# Fracture Fusion on Fast‐Forward: Locally Administered Deferoxamine Significantly Enhances Fracture Healing in Animal Models: A Systematic Review and Meta‐Analysis

**DOI:** 10.1002/advs.202413290

**Published:** 2025-01-22

**Authors:** Daniel Müller, Jens Klotsche, Magdalena B. Kosik, Carsten Perka, Frank Buttgereit, Paula Hoff, Timo Gaber

**Affiliations:** ^1^ Department of Rheumatology and Clinical Immunology Charité – Universitätsmedizin Berlin, corporate member of Freie Universität Berlin and Humboldt‐Universität zu Berlin 10117 Berlin Germany; ^2^ Deutsches Rheumaforschungszentrum Berlin (DRFZ) a Leibniz Institute 10117 Berlin Germany; ^3^ Charité – Universitätsmedizin Berlin, corporate member of Freie Universität Berlin and Humboldt‐Universität zu Berlin CharitéCenter for Orthopedics und Traumatology 10117 Berlin Germany; ^4^ MVZ Endokrinologikum Berlin am Gendarmenmarkt 10117 Berlin Germany

**Keywords:** µCt, bone regeneration, deferoxamine, mouse model, rat model

## Abstract

Fractures, with a yearly incidence of 1.2%, can lead to healing complications in up to 10% of cases. The angiogenic stimulant deferoxamine (DFO) is recognized for enhancing bone healing when administered into the fracture gap. This systematic review with meta‐analysis investigates the effect of local DFO application on bone healing in rat and mouse models. EMBASE, MEDLINE (PubMed), and Web of Science are systematically searched in January 2024. The study is prospectively registered in PROSPERO (CRD42024492533), and the SYRCLE tool is used to assess study quality and risk of bias. Outcome values contain the primary endpoint bone volume fraction (BV/TV) as well as the secondary endpoints bone volume, tissue volume, bone mineral density, trabecular separation, trabecular thickness, vessel formation and the mechanical properties, assessed by µCT, angiography and mechanical strength tests. Out of 21 included studies, 18 qualify for meta‐analysis, involving 539 animals. DFO‐treated groups exhibit significantly higher BV/TV values (*p* < 0.0001) compared to controls, with similarly significant improvements in secondary outcomes. These findings highlight the substantial benefit of DFO in promoting bone healing, especially after radiotherapy. Rapid clinical implementation is recommended to help patients at high risk of fracture healing complications.

## Graphical abstract

Fractures affect 1.2% of individuals annually, with healing complications in up to 10% of cases. This systematic review and meta‐analysis evaluates locally administered deferoxamine (DFO), an angiogenic stimulant, in rat and mouse fracture models. DFO significantly enhances vascularization, bone formation, mechanical properties, and overall bone quality at fracture sites, underscoring its strong potential for clinical fracture therapy.

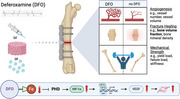

## 1 Introduction

Bone fractures, with a person‐yearly incidence of 1.2%,^[^ ^1^ ^]^ are prevalent injuries that the majority of people will experience at least once in their lifetime. Despite tremendous advancements in surgical techniques and the development of surgical materials, up to 10% of fracture patients still develop impaired bone regeneration or even so‐called non‐unions^[^ ^2^ ^]^ Standard treatments for fractures typically involve reduction, splinting, and the use of external fixators or plate and screw systems for stabilization, along with anticoagulation and analgetics including nonsteroidal anti‐inflammatory drugs (NSAIDs) to prevent thromboembolic events and manage pain. There are currently no reliable and well‐approved drug therapies specifically designed to enhance the primary process of fracture healing.^[^ ^3^ ^]^

This systematic review investigates DFO, also known as Desferal or Desferrioxamine, a drug with potential benefits in fracture healing as demonstrated in various animal studies.

DFO promotes angiogenesis and fracture healing by mimicking hypoxic conditions and activating the hypoxia‐inducible factor (HIF)‐1α/vascular endothelial growth factor (VEGF) pathway through iron chelation.^[^ ^4^ ^]^ The presence of DFO reduces intracellular iron levels, which act as a cofactor necessary for prolyl hydroxylase (PHD) activity. Inactivation of PHD prevents ubiquitination of HIF‐1α and, therefore, proteasomal degradation. Consequently, HIF‐1α accumulates in the nucleus and forms the HIF‐1 heterodimer with HIF‐1β, which then binds to the hypoxia‐responsive element (HRE) on the DNA. This activation leads to the transcription of genes involved in angiogenesis, including VEGF mRNA expression and protein production. Moreover, DFO reduces apoptosis by decreasing the formation of free radicals and reactive oxygen species (ROS) catalyzed by iron.^[^ ^5^ ^]^ The improved angiogenesis leads to enhanced transport of essential substances like oxygen, nutrients, metabolites, hormones, growth factors, and neurotransmitters to and from the regenerative area, supporting osteogenesis.^[^ ^6^ ^]^

Fracture healing involves complex physiological processes to restore bone function and is classified into primary and secondary healing. Primary bone healing occurs under conditions of absolute stability, with aligned bone ends and minimal strain, leading to no callus formation. In contrast, secondary healing involves non‐rigid fixation modalities, higher mechanical strain, and a larger fracture gap (>1mm), resulting in callus formation.^[^ ^7^, ^8^ ^]^

Indirect (secondary) fracture healing is the predominant form occurring,^[^ ^9^ ^]^ which was also observed in the animal studies we reviewed due to the described fracture gaps and mechanical stress.

DFO is FDA‐approved for treating acute and chronic iron overload and as an off‐label therapy for aluminum toxicity in patients with chronic kidney disease.^[^ ^10^ ^]^ Existing systematic reviews and meta‐analyses primarily discuss DFO's role in managing iron overload in conditions like thalassemia,^[^ ^11^, ^12^ ^]^ sickle cell anemia,^[^ ^11^, ^13^ ^]^ or intracerebral hemorrhage.^[^ ^14^, ^15^ ^]^

To the best of our knowledge, this systematic review and meta‐analysis is the first thorough approach to investigate the question: “Does the local application of Deferoxamine into the fracture gap enhance bone healing in animal models?”. The main purpose of this research is to explore the potential of DFO as a therapeutic agent in fracture management.

## 2 Results

### 2.1 Literature Search

**Figure 1:**
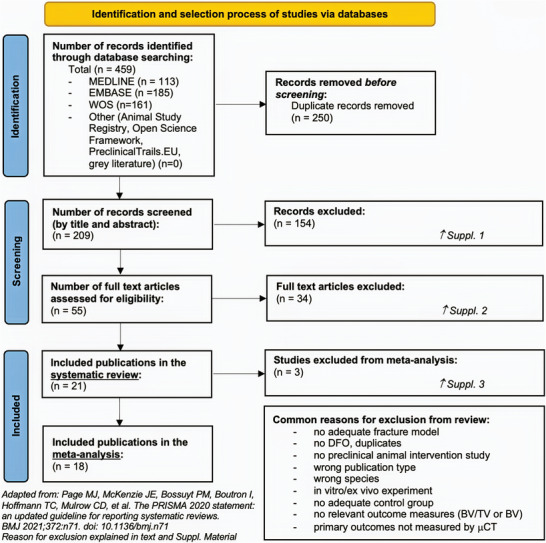
Prisma flow chart.

Our comprehensive systematic literature search initially identified a total of 459 potentially relevant studies for inclusion (**Figure** **1**). Of these, 250 were removed due to duplication, and an additional 188 were excluded based on their titles, abstracts, and full texts for not meeting the predefined inclusion criteria. No studies were excluded solely on the basis of language restrictions or for reasons other than those listed (Figure 1; Tables S1–S3, Supporting Information). Further exploration, including searches in recognized study registries and the application of MeSH and Emtree terms, did not uncover any new pertinent studies.

After a thorough screening process, 21 studies investigating the effect of DFO on fracture healing were deemed suitable for the systematic review.^[^ ^16^, ^17^, ^18^, ^19^, ^20^, ^21^, ^22^, ^23^, ^24^, ^25^, ^26^, ^27^, ^28^, ^29^, ^30^, ^31^, ^32^, ^33^, ^34^, ^35^, ^36^ ^]^ For the subsequent meta‐analysis, 3 publications were excluded due to incomplete or insufficient data regarding participant numbers (Geng, Shen, Stewart) or inadequate reporting of outcome measures (Stewart),^[^ ^23^, ^30^, ^31^ ^]^ culminating in 18 studies being incorporated into the statistical meta‐analysis.^[^ ^16^, ^17^, ^18^, ^19^, ^20^, ^21^, ^22^, ^24^, ^25^, ^26^, ^27^, ^28^, ^29^, ^32^, ^33^, ^34^, ^35^, ^36^ ^]^ Comprehensive details of the excluded studies and the specific reasons for their exclusion are documented in the Supplementary Table1 available online (Tables S1–S3).

### 2.2 Search Results and Study Characteristics

In our systematic review, 21 animal studies were evaluated for eligibility.^[^ ^16^, ^17^, ^18^, ^19^, ^20^, ^21^, ^22^, ^23^, ^24^, ^25^, ^26^, ^27^, ^28^, ^29^, ^30^, ^31^, ^32^, ^33^, ^34^, ^35^, ^36^ ^]^ Of these, 18 studies (85.71%) utilized rat models,^[^ ^16^, ^17^, ^18^, ^19^, ^20^, ^21^, ^22^, ^23^, ^24^, ^25^, ^27^, ^28^, ^29^, ^31^, ^33^, ^34^, ^35^, ^36^ ^]^ while 3 studies (14.29%) employed mouse models.^[^ ^26^, ^30^, ^32^ ^]^ The specific strains included Sprague‐Dawley (SD) rats (16 studies),^[^ ^16^, ^17^, ^18^, ^19^, ^20^, ^21^, ^22^, ^23^, ^24^, ^25^, ^27^, ^28^, ^33^, ^34^, ^35^, ^36^ ^]^ Norway rats (1 study),^[^ ^31^ ^]^ Fischer rats (1 study),^[^ ^29^ ^]^ and C57BL/6N mice (3 studies)^[^ ^26^, ^30^, ^32^ ^]^ (**Table** **1**).

**Table 1:** Study characteristics.

Study			Subjects				Intervention					
Author	Year	Country	Species and Strain	Sex [M/F]	Age	Weight	Disease Model	Fracture site	Type of Fracture Defect Model	DFO Application route	Dose + Frequency	Study Groups
Cheng^[^ ^16^ ^]^	2020	China	SD‐rats	M	8 weeks	__	healthy	calvaria	critical‐size‐full thickness bone hole (Dia: 5mm)	defect filled with injectable composite hydrogels with DFO	filling with DFO (120 µmol/L) in surgery	6 groups: blank (no filling), SNF/HA, SNF‐D/HA, SNF‐D‐B/HA, SNF‐D‐B/HA‐B, and SNF‐D/HA‐B
Cui^[^ ^17^ ^]^	2022	China	SD‐rats	__	6 weeks	__	healthy	calvaria	full‐thickness drill hole (Dia: 5 mm)	Electrospinning fibrous mats (EFMs) loaded with DFO	DFO filling (100 µM) in surgery	5 groups: BLANK (no implant), PCL, DFO, DEX, DFO + DEX
Donneys; Ahsan^[^ ^18^ ^]^	2013	USA	SD‐rats	M	12 weeks	400 g	radiotherapy (X‐groups)	mandibule	vertical osteotomy + external fixation (2 mm gap)	localized injections of DFO into the fracture callus (XFxDFO)	DFO (200 µM DFO in 300 µL NS) every other day for a total of 5 doses on POD 4–12	3 groups: Fx, XFx, XFxDFO
Donneys; Deshpande^[^ ^19^ ^]^	2013	USA	SD‐rats	M	12 weeks	400 g	healthy	mandibule	osteotomy + external fixation + DO (‐> 5.1 mm gap)	DFO injected directly into distraction gap (Exp. Groups)	DFO (200 µM in 300 µL NS) every other day POD 4 ‐ POD 12	6 Groups: Control 14d/21d/28d, Experimental 14d/21d/28d
Donneys; Yang^[^ ^20^ ^]^	2019	USA	SD‐rats	M	12 weeks	400 g	radiotherapy (X‐groups)	mandibule	vertical osteotomy + external fixation (2 mm gap)	DFO local injection directly into fracture site (iDFO); HA‐DFO‐scaffold implanted (HA‐DFO)	DFO (200 µM in 300 µL NS) every other day POD 4 ‐ POD 12 (iDFO group); DFO dose loaded on HA scaffold ≈ 1000 µM (≙ 5 DFO‐injections (200 µM each)	4 groups: Fx, XFx, iDFO, HA‐DFO
Fan^[^ ^21^ ^]^	2022	China	SD‐rats	M	8 weeks	250–300 g	healthy	distal femur	injury hole (Dia:3 mm; height: 5 mm)	fracture filled with DFO‐laden composite scaffolds (SF/HA‐DFO‐ groups)	DFO (120 µM) added to a scaffold with HA and SF (SF/HA‐DFO‐R)	6 groups: Blank, SF/HA‐R, SF/HA‐DFO‐R, SF/HA‐DFO‐A5, SF/HA‐DFO‐A10, SF/HA‐DFOA15.
Felice^[^ ^22^ ^]^	2013	USA	SD‐rats	M	__	__	radiotherapy (XRT‐ groups)	madibule	osteotomy + external fixation + DO (‐> 5.1 mm gap)	DFO locally injected (DO‐XRT‐DFO)	5 separate injections of DFO (300 µL) every other day starting on POD 4	3 groups: DO‐Control, DO‐XRT, DO‐XRT‐DFO
Geng^[^ ^23^ ^]^	2021	China	SD‐rats	M	4 weeks	__	healthy	calvaria	critical‐sized defect holes (Dia: 5 mm)	DFO‐loaded scaffold implantation	loading content of DFO in the scaffold 0.96 µg mg^−1^	6 groups: 1) control group (without scaffold), 2) PGSLP 3) MGP, 4) NGP, (5) DFO@MGP, (6) DFO@NGP scaffolds
Hou^[^ ^24^ ^]^	2023	China	SD‐rats	M	58 weeks	250 ‐ 300 g	healthy	femoral condyle	drill hole (Dia: 3 mm; depth: 5 mm)	DFO‐laoded cryogels implanted	20 mg DFO loaded into cryogels	5 groups: blank control (saline), SNF6, SNF10, SNF6‐D, SNF10‐D
Jia^[^ ^25^ ^]^	2016	China	SD‐rats	F	6 weeks	__	Osteoporosis: ovariectomy (OVX)	femur	critical‐sized drill hole (Dia: 2 mm, bicortical channel)	filling with DFO‐loaded scaffold (PLGA‐DFO group)	5 µL solution (containing 2 µg DFO) dropped into each PLGA scaffold	3 groups: control (no scaffold), PLGA, PLGA+DFO
Lang^[^ ^26^ ^]^	2022	Germany	C57BL/6N mice	F	12 weeks	20 ‐ 25 g	healthy	femur	Osteotomy (0.7 mm gap) + external fixator+ scaffold for delayed healing model	DFO locally applied by absorbable bovine Col‐I scaffold (ACS) into fracture gap	solved DFO (250 µM) applied on the ACS of treatment groups	4 groups: ACS (control; absorbable Col I‐based scaffolds), ACS+MIF, ACS+DFO, ACS+MIF/DFO
Li^[^ ^27^ ^]^	2023	China	SD‐rats	M	8 weeks	300 ± 50 g	healthy	femoral condyle	cylindrical drill hole defect (Dia: 3 mm; depth: 6 mm)	defects injected with the LCFS with different components (incl. DFO)	DFO stock solution was 50 mg mL^−1^ in distilled water	5 groups: Control (NS injections), LCFS, D@LCFS, S@LCFS, SD@LCFS
Liu^[^ ^28^ ^]^	2022	China	SD‐rats	M	adult	420 g	healthy	femur mid‐diaphysis	transverse osteotomy + external fixator + DO (‐> 5.0 mm gap)	DFO solution directly injected into distraction zone	DFO (200 µM in 300 µL NS) injection every other day from POD 16 ‐ 26	2 groups: Control (NS injections); Group1 (DFO injection)
Matsumoto^[^ ^29^ ^]^	2015	Japan	Fischer rats	F	12 weeks	140–150 g	healthy	tibial diaphysis	full‐thickness unicortical drill hole (Dia. 0.7 mm)	local injection of DFO to defect periphery	20 µL of DFO (200 µmol L^−1^) on alternate days from POD 1; POD 5 ‐> 2 doses, POD 10 ‐> 5 doses	6 groups: HU5, HU10, DFOHU5, DFOHU10, WB5, WB10
Shen^[^ ^30^ ^]^	2009	USA, China	C57BL/6 mice	M	8 weeks	__	healthy	midshaft femur	bending fracture + intramedullary pin	local DFO injection at the fracture site	20 µL of DFO (200 µM) every other day for 5 doses	3 groups: Control (NS injections), DFO, DMOG
Stewart^[^ ^31^ ^]^	2011	USA	Brown Norway rats	__	13 weeks	__	healthy	femur	Segmental defect (5 mm gap) + fixation with intramedullary Kirschner wire	DFO filled through DCPD cement portals of implanted PPF/TCP scaffolds	DFO (30 µL of a 400 µM solution)	5 groups: Control (NS injections), DFO, low dose (5µg) rh‐BMP‐2, combined DFO + low dose rhBMP‐2, rh‐BMP‐2 (10µg)
Wan^[^ ^32^ ^]^	2008	USA	C57BL/6 mice	__	8 weeks	__	healthy	left tibia	osteotomy + external fixator + DO (‐> 1.5 mm gap)	DFO injection directly into the distraction gap	injections with 200 µM DFO in distraction gap every other day from days 7 to 17 for a total of 5 doses	2 groups: Control (NS injection), DFO
Wei^[^ ^33^ ^]^	2022	China	SD‐rats	F	13 weeks	230–270 g	Osteoporosis: ovariectomy (OVX)	femoral condyle	cylindrical hole (Dia: 5 mm; length: 5 mm)	DFO‐loaded scaffolds applied into bone holes	each modified Mg‐TCP scaffold immersed in 0.15 mL of DFO solution (containing 0.1 mg DFO)	4 groups: OVX, OVX+TCP, OVX+ Mg‐TCP, OVX+DFO/Mg‐TCP
Yan^[^ ^34^ ^]^	2019	China	SD‐rats	M	8 weeks	300–350 g	healthy	femoral condyle	drill hole (Dia: 3 mm; bicortical channel)	scaffolds loaded with ≈0,37 mg DFO	scaffolds loaded with ≈0,37 mg DFO	4 groups: 1) Control (no scaffold), 2) PCL scaffolds, 3) PCH scaffolds, 4) PCD scaffolds
Zeng^[^ ^35^ ^]^	2022	China	SD‐rats	M	8 weeks	300–330 g	healthy	middle femur	critical segmental bone defect + internal fixation plate (5 mm gap)	filling material with DFO directly injected into defect	2.5 mL of diff. conc. (0, 30, 60, 120 µM) of DFO solution absorbed by 1 g GM (≙ 0, 42.05, 84.10, 168.20 µg DFO per 1 g GM)	5 groups: Control (no material), DFO‐0 (blank GMs hydrogel), DFO‐ 30, DFO‐60, and DFO‐120.
Zhao^[^ ^36^ ^]^	2023	China	SD‐rats	__	__	180–220 g	healthy	femoral condyle	critical‐size drill hole (Dia: 3 mm; depth: 3 mm)	DFO‐loaded scaffold implantation	Porous HA scaffold immersed in DFO solution (1 mg mL^−1^, several cycles for 30 min) followed by freeze‐drying for coating with DFO	5 groups: Control (no scaffold), H scaffold group, HD scaffold group (HD), HO scaffold group (HO); HOD scaffold group (HOD).

**Legend**: Dia = diameter; D = DFO = Deferoxamine; SD = Sprague Dawley; F = female; M = male; DO = distraction osteogenesis; POD = post‐operative day; NS = normal saline solution; **Cheng**: SNF/HA = silk nanofiber mixed with hydroxyapatite nanoparticles, B = BMP‐2 = bone morphogenic protein 2; **Cui**: PCL = polycaprolactone, DEX = dexamethasone; **Donneys, Ahsan**: Fx = fracture; **Donneys, Yang**: HA = hyaluronic acid; i = injected; **Fan**: SF = silk fibroin, HA = hydroxyapatite, A5/A10/A15 = treating time of scaffold in electric field during fabrication; R = scaffold not treated in electric field during fabrication; **Geng**: PGSLP = poly (glycerol‐co‐sebacic acid‐co‐L‐lactic acid‐co‐polyethylene glycol), MGP = macroporous structured gelatin/PGSLP, NGP = nanofibrous structured gelatin/PGSLP; **Hou**: SNF6/10 = silk nanofiber concentrations of 6 or 10%; **Jia**: PLGA = poly(Lactic‐co‐glycolic acid); **Lang**: ACS = absorbable bovine Col‐I scaffold, MIF = macrophage migration inhibitory factor; **Li**: LCFS = liquid crystal formulation system, S = simvastatin; **Matsumoto**: HU5/10 = hindlimb unloading for 5 or 10 days, WB5/10 = weight bearing for 5 or 10 days; **Shen**: DMOG = dimethyloxalylglycine; **Stewart**: DCPD = dicalcium phosphate dihydrate (cement portals), PPF/TCP = polypropylene fumarate/tricalcium phosphate, rh‐BMP2 = recombinant human bone morphogenetic protein‐2; **Wei**: Mg = Magnesium, TCP = β‐tricalcium phosphate; **Yan**: PCL = Polycaprolactone, PCH = aminated PCL, PCD = DFO loaded PCH; **Zeng**: GM = gelatin microspheres; **Zhao**: HA = H = hydroxyapatite, HD = HA + DFO, HO = HA + OKGN (oxidized konjac glucomannan)

Various bone defect and bone regeneration models were used, including different sizes of defects in long bones (*n* = 14)^[^ ^21^, ^24^, ^25^, ^26^, ^27^, ^28^, ^29^, ^30^, ^31^, ^32^, ^33^, ^34^, ^35^, ^36^ ^]^ like femur (*n* = 12)^[^ ^21^, ^24^, ^25^, ^26^, ^27^, ^28^, ^30^, ^31^, ^33^, ^34^, ^35^, ^36^ ^]^ and tibia (*n* = 2)^[^ ^29^, ^32^ ^]^ as well as other bones (*n* = 7)^[^ ^16^, ^17^, ^18^, ^19^, ^20^, ^22^, ^23^ ^]^ like mandible (*n* = 4)^[^ ^18^, ^19^, ^20^, ^22^ ^]^ and calvaria (*n *= 3).^[^ ^16^, ^17^, ^23^ ^]^ These fractures were surgically induced in different ways, either via drill hole (*n* = 11)^[^ ^16^, ^17^, ^21^, ^23^, ^24^, ^25^, ^27^, ^29^, ^33^, ^34^, ^36^ ^]^ or via osteotomy and surgical fixation, resulting in a fracture gap (*n* = 10).^[^ ^18^, ^19^, ^20^, ^22^, ^26^, ^28^, ^30^, ^31^, ^32^, ^35^ ^]^

A notable regeneration model was distraction osteogenesis, applied in 4 studies, where defects were gradually expanded over several experimental days to achieve a target bone gap size.^[^ ^19^, ^22^, ^28^, ^32^ ^]^ Additionally, some studies implemented disease models into their experiments, where they induced irradiated fractures with radiotherapy (*n* = 3)^[^ ^18^, ^20^, ^22^ ^]^ or osteoporosis with ovariectomy (*n* = 2).^[^ ^25^, ^33^ ^]^

For the local delivery of DFO into the fracture gap, 8 studies used direct injection,^[^ ^18^, ^19^, ^20^, ^22^, ^28^, ^29^, ^30^, ^32^ ^]^ while 13 studies employed advanced delivery systems being loaded with DFO, such as various scaffolds, composite hydrogels, cryogels, or fibrous mats, being implanted during surgery.^[^ ^16^, ^17^, ^21^, ^23^, ^24^, ^25^, ^26^, ^27^, ^31^, ^33^, ^34^, ^35^, ^36^ ^]^

The dose and administration frequency of DFO showed considerable variations across the studies. Precise details on DFO concentrations and dosages were challenging to standardize, due to the varied processing, loading, and release patterns of the DFO‐loaded vector systems employed in different studies.

Biocompatibilities and ‐characteristics of the intelligent vector systems were confirmed by in vitro experiments prior to their use in animal bone defects.

Variations of study characteristics were addressed within our subgroup analyses.

### 2.3 Study Results

Our primary endpoint, bone formation, was quantitatively assessed in all studies using µCT, providing data on BV/TV or BV. Secondary endpoints, including BMD, Tb.Sp., and Tb.Th., were evaluated in 11,^[^ ^18^, ^19^, ^20^, ^21^, ^22^, ^24^, ^27^, ^28^, ^33^, ^34^, ^36^ ^]^ 7,^[^ ^16^, ^21^, ^24^, ^25^, ^27^, ^33^, ^34^ ^]^ and 6^[^ ^16^, ^21^, ^25^, ^27^, ^33^, ^34^ ^]^ studies out of 21, respectively (**Table** **2**).

**Table 2:** Study results.

Study		Measurement		Outcome measures									
Author	Year	µCT device	Scanning parameters	Parameters measured by µCT						Time points	Compared groups: chosen control vs DFO group (study arm)	Study summary	Inclusion in Meta‐Analysis
				BV/TV	BV	TV	BMD	Tb.Sp.	Tb.Th.				
Cheng^[^ ^16^ ^]^	2020	SkyScan 1176, SkyScan, Aartselaar, Belgium	voltage 65 kV, current 385 µA, resolution 18 µm	X	X	(X)		X	X	4, 8, 12 weeks	SNF/HA vs. SNF‐D/HA	Osteoid regeneration improved with incorporation of DFO into hydrogels compared to the DFO free hydrogels.	Yes
Cui^[^ ^17^ ^]^	2022	scanning system (Quantum GX)	90 kV, 88 µA, 2 min standard scan model, 36 µm voxel size	X	X	(X)				8 weeks	PCL vs DFO (a) DEX vs DFO+DEX (b)	DFO+DEX group had the highest new bone formation in the defect area, with DEX and DFO groups following. No significant difference in BV and BV/TV between DFO and DEX groups seen.	Yes
Donneys; Ahsan^[^ ^18^ ^]^	2013	__	80 kVp, 80 mA and 1100 ms exposures, 392 projections taken at a 45‐micron voxel size	(X)	X	X	X			6 weeks (POD 40)	XFx vs XFxDFO	DFO significantly enhanced callus size, mineralization, and strength in mandibular fractures compared to those subjected to radiation, achieving levels comparable to non‐radiated controls. This indicates DFO's potential as a novel clinical treatment for pathologic fractures caused by radiation.	Yes
Donneys; Deshpande^[^ ^19^ ^]^	2013	__	80 kVp, 80 mA and 1100 ms exposures, 392 projections taken at a 45 µm voxel size	X			X			2, 3, 4 weeks	Control vs Experimental	Within the experimental group (DFO) BMD and BV/TV were significantly higher at 14 and 21 days post‐surgery when compared to the control group. After 28 days, these values consolidated and normalized to levels seen in fully healed bone.	Yes
Donneys; Yang^[^ ^20^ ^]^	2019	Micro‐CT (GE Healthcare Biosciences)	80 kVp, 80 mA and 1100 ms exposure at a resolution of 45 µm voxel size	X			X			6 weeks (POD 40)	XFx vs iDFO	DFO injections notably enhanced bone regeneration and mineralisation at the irradiated fracture sites.	Yes
Fan^[^ ^21^ ^]^	2022	Bruker Micro‐CT, Skyscan 1276 system (Kontich, Belgium)	85 kV, 200 mA, 1 mm Al filter, integration time 384 ms, voxel size 10 µm, medium resolution	X			X	X	X	4, 12 weeks	SF/HA‐R vs SF/HA‐DFO‐R	The regulated release of DFO from loaded composite scaffolds enhanced the regeneration of bone tissue.	Yes
Felice^[^ ^22^ ^]^	2013	__	80 kVp, 80 mA and 1100 ms exposures, 392 projections taken at a 45 µm voxel size	X			X			6 weeks (POD 40)	DO‐XRT vs DO‐XRT‐DFO	DFO effectively improves bone healing and structural integrity in radiation‐impaired distraction osteogenesis in murine mandibles, suggesting its utility in reconstructions for head and neck cancer patients.	Yes
Geng^[^ ^23^ ^]^	2021	Bruker Micro‐CT, SkyScan 1176 (Belgium).	__	X						6, 12 weeks	MGP vs DFO@MGP (a) NGP vs DFO@NGP (b)	DFO‐loaded scaffolds significantly improved vascularization and bone healing. (NGP group was better than MGP group)	No (no n given)
Hou^[^ ^24^ ^]^	2023	Bruker Micro‐CT, Skyscan 1276 system (Kontich, Belgium)	85 kV, 200 mA, 1 mm Al filter, integration time 384 ms, voxel size 10 µm, medium resolution	X			X	X		4, 12 weeks	SNF6 vs SNF6‐D (a) SNF10 vs SNF10‐D (b)	Compared to silk bone materials without DFO, DFO‐loading improved osteogenic outcomes.	Yes
Jia^[^ ^25^ ^]^	2016	high‐resolution (16 µm) micro‐CT scanner (GE eXplore Locus SP)	isotropic voxel size 16 µm (high‐resolution)	X				X	X	2, 4 weeks	PLGA vs PLGA+DFO	DFO, when released from PLGA, effectively reverses osteoporosis‐related declines in bone and blood vessel formation.	Yes
Lang^[^ ^26^ ^]^	2022	SCANCO µCT Viva 40	70 KVp, 114 µA, 191 slices with isotropic voxel size of 10.5 µm	X	X	X				2 weeks 3 weeks	ACS vs ACS+DFO a) ACS+MIF vs ACS+MIF/DFO b) ACS vs ACS+DFO (a) ACS+MIF vs ACS+MIF/DFO (b)	DFO without MIF marginally improved disrupted bone regeneration, without significance. Data suggests DFO enhances healing via increased mineralization and angiogenesis. DFO and MIF combined showed no significant synergistic benefit.	Yes
Li^[^ ^27^ ^]^	2023	high‐resolution Micro‐CT (Skyscan 1276, Bruker, Germany)	90 kV, 270 mA, 550 ms, isometric resolution of 20 mm	X			X	X	X	4, 8 weeks	LCFS vs D@LCFS (a) S@LCFS vs SD@LCFS (b)	Measurements showed significant angiogenic and osteogenic effects from the sequential application of DFO and SIM released from LCFS, providing an effective treatment for bone defects.	Yes
Liu^[^ ^28^ ^]^	2022	BRUKER Micro‐CT, SkyScan 1176, Bruker Physik‐AG, Rheinstetten, Germany	65 kV, 385 mA for 340 ms; Al + C + Cu filter, voxel size 18 mm	X			X			6 weeks	Control vs Group1	Therapy with DFO significantly improved bone regeneration during the consolidation phase.	Yes
Matsumoto^[^ ^29^ ^]^	2015	__	high‐resolution µCT using monochromatic synchrotron radiation subtraction just above (18.1 keV) and below (17.9 keV)	X						1 week (POD 5), 2 weeks (POD 10)	HU5 vs DFOHU5 HU10 vs DFOHU10	DFO can stimulate angiogenesis to counteract the negative effects of hindlimb unloading (HU) on early‐stage bone repair in rat cortical bone. However, the current dosage of DFO did not fully achieve the same bone repair levels seen in weight‐bearing (WB) rats.	Yes
Shen^[^ ^30^ ^]^	2009	µCT‐40 system (Scanco Medical, Bassersdorf, Switzerland)	12 µm voxel size, 250 slices, segmentation threshold of 204	(X)	X	X				4 weeks	Saline vs DFO	After 28 days µCT scans showed significantly higher BV and TV in the fracture callus of the DFO group versus saline, indicating HIF activation as an effective strategy for boosting vascularity and bone growth after skeletal trauma.	No (imprecise n)
Stewart^[^ ^31^ ^]^	2011	Scanco 40 µCT (Scanco Medical)	16 µm isotropic voxel size, 500 slices, segmentation threshold of 270		X					12 weeks	Saline vs DFO (a) low dose rh‐BMP‐2 vs combined DFO + low dose rh‐BMP‐2 (b)	DFO enhanced vascularization and stiffness of bone in segmental defects. Mean bone volume was increased in DFO treatment groups compared to saline control, but did not reach statistical significance (*p* = 0.18).	No (imprecise outcome data and n)
Wan^[^ ^32^ ^]^	2008	µCT‐40 system (Scanco Medical)	__	X	X	(X)				4 weeks (POD 31)	Saline vs DFO	DFO treatment significantly improved BV and BV/TV, indicating enhanced bone regeneration and angiogenesis, compared to controls.	Yes
Wei^[^ ^33^ ^]^	2022	high‐resolution (16 µm) micro‐CT scanner (GE eXplore Locus SP)	isotropic voxel size 16 µm (high‐resolution)	X			X	X	X	12 weeks	OVX+Mg‐TCP vs OVX+DFO/Mg‐TCP	Local DFO/Mg‐TCP implants significantly enhanced bone regeneration and mineralization in osteoporotic rats, outperforming DFO‐free implants.	Yes
Yan^[^ ^34^ ^]^	2019	high‐resolution micro‐CT (Scanco Medical, Switzerland)	70 kV, 130 µA radiation source with 0.5 mm aluminum filter, isometric resolution of 20 µm	X			X	X	X	2, 4 weeks	PCH vs PCD	DFO in PCD scaffolds significantly enhanced vascularity, bone growth, and osseointegration in a rat model with weight‐bearing bone defects, demonstrating promising results for large bone defect repair.	Yes
Zeng^[^ ^35^ ^]^	2022	Micro‐CT SkyScan 1276, Bruker, Billerica, USA	100 kV, 80 µA, 18 µm pixel resolution	X						12 weeks	DFO‐0 vs DFO‐30 (a) vs DFO‐60 (b) vs DFO‐120 (c)	BV/TV indicated the highest bone formation in the DFO‐60 group, showing superiority over all the other groups. The results highlight the importance of proper dosing.	Yes
Zhao^[^ ^36^ ^]^	2023	Micro‐CT (Skyscan 1076, Skyscan company, USA)	70 kV, 110 mA, Al + Cu filter with resolution of 18 µm.	X			X			2, 8 weeks	H vs HD (a) HO vs HOD (b)	The application of DFO coating significantly stimulated angiogenesis, which enhanced the supply of nutrients essential for tissue regeneration and healing.	Yes

**Legend**: (X) not directly given but calculated from other given values; D = DFO = Deferoxamine; DO = distraction osteogenesis; POD = post‐operative day; **Cheng**: SNF/HA = silk nanofiber mixed with hydroxyapatite nanoparticles; **Cui**: PCL = polycaprolactone, DEX = dexamethasone; **Donneys, Ahsan**: Fx = fracture; **Yang**: i = injected; **Fan**: SF = silk fibroin, HA = hydroxyapatite, R = scaffold not treated in electric field during fabrication; **Geng**: PGSLP = poly (glycerol‐co‐sebacic acid‐co‐L‐lactic acid‐co‐polyethylene glycol), MGP = macroporous structured gelatin/PGSLP, NGP = nanofibrous structured gelatin/PGSLP; **Hou**: SNF6/10 = silk nanofiber concentrations of 6 or 10%; **Jia**: PLGA = poly(Lactic‐co‐glycolic acid); **Lang**: ACS = absorbable bovine Col‐I scaffold, MIF = macrophage migration inhibitory factor; **Li**: LCFS = liquid crystal formulation system, S = simvastatin; **Matsumoto**: HU5/10 = hindlimb unloading for 5 or 10 days, WB5/10 = weight bearing for 5 or 10 days; **Stewart**: rh‐BMP2 = recombinant human bone morphogenetic protein‐2; **Wei**: Mg = Magnesium, TCP = β‐tricalcium phosphate; **Yan**: PCL = Polycaprolactone, PCH = aminated PCL, PCD = DFO loaded PCH; **Zhao**: HA = H = hydroxyapatite, HD = HA + DFO, HO = HA + OKGN (oxidized konjac glucomannan)

To facilitate comparison, observation time points, if given in postoperative days (POD), were standardized to whole weeks, with the most measurements occurring at 2 (6 studies),^[^ ^19^, ^25^, ^26^, ^29^, ^34^, ^36^ ^]^ 4 (9 studies),^[^ ^16^, ^19^, ^21^, ^24^, ^25^, ^27^, ^30^, ^32^, ^34^ ^]^ 6 (5 studies),^[^ ^18^, ^20^, ^22^, ^23^, ^28^ ^]^ 8 (4 studies),^[^ ^16^, ^17^, ^27^, ^36^ ^]^ and 12 (7 studies)^[^ ^16^, ^21^, ^23^, ^24^, ^31^, ^33^, ^35^ ^]^ weeks.

The reliability of µCT scanning, our method for measuring outcomes such as BV/TV, BV, TV, BMD, Tb.Sp., and Tb.Th., is influenced by equipment specifications. While µCT is the gold standard for these measurements, offering high accuracy and precision, the quality is contingent upon scanning parameters such as voltage, current, and resolution. We acknowledge the given variability in µCT models and scanning settings as potential confounders.

### 2.4 Quality Assessment and Risk of Bias

**Figure 2:**
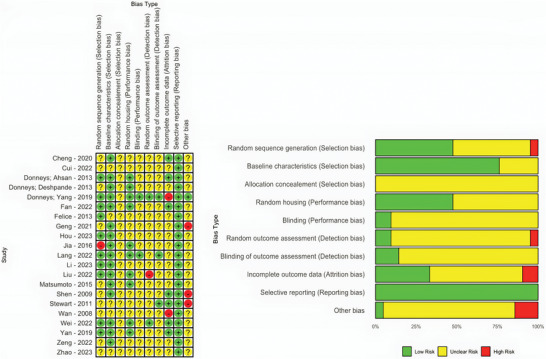
Risk of bias assessment for studies included in our systematic review. Adapted from the SYRCLE RoB tool. “+” = low risk; “?” = unclear risk; “‐” = high risk.

Nearly half (47.62%) of the included studies adequately described the randomization process for assigning subjects to control and various treatment groups.^[^ ^18^, ^20^, ^21^, ^22^, ^24^, ^26^, ^27^, ^28^, ^33^, ^34^ ^]^ Most of the studies (16/21) reported more than 3 of the 5 important baseline characteristics (species, strain, age, sex, weight) of the subjects.^[^ ^16^, ^18^, ^19^, ^20^, ^21^, ^23^, ^24^, ^25^, ^26^, ^27^, ^28^, ^29^, ^30^, ^33^, ^34^, ^35^ ^]^(**Figure** **2**).

None of the studies disclosed whether the results of randomization were concealed until the intervention commenced (allocation concealment). Additionally, 47.62% of the studies provided specifics on animal housing and husbandry conditions.^[^ ^18^, ^19^, ^20^, ^21^, ^25^, ^26^, ^28^, ^29^, ^33^, ^34^ ^]^

Limited information was given on random assignment to measurement groups and blinding of outcome assessment, noted in only 9.52%.^[^ ^20^, ^33^ ^]^ and 14.29%.^[^ ^20^, ^26^, ^31^ ^]^ of studies, respectively, indicating a high risk of detection bias.

In the “other bias” category, we excluded 3 studies (Geng, Shen, and Stewart) judged with “high risk” because Geng provided no and Shen and Stewart did not have enough information about the subject number.^[^ ^23^, ^30^, ^31^ ^]^ The overall risk of bias for most studies was categorized as “unclear”.

Lang et al. was the only study referring to the “3Rs” principle (replacement, refinement, reduction) for experiments with laboratory animals.^[^ ^37^ ^]^

Little information was given about applied blinding methods and handling dropouts (incomplete outcome data).

6 studies (Cui, Felice, (Geng), Wan, Zeng, Zhao) were considered low‐quality studies (<3 positive judgments), which we further addressed in our sensitivity analysis.^[^ ^17^, ^22^, ^23^, ^32^, ^35^, ^36^ ^]^

### 2.5 The Overall Effect of BV/TV and Heterogeneity

**Figure 3:**
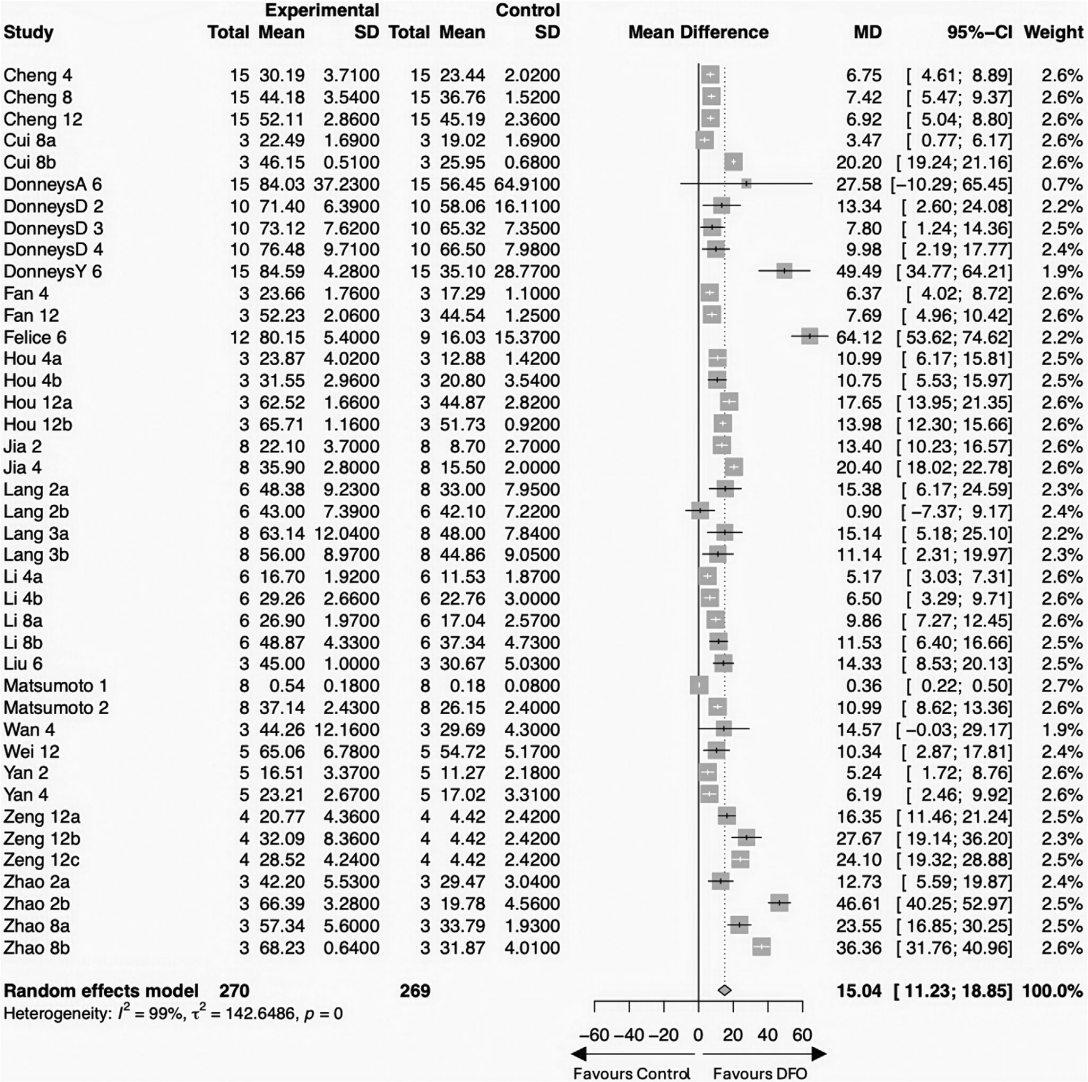
Forest plot for the overall comparison of DFO‐groups versus DFO‐free groups: The forest plot displays the relative weight of the individual studies, the estimates of treatment effect expressed as mean differences (MD), and 95% confidence intervals (CI). The diamond at the bottom line indicates the pooled estimate and its 95% CI. References on the left give study names. Study names are designated by the “Lead Author's Name” followed by the “Initial of a Co‐Author (capitalized) if the lead author has contributed multiple studies”, then the “measurement time point after fracture surgery (rounded) in weeks”, and finally, a “lowercase letter to differentiate multiple study arms within a single study”.

18 studies were finally included in the meta‐analysis. We used the extracted values of the investigated DFO group as well as the respective adequate comparison group, which had the identical intervention except DFO (**Figure** **3**).

In the analysis, the 18 studies provided 41 directly comparable experimental‐control group pairs, encompassing a total of 270 subjects in experimental groups and 269 in control groups.^[^ ^16^, ^17^, ^18^, ^19^, ^20^, ^21^, ^22^, ^24^, ^25^, ^26^, ^27^, ^28^, ^29^, ^32^, ^33^, ^34^, ^35^, ^36^ ^]^ In the forest plot, a confidence interval (CI) crossing the line of no effect (indicating negative CI values) denotes a non‐significant outcome. Findings to the right side favor the DFO‐treated groups, suggesting DFO's efficacy over controls. Notably, only 3 of the 41 pairs (≈7%) displayed non‐significant outcomes,^[^ ^18^, ^26^, ^32^ ^]^ with the remaining pairs showing significant benefits of DFO treatment.

The pooled effect size, as calculated under a random effects model due to the high level of heterogeneity (I^2^ = 99%), showed a significant MD of 15.04% (95% CI: 11.23; 18.85), supporting the hypothesis that DFO treatment is likely to result in a significant increase in BV/TV compared to controls (*p* < 0.0001).

It is imperative to note the high heterogeneity observed, which suggests variation in the effect sizes across studies. This variation could be due to differences in study design, sample sizes, duration of DFO treatment, or other study‐specific factors.

### 2.6 Subgroup Analysis by Categories

Despite subgroup analyses, the high heterogeneity (I^2^) persisted. Hence, the random effects model was consistently applied to accommodate the variability across studies.

#### 2.6.1 Health State

**Figure 4:**
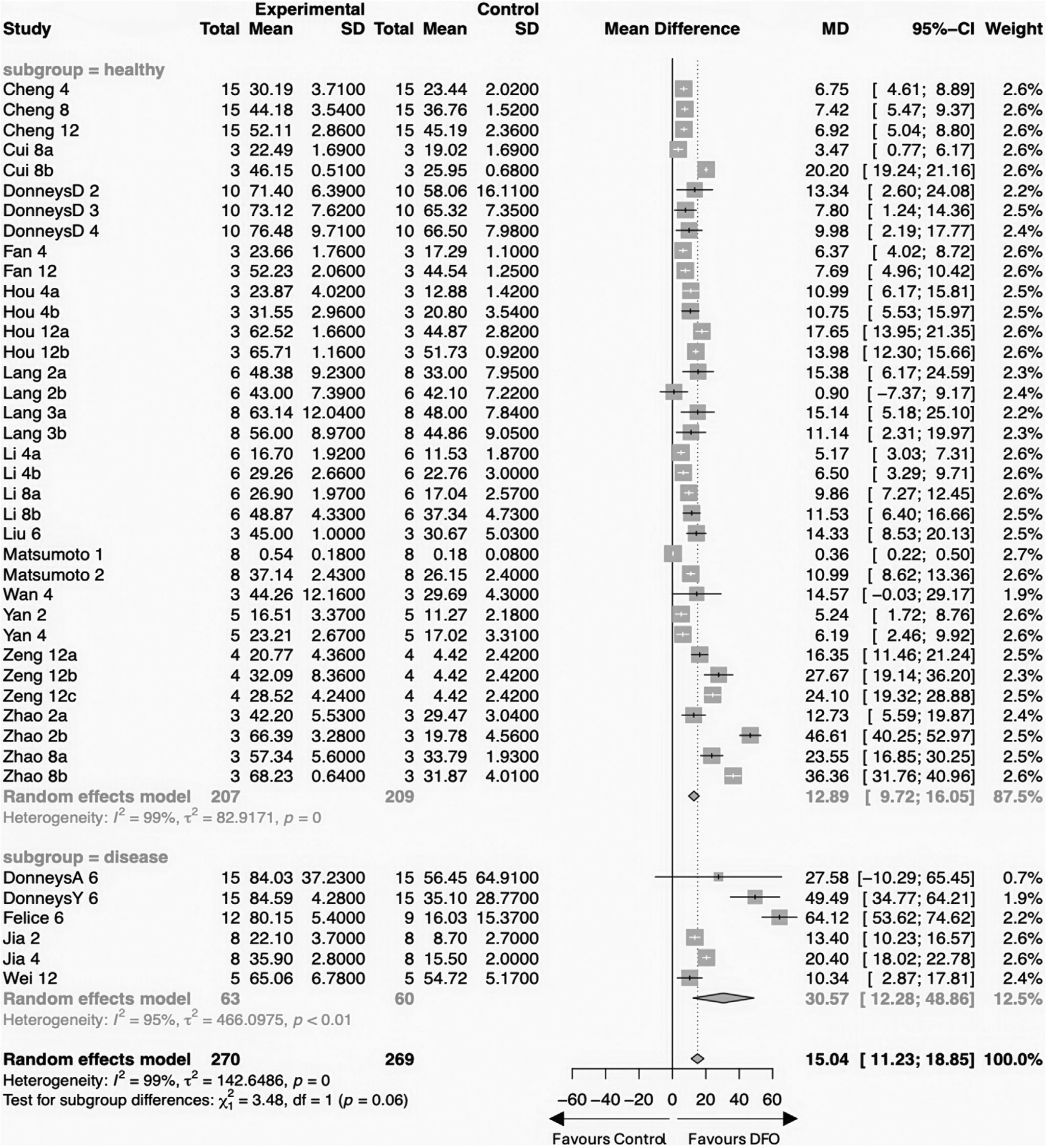
Forest plot for analyzing the subgroups of healthy bone and disease models.

Five studies (DonneysA, DonneysY, Felice, Jia, Wei) involved disease models impacting fracture healing^[^ ^18^, ^20^, ^22^, ^25^, ^33^ ^]^ Consequently, we conducted a subgroup analysis comparing these with studies on healthy bone to assess differential effects (**Figure** **4**).

The positive effect of DFO was notably more pronounced in the disease model subgroups, particularly in studies involving irradiated fractures in the mandible (DonneysA, DonneysY, Felice).^[^ ^18^, ^20^, ^22^ ^]^ Although significant effects were also observed in osteoporosis models induced by ovariectomy (Jia, Wei), they were less pronounced.^[^ ^25^, ^33^ ^]^

The disease model subgroup shows a significant MD that was over double that of the healthy bone subgroup, at 30.57% (CI: 12.28; 48.86) compared to 12.89% (CI: 9.72; 16.05), respectively. Despite this substantial difference, the subgroup variation was not statistically significant (*p* = 0.06), partly attributed to the broad CI in the disease subgroup.

The larger number of studies involving healthy bone models contributed to their greater overall weight (87.5%) in the analysis, diminishing the relative impact of the disease model studies (12.5% weight). This resulted in a minimal overall mean difference shift of 2.15%, which was not statistically significant.

Despite the modest overall change, we acknowledged the impact of disease model studies on our findings. These were carefully considered, particularly in the more detailed subgroup analyses where fewer studies were aggregated, thus potentially amplifying their influence.

Investigating the radiotherapy‐focused studies of the disease subgroup exclusively, there was a significantly higher MD compared to those studies with healthy or osteoporosis models, suggesting DFO is particularly effective in irradiated fractures. However, the broader confidence intervals in radiotherapy studies indicate a less precise estimation of the true effect, and the small number of studies makes it less robust. (Figure S1, Supporting Information).

#### 2.6.2 Bone Type

The studies included in our analysis focused on different types of bones, categorized into cranial bones (Cheng, Cui, DonneysA, DonnesD, DonneysY, Felice)^[^ ^16^, ^17^, ^18^, ^19^, ^20^, ^22^ ^]^ and long bones (Fan, Hou, Jia, Lang, Li, Liu, Matsumoto, Wan, Wei, Yan, Zeng, Zhao).^[^ ^21^, ^24^, ^25^, ^26^, ^27^, ^28^, ^29^, ^32^, ^33^, ^34^, ^35^, ^36^ ^]^ Specifically, Cheng and Cui examined the calvaria,^[^ ^16^, ^17^ ^]^ while DonneysA, DonnesD, DonneysY, and Felice focused on the mandible^[^ ^18^, ^19^, ^20^, ^22^ ^]^ (Figures S2.1 and S2.2, Supporting Information).

The forest plot analysis revealed that DFO's effects on BV/TV were slightly more pronounced in the cranial bone subgroup compared to the long bones, with an MD of 18.77% (CI: 7.01; 30.53) and 14.06% (CI: 10.52; 17.59), respectively, although the difference between these groups was not statistically significant (*p* = 0.45) (Figure S2.1, Supporting Information).

It is important to note that the cranial bone subgroup includes all studies that utilized radiotherapy models on the mandible, which are known to produce extreme values that significantly influence the pooled MD of the subgroup. To account for this, we conducted an additional analysis excluding all disease models, which shifted the advantage from the cranial to the long bones, with MDs of 9.32% (CI: 5.25; 13.38) and 13.98% (CI: 10.07; 17.88), respectively (Figure S2.2, Supporting Information). This adjustment did not result in a significant difference between the subgroups (*p* = 0.11), but did confirm the significant positive effect of DFO in both cranial and long bone groups compared to control groups.

#### 2.6.3 Application Route

In our analysis, we observed a variety of DFO application methods across the included studies, prompting us to compare the outcomes of studies utilizing advanced delivery systems such as scaffolds, hydrogels, and nanoparticles (referred to as the “vector” subgroup) against those employing direct injection of DFO solution without influencing auxiliary molecules (Figures S3.1 and S3.2, Supporting Information).

When considering all studies, including those with disease models, the direct injection approach exhibited a higher MD in BV/TV of 20.46% (CI: 7.57; 33.35) compared to 13.74% (CI: 10.32; 17.16) for the vector subgroup. However, the difference between these 2 application methods was not statistically significant (*p* = 0.32) (Figure S3.1, Supporting Information).

Upon excluding studies involving disease models, the dynamic changed, with the vector delivery systems showing a superior effect, evidenced by an MD of 13.63% (CI: 9.87; 17.39), compared to 9.16% (CI: 4.62; 13.69) for direct injection (Figure S3.2, Supporting Information). Despite this shift, both subgroups continued to demonstrate a significant positive impact of DFO on bone regeneration. Nevertheless, the disparity between the 2 application methods remained statistically non‐significant (*p* = 0.14).

#### 2.6.4 Type of Fracture Defect Model

The included studies employed various surgical techniques to induce fractures, ranging from drilling holes to creating fracture gaps via osteotomy that required surgical realignment of the bone ends for stabilization and healing (Figure S4.1,2, Supporting Information).

The drilled holes created larger gaps between bone edges yet facilitated bone formation from various directions and offered stable fracture conditions. Conversely, gap fractures, despite having shorter distances between bone ends, restrict bone growth to the 2 bone edges. Despite surgical stabilization, these fractures experience more movement at the fracture site, contributing to stress on the callus.

Some gap fractures underwent distraction osteogenesis (DO), a process in which the fracture gap was gradually extended over several days to a final gap length (as seen in studies like DonneysD, Felice, Liu, Wan).^[^ ^19^, ^22^, ^28^, ^32^ ^]^ DO did not demonstrate a significant difference in outcomes compared to other treatment groups.

When comparing the bone hole and gap subgroups, only a minor and non‐significant difference in pooled MDs of 7.6% was observed (*p* = 0.10), with the respective MDs being 12.58% (CI: 8.77; 16.38) and 20.18% (CI: 11.84; 28.52) (Figure S4.1, Supporting Information). This trend persisted after excluding studies that employed disease models, with nearly negligible differences in MD between the hole (12.30%, CI: 8.05; 16.55) and gap (14.39%, CI: 10.22; 18.55) groups (*p* = 0.49) (Figure S4.2, Supporting Information). Both groups exhibited significant benefits from DFO treatment, indicating that the type of the fracture defect model, whether a hole or a gap, does not significantly impact the efficacy of DFO, making it an effective treatment for various fracture types.

#### 2.6.5 Time Points

The time points for measuring BV/TV varied among the studies, with the most common assessments at 2, 4, 8, and 12 weeks after the start of the intervention period (Figure S5.1,2, Supporting Information). For consistency, measurements taken on postoperative days (POD) were rounded to full weeks. The observed alterations in MDs across these time points did not exhibit a consistent trend, potentially due to random variation and allocation, including the impact of outlier studies (Figure S5.1, Supporting Information). This observation did not change after the exclusion of the disease model studies (Figure S5.2, Supporting Information).

#### 2.6.6 Sex

In our included studies, male rats and mice were predominantly used, reflecting the common practice in research. Recognizing the potential influence of sex on outcomes, we conducted a subgroup analysis to compare results between male and female subjects (Figure S6.1,2, Supporting Information). Three of the 18 studies (Cui, Wan, Zhao)^[^ ^17^, ^32^, ^36^ ^]^ were excluded from this subgroup analysis due to insufficient information on the sex of the experimental animals.

The subgroup analysis revealed no significant difference between male and female subjects (*p* = 0.30), with a subgroup variation of 3.64% (Figure S6.1, Supporting Information). This difference further decreased to a marginal 2.46% (*p* = 0.42) if studies using disease models were excluded (Figure S6.2, Supporting Information).

The minimal and statistically insignificant subgroup difference confirms that both females and males derive equal benefit from local DFO therapy for fractures.

#### 2.6.7 Age

It is well‐established that younger organisms regenerate more efficiently than older ones. To evaluate whether DFO therapy exhibits differing effectiveness based on age, we conducted a subgroup analysis, categorizing the studies into 2 groups: young and old (Figure S7.1,2, Supporting Information). Using 8 weeks as the cutoff age, which approximately marks the end of puberty and the onset of adolescence in rats and mice,^[^ ^38^, ^39^ ^]^ animals aged ≤8 weeks were classified as young, while those >8 weeks were categorized as old. Two studies (Felice and Zhao)^[^ ^22^, ^36^ ^]^ were excluded from the analysis due to the absence of age data. One study (Liu et al.)^[^ ^28^ ^]^ described the animals' age only as “adult”, which we interpreted as referring to animals older than 8 weeks, and it was therefore included in the analysis.

The subgroup analysis demonstrated negligible differences between the 2 age groups. This was consistent across both the full dataset (Figure S7.1, Supporting Information) and the analysis limited to healthy animals (Figure S7.2, Supporting Information), with *p*‐values of 0.65 and 0.91, respectively.

These findings suggest that DFO therapy for fractures is equally effective across different age groups.

### 2.7 Results and Analysis of Secondary Endpoints

Our results consistently found that DFO was superior to the control groups within all our secondary endpoints.

Results of bone parameters:

In analyzing secondary endpoints, we explored additional bone parameters frequently assessed in the included studies. We were able to extract or calculate data for BV, TV, BMD, Tb.Sp., and Tb.Th. from 7,^[^ ^16^, ^17^, ^18^, ^26^, ^30^, ^31^, ^32^ ^]^ 6,^[^ ^16^, ^17^, ^18^, ^26^, ^30^, ^32^ ^]^ 11,^[^ ^18^, ^19^, ^20^, ^21^, ^22^, ^24^, ^27^, ^28^, ^33^, ^34^, ^36^ ^]^ 7,^[^ ^16^, ^21^, ^24^, ^25^, ^27^, ^33^, ^34^ ^]^ and 6^[^ ^16^, ^21^, ^25^, ^27^, ^33^, ^34^ ^]^ studies, respectively. Due to persistent high heterogeneity, the random effects model was consistently applied.

Regarding bone volume (BV), which is defined as the volume within the area of interest (AOI) being recognized as bone [in mm^3^], more than half of the study group pairs demonstrated a significant increase relative to their controls with an MD of 2.4 mm^3^ (CI: 0,49; 4.32) (Figure S8, Supporting Information).

The tissue volume parameter (TV), representing the total volume of the entire AOI [in mm^3^], did not seem to be remarkably affected by DFO treatment. The pooled effect size of the DFO groups indicated a slight but non‐significant increase in callus or tissue volume by 0.75 mm^3^ (CI: ‐0.19; 1.69) (Figure S9, Supporting Information).

Another bone parameter evaluating the bone's quality is bone mineral density (BMD), which refers to the amount of mineral matter per cubic centimeter of bone [in mg/cm^3^]. The analysis revealed a significant enhancement in BMD after DFO treatment, with an MD of 105.67 mg cm^−3^ (CI: 69.35; 141.99). This significant increase suggests that DFO markedly influences the mineralization of newly formed bone, potentially explainable by the improved delivery of minerals and nutrients to the fracture site, facilitated by enhanced vascularization due to DFO treatment (Figure S10, Supporting Information).

The bone's quality can also be evaluated by the trabecular system within the newly formed bone.

The trabecular separation (Tb.Sp.) can be described as the mean distance between the borders of segmented trabeculae [in mm]. A smaller distance signifies denser bone, indicating a better bone structure and quality. Therefore, a decrease in Tb.Sp. is beneficial. The analysis showed that all but 2 study groups (Li 8a, Wei 12)^[^ ^27^, ^33^ ^]^ experienced a significant reduction in Tb.Sp., with a pooled MD of ‐0.21 mm (CI: ‐0.27; ‐0.14). This indicates, that DFO has a positive impact on the structure and density of newly formed bone, enhancing its structural quality (Figure S11, Supporting Information).

Trabecular thickness (Tb.Th.) refers to the mean diameter of the trabeculae of the bone [in mm]. A larger value is preferable because it signifies thicker and potentially stronger trabeculae and bone. The analysis shows a significant pooled MD measuring 0.04 mm (CI: 0.02; 0.07) in favor of DFO (Figure S12, Supporting Information).

Results of vascular and mechanical properties:

To examine the physical stability and properties of the newly formed bone, we looked into different bone quality parameters measured by mechanical strength tests. Eleven studies, included in our review, additionally provided data on various mechanical properties such as yield strength (DonneysA, DonneysD, Felice),^[^ ^18^, ^19^, ^22^ ^]^ stiffness (DonneysA, DonneysD, DonneysY, Liu, (Shen, Stewart)),^[^ ^18^, ^19^, ^20^, ^28^, ^30^, ^31^ ^]^ ultimate load (DonneysA, DonneysD, Felice, Liu)^[^ ^18^, ^19^, ^22^, ^28^ ^]^ and failure load (DonneysY, Felice, Liu, (Stewart))^[^ ^20^, ^22^, ^28^, ^31^ ^]^ (**Table** **3**).

**Table 3:** Characteristics and results of studies investigating vascular and mechanical outcomes.

Study		Outcome measures									
Author	Year	mechanical strength tests				µCT angiography			Time points in weeks	Compared groups: chosen control vs DFO group (study arm)	Inclusion in Meta‐Analysis
		mechanical				vascular					
		Yield	Stiffness	Ultimate load	Failure load	VV	VN	VVF			
Donneys; Ahsan^[^ ^18^ ^]^	2013	X	X	X					6 weeks (POD 40)	XFx vs XFxDFO	Yes
Donneys; Deshpande^[^ ^19^ ^]^	2013	X	X	X					2, 3, 4 weeks	Control vs Experimental	Yes
Donneys; Yang^[^ ^20^ ^]^	2019		X		X				6 weeks (POD 40)	XFx vs iDFO	Yes
Felice^[^ ^22^ ^]^	2013	X		X	X				6 weeks (POD 40)	DO‐XRT vs DO‐XRT‐DFO	Yes
Jia^[^ ^25^ ^]^	2016					X			2 weeks	PLGA vs PLGA+DFO	Yes
Liu^[^ ^28^ ^]^	2022		X	X	X				6 weeks	Control vs Group1	Yes
Matsumoto^[^ ^29^ ^]^	2015							X	1 week (POD 5), 2 weeks (POD 10)	HU5 vs DFOHU5 HU10 vs DFOHU10	Yes
Shen^[^ ^30^ ^]^	2009		X				X	X	4 weeks	Saline vs DFO	No (imprecise n)
Stewart^[^ ^31^ ^]^	2011		X		X	X	X		12 weeks	Saline vs DFO (a) low dose rh‐BMP‐2 vs combined DFO + low dose rh‐BMP‐2 (b)	No (imprecise n)
Wan^[^ ^32^ ^]^	2008						X		2 weeks (POD 17)	Saline vs DFO	Yes
Yan^[^ ^34^ ^]^	2019							X	2 weeks	PCH vs PCD	Yes

**Legend**: VV = vessel volume; VN = vessel number; VVF = vessel volume fraction; DO = distraction osteogenesis; POD = post‐operative day; **Donneys, Ahsan**: XFx = irradiated fracture; **Donneys, Yang**: XFx = irradiated fracture, i = injected; **Felice**: XRT = radiotherapy; **Jia**: PLGA = poly(Lactic‐co‐glycolic acid); **Matsumoto**: HU5/10 = hindlimb unloading for 5 or 10 days; **Stewart**: rh‐BMP2 = recombinant human bone morphogenetic protein‐2; **Yan**: PCL = Polycaprolactone, PCH = aminated PCL, PCD = DFO loaded PCH.

Although several approaches might explain how DFO enhances bone healing, the primary physiological mechanism is thought to be an improvement in the vascularization of the fracture area.^[^ ^40^ ^]^

Therefore, we investigated the reported data of our included studies regarding vascularization parameters measured with µCT angiography such as VV (Jia, (Stewart)),^[^ ^25^, ^31^ ^]^ VN ((Shen, Stewart), Wan),^[^ ^30^, ^31^, ^32^ ^]^ and VVF (Matsumoto, (Shen), Yan)^[^ ^29^, ^30^, ^34^ ^]^ (Table 3).

Due to the limited number of studies reporting data on the outcome categories “mechanical” and “vascular”, we consolidated various parameters (yield, stiffness, ultimate load, and failure load for mechanical outcomes; VV, VN, and VVF for vascular outcomes) for analysis. To facilitate appropriate comparison across these diverse outcome measures, we employed the Standardized Mean Difference (SMD) rather than the Mean Difference (MD). This analysis revealed reduced heterogeneity within the subgroups, although not sufficiently low to justify the use of a fixed effects model. Consequently, we continued with the random effects model for our calculations.

**Figure 5:**
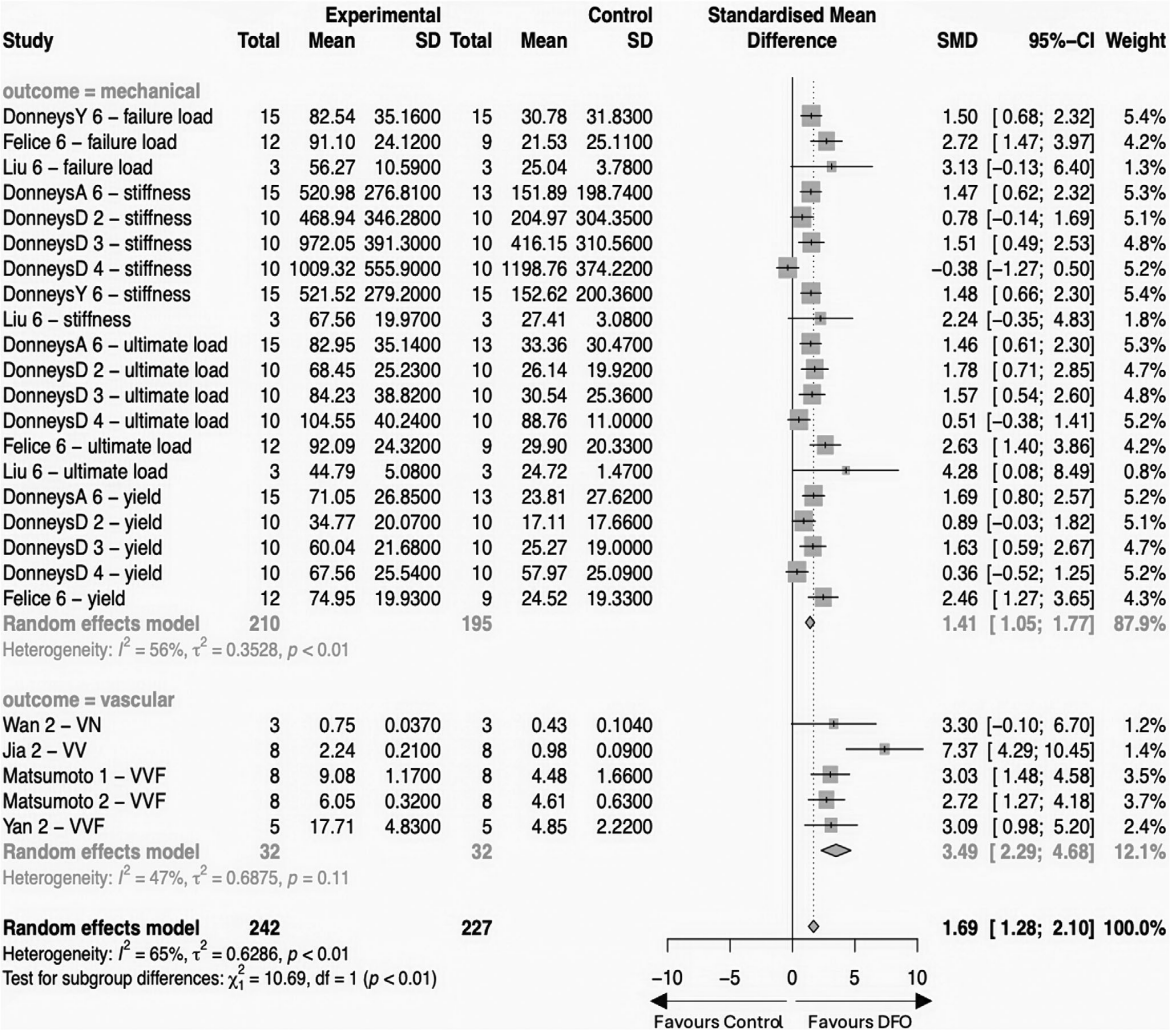
Forest plot displaying results for mechanical and vascular outcomes: The forest plot displays the relative weight of the individual studies and the estimates of treatment effect expressed as standardized mean differences (SMD), and 95% confidence intervals (CI). The diamond at the bottom line indicates the pooled estimate and its 95% CI. References on the left give study names and specific outcome parameters. Study names are designated by the “Lead Author's Name” followed by the “Initial of a Co‐Author (capitalized) if the lead author has contributed multiple studies”, and finally, the “measurement time point after fracture surgery (rounded) in weeks”.

The forest plot (**Figure** **5**) shows that DFO therapy significantly improves the mechanical properties of newly formed bone, as the pooled effect shifts to the right, favoring DFO, and does not cross the line of no effect.

Significant enhancements in vascularization were observed in all but one of the DFO‐treated groups, regardless of the specific vascularization parameter (Figure 5). This supports the theory that DFO's hypoxia‐mimicking mechanism plays a foundational role in promoting vascular improvement and, consequently, bone healing.

Thus, our results consistently demonstrate the superior efficacy of DFO treatments over non‐DFO controls in both promoting vascularization at the fracture site and enhancing the mechanical properties of newly formed bone. These outcomes are in harmony with the findings from other studies that we screened, which specifically examined either vascularity (e.g., Donneys/Farberg 2012, Donneys/Nelson 2015 and 2016, Donneys/Weiss 2013, Farberg 2012, Guzey, Kuchler, Qiu, Zheng)^[^ ^41^, ^42^, ^43^, ^44^, ^45^, ^46^, ^47^, ^48^, ^49^ ^]^ or mechanical properties (e.g., Donneys/Nelson 2016, Guzey).^[^ ^43^, ^46^ ^]^

### 2.8 Sensitivity Analysis

Using the leave‐one‐out method, no study group or study arm was identified as remarkably influential, and removing any single study would not significantly alter the outcomes or heterogeneity associated with the BV/TV data (Figure S13, Supporting Information).

After excluding studies rated as low quality, defined by having less than 3 “low risk” categorical judgments (specifically Cui, Felice, (Geng), Wan, Zeng, Zhao)^[^ ^17^, ^22^, ^23^, ^32^, ^35^, ^36^ ^]^ from the overall study pool, there was a slight alteration in the results. The MD regarding BV/TV shifted from 15.04% (95%‐CI: 11.23; 18.85) to 10.25% (95%‐CI: 8.16; 12.34) (Figure S14, Supporting Information). This suggests that low‐quality studies tended to show higher intervention effects compared to high‐quality ones, with a significant difference of 15.95% between these groups (*p* < 0.01). Furthermore, the low‐quality studies were not as precise in estimating the true effect due to a broader confidence interval of the pooled effect than the high‐quality studies (Figure S14, Supporting Information).

When the effect is calculated excluding small studies with only 3 or fewer subjects (Cui, Fan, Hou, Liu, Wan, Zhao),^[^ ^17^, ^21^, ^24^, ^28^, ^32^, ^36^ ^]^ the pooled effect size's MD marginally decreases by 1,03% without statistical significance (Figure S15, Supporting Information). There was also no significant difference between the small and large study subgroups (*p* = 0.46). To explore the potential influence in greater detail, we subdivided the data into narrower categories based on study size: small (Cui, Fan, Hou, Liu, Wan, Zhao; ≤3),^[^ ^17^, ^21^, ^24^, ^28^, ^32^, ^36^ ^]^ medium (Jia, Lang, Li, Matsumoto, Wei, Yan, Zeng; >3 and ≤8),^[^ ^25^, ^26^, ^27^, ^29^, ^33^, ^34^, ^35^ ^]^ and large (Cheng, DonneysA, DonneysD, DonneysY, Felice; >8).^[^ ^16^, ^18^, ^19^, ^20^, ^22^ ^]^ However, even with this additional stratification, the subgroup differences remained statistically insignificant (*p* = 0.17) (Figure S16, Supporting Information).

Acknowledging that variations in measurement devices could serve as a confounding factor, we conducted a subgroup analysis to evaluate whether differences in µCT device brands and models may impacted the results. Four studies (DonneysA, DonneysD, Felice, Matsumoto)^[^ ^18^, ^19^, ^22^, ^29^ ^]^ were excluded from this analysis due to insufficient reporting on measurement device details. The analysis revealed no significant differences between the device subgroups (*p* = 0.06) (Figure S17, Supporting Information).

Consequently, these sensitivity analyses indicate that the results generated in the meta‐analyses are generally robust and reliable. However, cautious interpretation is warranted.

### 2.9 Publication Bias

**Figure 6:**
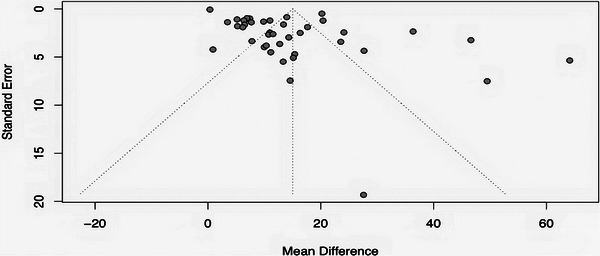
Funnel plot presenting the distribution of the study results in our meta‐analysis, with correlating MD and standard error (SE) of BV/TV data.

The funnel plot is employed to investigate the possibility of publication bias affecting the results (**Figure** **6**). Observations of asymmetry within the funnel plot indicate a possible presence of publication bias or other small‐study effects. Specifically, an absence of studies in regions of high precision (nearer to the apex of the plot) or an uneven distribution of studies across the plot may signal such biases. Our funnel plot reveals a concentration of studies at the apex, indicating a positive aspect of data reliability. However, the noticeable asymmetry within the plot suggests that the results might be influenced by unpublished studies.

The observed asymmetry in the funnel plot may suggest the presence of publication bias or other selection biases. We aimed to mitigate these biases by including all relevant studies that provided the necessary data. However, the asymmetry might also result from other factors, such as the high level of heterogeneity due to our broad inclusion criteria, variations in the methodological quality of the included studies leading to overestimated effect sizes, and the inherent variability in effect sizes caused by diverse study designs and settings. To better assess the impact of publication bias, the “Trim‐and‐Fill” method was employed, which helps to identify the potentially missing studies and to correct the asymmetry in the funnel plot (Figure S18, Supporting Information).

Egger's test, a regression‐based approach to statistically detect funnel plot asymmetry, underscores the potential presence of publication bias in our meta‐analysis. The intercept, calculated at 0.2485 with a standard error (SE) of 0.4975, directly points to asymmetry in the plot. This finding, supported by a t‐statistic of 5.80 and a highly significant p‐value (<0.0001), confirms the statistical significance of the observed asymmetry.

Additionally, our analysis shows a bias estimate of 6.7234, suggesting that smaller studies might be disproportionately influencing the overall effect size, a common sign of publication bias. The considerable residual heterogeneity (τ^2 ^= 47.6362) indicates significant differences in the effects observed across studies, that cannot be attributed to coincidental variability alone.

To further investigate the impact of small studies on asymmetry, we repeated the analyses after excluding studies with ≤3 subjects (Cui, Fan, Hou, Liu, Wan, Zhao).^[^ ^17^, ^21^, ^24^, ^28^, ^32^, ^36^ ^]^

Exclusion of small studies reduced the magnitude of funnel plot asymmetry (bias slope: 6.72 to 5.46) and heterogeneity (τ^2^: 47.64–13.59), suggesting that small studies contribute to both asymmetry and variability in effect sizes (Figures S19 and S20, Supporting Information). However, significant asymmetry persisted in both analyses (*p* < 0.0001), indicating that factors beyond small study effects may also influence the observed bias.

The persistent asymmetry observed after excluding small studies suggests that high standard error (SE) and heterogeneity, likely resulting from differences in study quality or design, are also major contributors. Mathematically, $SE=\frac{SD}{\sqrt{n}}$, which means that studies with broad confidence intervals (reflecting high standard deviation (SD)), or small sample sizes (n), have higher SE. Such studies disproportionately influence funnel plots and Egger's regression, intensifying asymmetry and bias. Specifically, the studies contributing the most substantial negative effect, due to their wide confidence intervals and elevated SE, are DonneysA,^[^ ^18^ ^]^ DonneysY,^[^ ^20^ ^]^ Felice,^[^ ^22^ ^]^ and Wan.^[^ ^32^ ^]^

## 3 Discussion

Our analyses demonstrate significant positive effects of DFO on bone regeneration, independent of the application method, fracture or bone type involved (Figures S2.1,2–S4.1,2, Supporting Information). Notably, the first subgroup analysis focusing on health states indicated that DFO is particularly beneficial for pathological or irradiated fractures (Figure 4; Figure S1, Supporting Information). This subgroup, which includes studies by DonneysA, DonneysY, and Felice,^[^ ^18^, ^20^, ^22^ ^]^ shows that DFO can almost completely restore bone regeneration to levels observed before radiotherapy. That suggests a potential therapeutic value of DFO in counteracting the detrimental adverse effects of radiotherapy on the fracture site, particularly including reduced vessel formation, diminished osteoblast proliferation and the consequent decrease in new bone formation.^[^ ^50^ ^]^

This review also revealed that DFO significantly increases not only the amount and density of the newly formed bone mass, as seen in BV (Figure S8, Supporting Information) and our primary endpoint BV/TV (Figure 3), but also its quality and structure, as investigated in our secondary endpoints BMD, Tb.Sp., and Tb.Th (Figures S10–S12, Supporting Information).

Furthermore, it significantly increases the bone's mechanical strength and the vascularity around the fracture site (Figure 5), which is known as the primary physiological mechanism explaining how DFO enhances bone healing.^[^ ^40^ ^]^

Due to the variability in DFO doses and delivery methods across studies (Table 1), direct comparisons were challenging, as the release times and concentrations differed considerably. Even among studies utilizing the direct injection method, comparisons were impractical because most employed a DFO concentration of 200 µM administered every other day (DonneysA, DonneysD, DonneysY, Liu, Matsumoto, Wan),^[^ ^18^, ^19^, ^20^, ^28^, ^29^, ^32^ ^]^ except for Felice et al.,^[^ ^22^ ^]^ who used a higher concentration of 300 µM. Consequently, our focus shifted toward examining individual studies within our selection that analyzed the effect of the DFO dosage on the outcomes.

A detailed examination of Zeng et al.’s study, which provided data on various DFO dosages using a consistent delivery method, revealed that while all tested concentrations significantly outperformed the DFO‐free control groups, the intermediate‐high dosage (60 mg L^−1^) yielded the most favorable outcomes, surpassing even the highest concentration (120 mg L^−1^).^[^ ^35^ ^]^ This finding underscores the necessity for further research aimed at identifying the optimal DFO dosage and release pattern for the local application way, as it indicates that excessive DFO can be cytotoxic.

In our subgroup analysis comparing the direct injection of DFO versus its delivery via sophisticated vectors, no significant differences in effectiveness were observed between the 2 methods (*p* = 0.32) (Figure S3.1, Supporting Information).

To delve deeper, we examined a specific study by Donneys/Yang et al. that contrasted direct DFO injection (iDFO) with DFO application through a Hyaluronic Acid (HA) scaffold (HA‐DFO) in an irradiated mandible fracture model.^[^ ^20^ ^]^ This study found that both iDFO and HA‐DFO therapies effectively mitigated the radiomorphometric declines observed in the radiation control group (XFx), with no significant difference in BV/TV, BMD, and tissue mineral density (TMD) between the 2 DFO treatment groups. Similarly, both treatments restored the decreased stiffness and failure load seen in the XFx control group, with no significant difference in mechanical outcomes between iDFO and HA‐DFO treatments.

The study highlighted the short half‐life of free DFO (≈0.5 h) and rapid clearance, suggesting that HA could enable a sustained release pattern, retaining 50% of the DFO for up to 10 days. This sustained release is crucial for ensuring that DFO was active before, during, and after the critical angiogenic phase of fracture healing, which typically occurs 7–14 days postinjury or surgery. That also prevents high‐concentration peaks, minimizing concerns regarding DFO‐induced cytotoxicity.

Theoretically, regulating the release pattern and duration to counteract DFO's brief half‐life could enhance healing, yet Donneys/Yang et al's study was unable to confirm such an effect. This area warrants further investigation.^[^ ^20^ ^]^

However, vector delivery systems, compared to direct injections, have the advantage of typically requiring just a single application during fracture surgery. This reduces the need for multiple DFO injections, thereby decreasing pain, risk of infection, and inconvenience, consequently improving patient compliance.

We did not observe remarkable or consistent and explainable differences in the effects of DFO across various time points (Figure S5.1,2, Supporting Information), likely due to the timing of outcome measurements in studies with pronounced effects being coincidental. However, the study by Donneys/Deshpande et al.,^[^ ^19^ ^]^ which specifically explored the effects at different time points (2, 3, and 4 weeks), revealed that DFO's most substantial impact occurs early on, boosting the initial healing process. By 28 days, these effects consolidated, aligning with the levels observed in fully healed bone, thus showing minimal difference from control groups.

DFO is FDA‐approved and commercially available for indications beyond fractures, and the WHO recognizes it as an essential medicine.^[^ ^5^ ^]^ Not only is it cost‐effective, but it also has a minimal adverse event profile, especially when administered locally, a fact supported by the low number of DFO‐related dropouts reported in our included studies.

Despite these advantages and our study's significant and promising results, there's a notable scarcity of research on DFO's use in treating fractures in humans, with no clinical trials and only a handful of case reports available. Most of these reports^[^ ^51^, ^52^, ^53^, ^54^ ^]^ focus on DFO in the context of aluminum‐related bone disease, examining its effect on fracture healing secondary to the primary treatment for aluminum overload.

While all case reports except for Sundaram et al. found enhanced fracture healing with systemic DFO therapy,^[^ ^51^ ^]^ it is crucial to highlight that these outcomes may not directly align with our findings, as our research focused exclusively on local application, potentially achieving higher cumulative DFO concentrations at the fracture site than systemic administration would.

We identified a single human case report, self‐stated as the first clinical report of applying DFO locally to enhance bone formation in a patient following bimaxillary osteotomy and distraction of a previously irradiated maxilla (Momeni et al),^[^ ^55^ ^]^ similar to the work by Donneys/Deshpande et al. and Felice et al.^[^ ^19^, ^22^ ^]^

This report describes the treatment process with local DFO therapy of a 16‐year‐old male during maxillary LeFort I distraction osteogenesis aimed at correcting a negative overjet caused by radiation‐induced maxillary hypoplasia.

The treatment involved administering 0.5 mg of DFO every other day through catheters placed at the osteotomy sites. Due to a dislodged catheter on the right side, DFO was only applied on the left side, allowing the right side to serve as a control. Three months later, CT imaging revealed an increase in bone area and density on the DFO‐treated side, without any adverse events experienced by the patient. These results demonstrate DFO's potential to enhance bone formation in the context of irradiated bone distraction and align with our meta‐analysis findings.

The collective evidence from our review and existing studies highlights local DFO application as a promising therapy for enhancing fracture healing, particularly after radiotherapy to regain vascularization. So, in accordance with most of the screened and investigated studies, we strongly recommend the initiation of clinical trials to further evaluate DFO's efficacy in this context.

In addition to DFO, which enhances angiogenesis by stabilizing HIF‐1α through iron chelation to upregulate VEGF expression, alternative strategies have also shown potential in promoting angiogenesis to improve bone formation and fracture healing. For instance, VEGF‐decorated matrices enable controlled and localized VEGF release, maintaining physiological gradients that couple angiogenesis with osteogenesis while minimizing risks such as excessive osteoclast activity.^[^ ^56^ ^]^ Another approach utilizes scaffolds that release 3‐hydroxybutyrate (3HB), a metabolite that enhances mitochondrial function and amplifies VEGF signaling, thereby creating a metabolically supportive microenvironment for angiogenesis.^[^ ^57^ ^]^ Additionally, lentiviral vector‐based delivery of platelet‐derived growth factor‐BB (PDGF‐BB), a potent chemoattractant and mitogen for mesenchymal and osteogenic cells, stimulates endothelial cell migration and VEGF expression, significantly improving both vascularization and bone tissue formation.^[^ ^58^ ^]^

These diverse strategies highlight the transformative potential of angiogenesis‐focused approaches to accelerate and optimize fracture healing. However, as with DFO, further research is necessary to translate these promising methods into widespread clinical practice.

Our study faces several limitations. The quality of many included studies is constrained by inadequately reported data (Figure 2). We addressed this impact within our sensitivity analysis (Figures S13–S17, Supporting Information). Although the ARRIVE guidelines (Animal Research: Reporting of In Vivo Experiments),^[^ ^59^, ^60^ ^]^ first introduced in 2010, were designed to improve the quality, transparency, and reproducibility of animal research, adherence has been inconsistent. The study by Wan et al. (2008),^[^ ^32^ ^]^ categorized as “low quality” due to poor reporting, predates these guidelines, which likely contributed to its shortcomings. Similarly, Felice et al. (2013)^[^ ^22^ ^]^ conducted their study when the guidelines were still relatively new, potentially explaining some reporting gaps. Later studies, however, should have had the opportunity to follow these recommendations more rigorously.

The heterogeneity in study designs, including variations in DFO dosage, administration frequency, route, timing, animal species, and disease models, was a deliberate choice to avoid overly restrictive inclusion criteria. This approach allowed for a broader dataset and for evaluating DFO's effects regarding secondary endpoints but also contributed to the high heterogeneity observed in our meta‐analysis, which we attempted to mitigate through random effects modeling and subgroup analyses.

Some subgroup analyses may have been disproportionately influenced by outliers, particularly in smaller subgroups where such studies exerted a greater impact on the pooled results. Efforts to minimize this effect included using the random effects model and conducting analyses that excluded disease model studies tending to show more extreme results (Figures S2.2–S7.2, Supporting Information). The wider CIs in disease model studies suggest less precision in estimating true effects.

The heterogeneity identified in our analyses may have contributed to asymmetry in the funnel plot (Figure 6) and significant findings in Egger's regression. These tests primarily suggest potential publication bias within our meta‐analysis, which is further supported by the Trim‐and‐fill plot displaying the likely omission of studies with negative results (Figure S18, Supporting Information). Moreover, many studies included small sample sizes, a limitation we have attempted to address through sensitivity analysis (Figures S15 and S16, Supporting Information).

Potentially influential factors, such as the sex and age of the subjects, showed no significant subgroup differences, indicating that DFO is equally effective across sexes and age groups, as demonstrated by our subgroup analyses (Figures S6.1,2 and S7.1,2, Supporting Information). Additionally, while variations in measurement devices and dosing regimens across studies were considered potential confounders, our sensitivity analysis confirmed that different µCT devices did not significantly impact the outcomes (Figure S17, Supporting Information). However, differences in dosing remain a potential source of bias, though a more detailed investigation was not feasible. These varying study parameters and settings contributed to the high heterogeneity observed in our meta‐analysis. We statistically addressed this by employing the random‐effects model and conducting extensive subgroup analyses to account for such variability.

## 4 Conclusion 

In conclusion, the findings of this systematic review and meta‐analysis underscore the great potential of local DFO therapy as a promising medical compound for enhancing fracture healing. Given its significantly positive outcomes, excellent safety profile characterized by minimal adverse effects due to local application, cost‐effectiveness, and existing FDA approval, alongside its recognition by the WHO as an essential medicine, we advocate for the prompt initiation of clinical trials aimed at rapid clinical translation. This therapy holds promise to benefit all patients with various types of fractures, particularly those at high risk of bone healing complications, especially after radiotherapy. Further research is essential to optimize dosing and delivery systems.

## 5 Materials and Methods

This systematic review and meta‐analysis was performed according to the “Preferred Reporting Items for Systematic Reviews and Meta‐analysis” (PRISMA) guidelines^[^ ^61^ ^]^ and was prospectively registered on the “International Prospective Register of Systematic Reviews” (PROSPERO). The associated protocol can be found under the registration number CRD42024492533. Formal ethical approval was not required since this research is not a human or animal experiment and only includes already published data.

The PICO (Population, Intervention, Comparison, and Outcomes) model was used to define the inclusion criteria. We evaluated the internal quality and validity of the included studies using the Risk of Bias (RoB) tool by the “Systematic Review Center of Laboratory Animal Experimentation” (SYRCLE).^[^ ^62^ ^]^

All statistical procedures for the meta‐analysis were performed utilizing the “meta” package within R and RStudio. We generated all plots and figures through R and RStudio software and employed Microsoft Excel for data extraction and the construction of tables.

### 5.1 Systematic Search Strategy

We systematically searched the electronic databases Medline (PubMed) and Embase via OvidSP as well as the database Web of Science (WOS), along with a separate free MeSH term and Emtree search, a review of grey literature, and we looked for additional eligible studies in the reference lists of our included studies and other topic‐related articles.

To ensure comprehensiveness, the registers “Animal Study Registry”, “PreclinicalTrials.EU” and “Open Science Framework” were explored for registered but unpublished preclinical animal studies on DFO's role in fracture healing. We imposed no restrictions on publication dates. The initial search was conducted in October 2023 and re‐ran in January 2024 to retrieve the latest studies using the following search strategy (**Table** **4**):

**Table 4:** Search strategy in MEDLINE and EMBASE using OvidSP and in WOS (03.01.2024).

		Results		
Searches		MEDLINE	EMBASE	WOS
1	(Deferoxamine or DFO$ or Desferoxamine or Desferrioxamine or Desferal).ti, ab.	10046	13136	14529
2	(((bon$ or fractur$ or skelet$ or callus) and (defect$ or seperat$ or heal$ or fusion$ or regenerat$ or remodel$ or repa$ or reconstruct$ or mass or $union or vasculariz$ or format$ or gap)) or distraction osteogenesis).ti, ab.	619822	820142	1092819
3	1 and 2	313	487	447
4	(rat or rats or mouse or mice or murine).mp.	3754526	4706134	5860447
5	3 and 4	116	193	165
6	limit 5 to (english or german)	113	189	161
7	remove duplicates from 6	113	185	161
		Deduplicated total: **209**		

“$” = substitute for any string of zero or more characters; “ti” = title; “ab” = abstract; “mp” = multi‐purpose (searching in various fields). Search syntax, including truncations, had to be adjusted to the WOS application (Text S1) and for searches in other electronic databases.

### 5.2 Study Selection

In alignment with the PRISMA Protocol, 2 reviewers independently completed the search and screened the identified literature for relevance based on the predetermined inclusion and exclusion criteria using EndNote management software 20.5.

For title and abstract screening, the main criteria were liberally applied to ensure the inclusion of all relevant studies and being sorted in the following hierarchical order: 1) Primary study; 2) fracture model; 3) in vivo preclinical animal study using rats or mice models; 4) DFO application; 5) relevant outcome measures (BV/TV or BV); 6) measurements done with µCT.

Full texts of the remaining studies were screened for eligibility by 2 independent reviewers with a stricter application of the inclusion and exclusion criteria.

Following this search and screening strategy, all studies retrieved were thoroughly evaluated in regard to the predetermined eligibility criteria (PROSPERO Protocol: CRD42024492533).

Discrepancies were resolved through discussion and consensus between the observers. If no agreement could be reached, the supervisors checked the results and arbitrated.

### 5.3 Eligibility Criteria

#### 5.3.1 Types of Studies

We included all preclinical in vivo animal intervention studies published in English or German up to the 3rd of January 2024 that have an original full‐text publication and underwent the peer‐review process.

Hence, non‐original full‐text articles, reviews, systematic reviews, meta‐analyses, case reports, expert opinions, letters to the editor, conference abstracts, and preprints were excluded from this review.

#### 5.3.2 Types of Subjects

The review was limited to studies employing rat or mouse models, with no restrictions on strain, sex, or age. Other rodents and species were excluded to reduce heterogeneity.

#### 5.3.3 Types of Intervention

Our criteria embraced all studies investigating fractures with fracture gaps, regardless of whether surgical fixation was employed, including various experimental models such as induced animal disease models and distraction osteogenesis. The mode of local DFO administration, whether through direct injection or via sophisticated vector systems being loaded with DFO (e.g., scaffolds, hydrogels, nanoparticles), was considered without constraints on timing, dosage, or frequency.

Exclusions were applied to studies focused solely on bone formation around implants or prostheses, dental and alveolar fractures, and those lacking adequate controls. This ensured the exclusivity of DFO's effects in the evaluation, ruling out studies where auxiliary agents were not comparably used across groups.

### 5.4 Outcome Measures

In our systematic review and meta‐analysis, we primarily concentrated on bone parameters measured by µCT, as it is currently seen as the gold‐standard method for analyzing the 3D morphology and microarchitecture of bones in small animals such as rats and mice.^[^ ^63^, ^64^, ^65^ ^]^

Due to their clinical relevance and widespread research application, we focused on bone volume fraction (BV/TV [in %]) and bone volume (BV [in mm^3^]) as primary endpoints in our systematic review for evaluating and quantifying new bone formation around the fracture site. For our meta‐analysis, we only included studies that provide essential data on BV/TV. In cases where studies only report BV and tissue volume (TV [in mm^3^]), we included the study in our meta‐analysis and used these given values to calculate the missing BV/TV data.

Secondary outcome measures included µCT‐assessed parameters such as bone mineral density (BMD [in mg/cc]), trabecular separation or spacing (Tb.Sp. [in mm]), and trabecular thickness (Tb.Th. [in mm]), which provided insights into bone quality.

Additionally, we evaluated parameters indicative of vascularization at the fracture site, including vessel volume (VV [in mm^3^]), vessel number (VN [in number of vessels per defect area]), and vessel volume fraction (VVF [in %]), as assessed by µCT angiography. We also examined the mechanical integrity of newly formed bone, focusing on yield strength [in N], stiffness [in N/mm], and both ultimate and failure load [in N], as determined through mechanical strength tests.

Furthermore, we concentrated on potential influences from factors like application method, bone type, health state (healthy or diseased models), type of the surgically induced fracture defect model, and timing of DFO application, particularly in the subgroup analyses using the data of our primary bone parameter BV/TV.

We excluded data derived solely from histological evaluations of the mentioned bone and vessel formation parameters to maintain consistency with our µCT‐based analytical framework.

### 5.5 Data Extraction

Qualitative and quantitative data were extracted by the main reviewer from the full text of the included literature as predetermined in our protocol. The extracted data was meticulously reviewed and cross‐checked by a second reviewer, with discussion around discrepancies to reach a consensus.

Quantitative outcome values and figures were extracted exactly as mentioned in the original study, including the mean ± standard deviation (SD). If the primary data was not presented in a direct form in the text or tables and hence was only illustrated as graphs or diagrams, we used the online tool “Webplotdigitizer” for extraction.^[^ ^66^ ^]^ In instances of absent or ambiguous data reporting (Geng, Shen, Stewart), authors were contacted via E‐mail to ask for the required data, allowing a 12‐week period for response. Lack of reply resulted in exclusion from the meta‐analysis, but not necessarily from the systematic review.

Measurement time points given in post‐operative days (POD) were rounded to whole weeks for better statistical calculations and comparisons.

For studies that did not present the results data in the required format, we calculated the missing information (mean ± SD) based on the given data. In instances where SD values were not reported, such as in the study by Lang et al., we calculated SD using the individual data points provided within the study. For data presented as the standard error of the mean (SEM), as in the study by Zeng et al., we converted SEM to SD utilizing the formula: $SD=SEM\;\times \;\sqrt{n}$, where n represents the number of samples.

If only BV and TV were given with the associated SDs, we used the following formulations to calculate our primary outcome BV/TV with their respective SD:

(1) $$\begin{array}{cc} Regenerated\;Bone\;Rate & =Bone\;Volume\;Fraction\;\;\left(BV/TV\right)\\ & =\frac{regenerated\;Bone\;Volume\;\left(BV\right)}{Bone\;Defect\;Volume\;\left(TV\right)}\;\times 100\\ & =BV/TV\;\left[in\;\%\right] \end{array}$$

(2) $$SD_{BV/TV}=BV/TV\times \sqrt{{\left(\frac{SD_{BV}}{BV}\right)}^{2}+{\left(\frac{SD_{TV}}{TV}\right)}^{2}}$$

Missing BV and TV values and their respective SDs were calculated using the modified versions of these formulas.

Specifically, in our dataset, we calculated BV/TV and its SD using the provided BV and TV values for the study by Donneys/Ahsan et al.^[^ ^18^ ^]^ and converted SEM to SD for the study by Zeng et al.^[^ ^35^ ^]^

To address variations in the units of outcome measures across different studies, we mathematically adjusted these units within our dataset. This was accomplished by converting original data to the units most commonly used across the studies, allowing us to employ mean difference (MD) rather than standardized mean difference (SMD) for our meta‐analysis.

Qualitative information was systematically extracted and presented in the characteristics and results tables, in line with our predefined protocol.

### 5.6 Quality Assessment and Risk of Bias/Critical Appraisal

The internal validity and methodological quality of the 21 included animal studies were independently analyzed according to the SYRCLE's risk of bias tool (SYRCLE RoB) for animal studies^[^ ^62^ ^]^ by 2 investigators who were not blinded to the article authors, journal, or institution. Using this tool, we assessed 6 domains of bias: “selection bias,” “performance bias,” “detection bias,” “reporting bias,” “attrition bias,” and “other sources of bias.” To ensure a standardized and precise evaluation, we employed the “signaling questions” framework outlined by Hooijmans et al.^[^ ^62^ ^]^ Bias was categorized as “low risk,” “high risk,” or “unclear risk.” A “low risk” judgement was assigned when the study included comprehensive information about the specific methodological approach and followed the highest standards of practice for the relevant procedures. A “high risk” classification was applied if the study offered sufficient methodological descriptions but deviated from the gold standard. An “unclear risk” rating was used if the study lacked enough information to properly evaluate the specific risk of bias item.

A study was deemed to be of low quality if it received less than 3 “low risk” ratings across the assessed categories, indicating a significant deficiency in the reported study design details. Any disagreements between the 2 reviewers were resolved via discussion and consensus. The software R and RStudio were utilized to generate the quality assessment figures (Figure 2).

### 5.7 Data Synthesis and Statistical Analysis

For the meta‐analysis, the MDs were calculated with the associated 95% confidence intervals (95%‐CI) to estimate overall bone regeneration and quality for the respective continuous outcome parameters BV/TV (primary outcome) as well as BV, TV, BMD, Tb.Sp., and Tb.Th. (secondary outcomes).

The forest plots were generated to display the study results with pooled estimates, providing a visual summary of the data containing the included studies’ effect sizes, means, and confidence intervals. Furthermore, this visualization helped to assess the overall trend and variability in the results, aiding in interpreting our meta‐analysis findings.

Subjective measurements and evaluations of bone regeneration, like bone union, were excluded due to their inherent subjectivity and potential for inaccuracies.

*p*‐values < 0.05 were considered statistically significant.

### 5.8 Publication Bias and Heterogeneity

We employed the inverse variance method within a random effects model to weight the studies in our meta‐analysis, ensuring a nuanced overall effect estimate reflective of the degree of heterogeneity.

The Q test and inconsistency (I^2^) test were used to assess the degree of heterogeneity between the trials. I^2^ test values <25%, between 25%–50%, and >50% were classified as indicating low, moderate, and high heterogeneity, respectively,^[^ ^67^ ^]^ complemented by the Cochranes Q test to ascertain the significance of observed heterogeneity.

For moderate to high heterogeneity within the pooled outcomes, we applied the random effects model, whereas the fixed effect model was considered in cases of low heterogeneity, aligning with established meta‐analytical practices.

To visually inspect for potential publication bias and present the distribution of the study results in our meta‐analysis, we constructed a funnel plot correlating MD with standard error (SE). Egger regression was conducted to statistically evaluate funnel plot asymmetry. The “Trim‐and‐Fill” method was exerted to identify and adjust for potentially missing studies.

### 5.9 Subgroup and Sensitivity Analysis

In our subgroup analyses, we meticulously evaluated the impact of qualitative scientific and clinically relevant criteria on our primary outcome, BV/TV.

As predetermined in our protocol, we grouped the studies by the categories of health state (healthy vs disease model), bone types (long vs cranial), type of fracture defect model (drill hole vs fracture gap), DFO application route (direct injection vs vector system) and the time point of measurement (1 to 12 weeks).

For our secondary bone parameters (BV, TV, BMD, Tb.Sp., Tb.Th.), subgroup differentiation was not feasible due to the limited number of studies reporting on these outcomes. However, to discern the potential impact of bone health on these outcomes, we created subgroups within the analyses for diseased and healthy bone models.

For the subgroup analyses regarding our primary endpoint BV/TV, we did not impose a minimum number of studies required for eligibility within a single subgroup, although we recognized the limited statistical power of very small subgroups in our interpretation of the results. Additionally, these subgroups had minimal impact on the overall outcome due to the random effects model, which assigned them relatively low weight.

Conducting a meta‐regression was not practicable due to inconsistent data availability.

In our sensitivity analyses, we employed a systematic approach to assess the robustness of our meta‐analysis results. Initially, we applied the leave‐one‐out method, systematically removing each study or study arm in turn, to determine the individual impact on the primary outcome. Subsequently, we conducted an analysis excluding studies identified as having a high risk of bias to observe whether any low‐quality study influences the overall outcome. Finally, we also performed an analysis excluding studies with small sample sizes (*n* < 4), addressing concerns about the reliability and generalizability of their findings. The influence of these variations on the meta‐analysis results was examined and discussed.

## Conflict of Interest

The authors declare no conflict of interest.

## Author Contributions

D.M. screened and reviewed the literature, performed the quality assessment, extracted and analyzed the data, conducted the statistical analyses, designed the figures and tables, and wrote the manuscript. M.K. was the second reviewer, performing screening and quality assessment and reviewing the correctness of the extracted data. P.H. and T.G. initiated this systematic review and meta‐analysis, provided key ideas for this research, acted as supervisors to support with expertise, and critically revised and adapted the manuscript. J.K. meticulously revised and verified the statistics. F.B. and C.P. thoroughly checked and edited the manuscript. All authors contributed to the article, reviewed the manuscript and approved the submitted version.

## Supporting information



Supporting Information

Supplementary Table 1

## Data Availability

The raw data supporting the conclusion of this article will be made available by the authors upon request.

## References

[1] C. Bergh , D. Wennergren , M. Möller , H. Brisby , PLoS One 2020, 15, e0244291. 33347485 10.1371/journal.pone.0244291 PMC7751975

[2] R. Zura , Z. Xiong , T. Einhorn , J. T. Watson , R. F. Ostrum , M. J. Prayson , G. J. Della Rocca , S. Mehta , T. McKinley , Z. Wang , R. G. Steen , JAMA Surg. 2016, 151, e162775. 27603155 10.1001/jamasurg.2016.2775

[3] A. Bigham‐Sadegh , A. Oryan , Int. Wound J. 2015, 12, 238. 24618334 10.1111/iwj.12231 PMC7950494

[4] X. Jing , T. Du , X. Yang , W. Zhang , G. Wang , X. Liu , T. Li , Z. Jiang , J. Cell. Physiol. 2020, 235, 9864. 32437020 10.1002/jcp.29799

[5] J. B. Parker , M. F. Griffin , M. A. Downer , D. Akras , C. E. Berry , A. C. Cotterell , G. C. Gurtner , M. T. Longaker , D. C. Wan , Front. Med. 2023, 10, 1015711. 10.3389/fmed.2023.1015711 PMC9975168 36873870

[6] J. Filipowska , K. A. Tomaszewski , Ł. Niedźwiedzki , J. A. Walocha , T. Niedźwiedzki , Angiogenesis 2017, 20, 291. 28194536 10.1007/s10456-017-9541-1 PMC5511612

[7] H. ElHawary , A. Baradaran , J. Abi‐Rafeh , J. Vorstenbosch , L. Xu , J. I. Efanov , Semin. Plast. Surg. 2021, 35, 198. 34526868 10.1055/s-0041-1732334 PMC8432998

[8] J. R. Sheen , A. Mabrouk , V. V. Garla , Fracture Healing Overview, StatPearls Publishing, Treasure Island (FL) 2023. 31869142

[9] R. Marsell , T. A. Einhorn , Injury 2011, 42, 551. 21489527 10.1016/j.injury.2011.03.031 PMC3105171

[10] J. Velasquez , A. A. Wray , Deferoxamine, StatPearls Publishing, Treasure Island (FL) 2023.

[11] K. Sridharan , G. Sivaramakrishnan , Expert Rev. Clin. Pharmacol. 2018, 11, 641. 29727586 10.1080/17512433.2018.1473760

[12] C. Bollig , L. K. Schell , G. Rücker , R. Allert , E. Motschall , C. M. Niemeyer , D. Bassler , J. J. Meerpohl , Cochrane Database Syst. Rev. 2017, 8. 10.1002/14651858.CD007476.pub3 PMC6483623 28809446

[13] T. Qadah , J. Int. Med. Res. 2022, 50, 3000605221143290. 36562113 10.1177/03000605221143290 PMC9793042

[14] T. Sun , Y. Y. Zhao , Q. X. Xiao , M. Wu , M. Y. Luo , Clin. Neurol. Neurosurg. 2023, 227, 107634. 36857886 10.1016/j.clineuro.2023.107634

[15] H. J. Cui , H. Y. He , A. L. Yang , H. J. Zhou , C. Wang , J. K. Luo , Y. Lin , T. Tang , PLoS One 2015, 10, e0127256. 26000830 10.1371/journal.pone.0127256 PMC4441464

[16] W. Cheng , Z. Ding , X. Zheng , Q. Lu , X. Kong , X. Zhou , G. Lu , D. L. Kaplan , Biomater. Sci. 2020, 8, 2537. 32215404 10.1039/d0bm00104j PMC7204512

[17] J. Cui , X. Yu , B. Yu , X. Yang , Z. Fu , J. Wan , M. Zhu , X. Wang , K. Lin , Adv. Healthcare Mater. 2022, 11. 10.1002/adhm.202200571 35668705

[18] A. Donneys , S. Ahsan , J. E. Perosky , S. S. Deshpande , C. N. Tchanque‐Fossuo , B. Levi , K. M. Kozloff , S. R. Buchman , Plast. Reconstr. Surg. 2013, 131, 711e. 10.1097/PRS.0b013e3182865c57 PMC3641519 23629110

[19] A. Donneys , S. S. Deshpande , C. N. Tchanque‐Fossuo , K. L. Johnson , J. T. Blough , J. E. Perosky , K. M. Kozloff , P. A. Felice , N. S. Nelson , A. S. Farberg , B. Levi , S. R. Buchman , Bone 2013, 55, 384. 23598047 10.1016/j.bone.2013.04.005 PMC4162399

[20] A. Donneys , Q. Yang , M. L. Forrest , N. S. Nelson , T. Zhang , R. Ettinger , K. Ranganathan , A. Snider , S. S. Deshpande , M. S. Cohen , S. R. Buchman , Regener. Med. 2019, 4. 10.1038/s41536-019-0072-9 PMC6529413 31123600

[21] Z. Fan , H. Liu , S. Shi , Z. Ding , Z. Zhang , Q. Lu , D. L. Kaplan , Mater. Today. Bio. 2022, 15, 100283. 10.1016/j.mtbio.2022.100283 PMC9130114 35634170

[22] P. A. Felice , S. Ahsan , A. Donneys , S. S. Deshpande , N. S. Nelson , S. R. Buchman , Plast. Reconstr. Surg. 2013, 132, 542e. 10.1097/PRS.0b013e31829fe548 PMC3787312 24076701

[23] M. Geng , Q. Zhang , J. Gu , J. Yang , H. Du , Y. Jia , X. Zhou , C. He , Biomater. Sci. 2021, 9, 2631. 33595010 10.1039/d0bm02058c

[24] J. Hou , Z. Ding , X. Zheng , Y. Shen , Q. Lu , D. L. Kaplan , Adv. Healthcare Mater. 2023, 12, e2203050. 10.1002/adhm.202203050 36841910

[25] P. Jia , H. Chen , H. Kang , J. Qi , P. Zhao , M. Jiang , L. Guo , Q. Zhou , N. D. Qian , H. B. Zhou , Y. J. Xu , Y. Fan , L. F. Deng , J. Biomed. Mater. Res.– Part A 2016, 104, 2515. 10.1002/jbm.a.35793 27227768

[26] A. Lang , J. Stefanowski , M. Pfeiffenberger , A. Wolter , A. Damerau , S. Hemmati‐Sadeghi , R. Haag , A. E. Hauser , M. Lohning , G. N. Duda , P. Hoff , K. Schmidt‐Bleek , T. Gaber , F. Buttgereit , Bone 2022, 154, 116247. 34743042 10.1016/j.bone.2021.116247

[27] R. Li , J. Zhang , J. Shi , J. Yue , Y. Cui , Q. Ye , G. Wu , Z. Zhang , Y. Guo , D. Fu , J. Mater. Chem. B. 2023, 11, 2946. 36916173 10.1039/d2tb02725a

[28] Y. Liu , J. Liu , F. Cai , K. Liu , X. Zhang , A. Yusufu , Front. Physiol. 2022, 13. 10.3389/fphys.2022.804469 PMC8905603 35283791

[29] T. Matsumoto , S. Sato , Physiol. Rep. 2015, 3, e12335. 25780087 10.14814/phy2.12335 PMC4393168

[30] X. Shen , C. Wan , G. Ramaswamy , M. Mavalli , Y. Wang , C. L. Duvall , F. D. Lian , R. E. Guldberg , A. Eberhart , T. L. Clemens , S. R. Gilbert , J. Orthopaed. Res. 2009, 27, 1298. 10.1002/jor.20886 PMC3767389 19338032

[31] R. Stewart , J. Goldstein , A. Eberhardt , G. T. M. Gabriel Chu , S. Gilbert , J. Orthopaed. Trauma 2011, 25, 472. 10.1097/BOT.0b013e31822588d8 PMC3748583 21738061

[32] C. Wan , S. R. Gilbert , Y. Wang , X. Cao , X. Shen , G. Ramaswamy , K. A. Jacobsen , Z. S. Alaql , A. W. Eberhardt , L. C. Gerstenfeld , T. A. Einhorn , L. Deng , T. L. Clemens , Proc. Natl. Acad. Sci. USA 2008, 105, 686. 18184809 10.1073/pnas.0708474105 PMC2206597

[33] S. Wei , R. G. Zhang , Z. Y. Wang , J. Biomater. Appl. 2022, 37, 838. 35984333 10.1177/08853282221121882

[34] Y. Yan , H. Chen , H. Zhang , C. Guo , K. Yang , K. Chen , R. Cheng , N. Qian , N. Sandler , Y. S. Zhang , H. Shen , J. Qi , W. Cui , L. Deng , Biomaterials 2019, 190–191, 97. 10.1016/j.biomaterials.2018.10.033 30415019

[35] Y. Zeng , C. Huang , D. Duan , A. Lou , Y. Guo , T. Xiao , J. Wei , S. Liu , Z. Wang , Q. Yang , L. Zhou , Z. Wu , L. Wang , Acta. Biomater. 2022, 153, 108. 36115651 10.1016/j.actbio.2022.09.018

[36] Y. Zhao , H. Chen , K. Ran , Y. Zhang , H. Pan , J. Shangguan , M. Tong , J. Yang , Q. Yao , H. Xu , Biomater. Adv. 2023, 144, 213202. 36434928 10.1016/j.bioadv.2022.213202

[37] D. I. Lewis , Emerging Topics Life Sci. 2019, 3, 675. 10.1042/ETLS20190061 32915219

[38] H. Elkhateeb , E. S. Mohammed , D. Mohamedien , Y. A. Ahmed , S. A. Soliman , SVU‐Int. J. Vet. Sci. 2023, 6, 44.

[39] R. L. Jilka , J. Gerontol. A Biol. Sci. Med. Sci. 2013, 68, 1209. 23689830 10.1093/gerona/glt046 PMC3779631

[40] J. Zhang , H. Zhao , G. Yao , P. Qiao , L. Li , S. Wu , Biomed. Pharmacother. 2021, 137, 111380. 33601146 10.1016/j.biopha.2021.111380

[41] A. Donneys , A. S. Farberg , C. N. Tchanque‐Fossuo , S. S. Deshpande , S. R. Buchman , Plast. Reconstr. Surg. 2012, 129, 850. 22456357 10.1097/PRS.0b013e31824422f2 PMC4535714

[42] A. Donneys , N. S. Nelson , E. E. Page , S. S. Deshpande , P. A. Felice , C. N. Tchanque‐Fossuo , J. P. Spiegel , S. R. Buchman , Head Neck 2015, 37, 1261. 24801669 10.1002/hed.23744 PMC4788100

[43] A. Donneys , N. S. Nelson , J. E. Perosky , Y. Polyatskaya , J. J. Rodriguez , C. Figueredo , C. A. Vasseli , H. C. Ratliff , S. S. Deshpande , K. M. Kozloff , S. R. Buchman , Bone 2016, 84, 245. 26723578 10.1016/j.bone.2015.12.051 PMC4776634

[44] A. Donneys , D. M. Weiss , S. S. Deshpande , S. Ahsan , C. N. Tchanque‐Fossuo , D. Sarhaddi , B. Levi , S. A. Goldstein , S. R. Buchman , Bone 2013, 52, 318. 23085084 10.1016/j.bone.2012.10.014 PMC3513581

[45] A. S. Farberg , X. L. Jing , L. A. Monson , A. Donneys , C. N. Tchanque‐Fossuo , S. S. Deshpande , S. R. Buchman , Bone 2012, 50, 1184. 22314387 10.1016/j.bone.2012.01.019 PMC3322244

[46] S. Guzey , A. Aykan , S. Ozturk , H. Avsever , Y. Karslioglu , A. Ertan , Ann. Plast. Surg. 2016, 77, 560. 26808734 10.1097/SAP.0000000000000679

[47] U. Kuchler , C. Keibl , A. Fugl , U. Y. Schwarze , S. Tangl , H. Agis , R. Gruber , Clin. Oral Implants Res. 2015, 26, 485. 25196581 10.1111/clr.12474

[48] M. Qiu , Z. Cai , C. Li , K. Yang , N. Tulufu , B. Chen , L. Cheng , C. Zhuang , Z. Liu , J. Qi , W. Cui , L. Deng , Adv. Sci. 2023, 10, e2207089. 10.1002/advs.202207089 PMC10238192 36999832

[49] X. Zheng , X. Zhang , Y. Wang , Y. Liu , Y. Pan , Y. Li , M. Ji , X. Zhao , S. Huang , Q. Yao , Bioact. Mater. 2021, 6, 3485. 33817422 10.1016/j.bioactmat.2021.03.011 PMC7988349

[50] N. Green , S. French , G. Rodriquez , M. Hays , A. Fingerhut , Radiology 1969, 93, 635. 5822740 10.1148/93.3.635

[51] M. Sundaram , D. Dessner , S. Ballal , Skeletal Radiol. 1991, 20, 91. 2020868 10.1007/BF00193817

[52] P. Garrett , M. McWade , J. O'Callaghan , Clin. Radiol. 1986, 37, 63. 3956096 10.1016/s0009-9260(86)80174-x

[53] K. R. Phelps , T. A. Einhorn , V. J. Vigorita , A. P. Lundin , E. A. Friedman , ASAIO Trans. 1986, 32, 198. 3778712

[54] J. I. Sebes , M. L. Pinstein , J. D. Massie , R. L. Scott , G. M. Palmieri , J. W. Williams , S. R. Acchiardo , AJR Am J. Roentgenol. 1984, 142, 424. 6229982 10.2214/ajr.142.2.424

[55] A. Momeni , S. Rapp , A. Donneys , S. R. Buchman , D. C. Wan , J. Craniofacial Surg. 2016, 27, 880. 10.1097/SCS.0000000000002633 PMC4902756 27171947

[56] M. G. Burger , A. Grosso , P. S. Briquez , G. M. E. Born , A. Lunger , F. Schrenk , A. Todorov , V. Sacchi , J. A. Hubbell , D. J. Schaefer , A. Banfi , N. Di Maggio , Acta Biomater. 2022, 149, 111. 35835287 10.1016/j.actbio.2022.07.014

[57] J. Li , X. Zhang , Z. X. Peng , J. H. Chen , J. H. Liang , L. Q. Ke , D. Huang , W. X. Cheng , S. Lin , G. Li , R. Hou , W. Z. Zhong , Z. J. Lin , L. Qin , G. Q. Chen , P. Zhang , Trends Biotechnol. 2024, 1745. 39237385 10.1016/j.tibtech.2024.08.002

[58] J. Li , Q. Xu , B. Teng , C. Yu , J. Li , L. Song , Y. X. Lai , J. Zhang , W. Zheng , P. G. Ren , Acta Biomater. 2016, 42, 389. 27326916 10.1016/j.actbio.2016.06.024

[59] N. P. Du Sert , A. Ahluwalia , S. Alam , M. T. Avey , M. Baker , W. J. Browne , A. Clark , I. C. Cuthill , U. Dirnagl , M. Emerson , PLoS Biol. 2020, 18, e3000411. 32663221 10.1371/journal.pbio.3000411 PMC7360025

[60] J. C. McGrath , G. Drummond , E. McLachlan , C. Kilkenny , C. Wainwright , Br. J. Pharmacol. 2010, 160, 1573. 20649560 10.1111/j.1476-5381.2010.00873.x PMC2936829

[61] M. J. Page , J. E. McKenzie , P. M. Bossuyt , I. Boutron , T. C. Hoffmann , C. D. Mulrow , L. Shamseer , J. M. Tetzlaff , E. A. Akl , S. E. Brennan , Int. J. Surg. 2021, 88, 105906. 33789826 10.1016/j.ijsu.2021.105906

[62] C. R. Hooijmans , M. M. Rovers , R. B. M. de Vries , M. Leenaars , M. Ritskes‐Hoitinga , M. W. Langendam , BMC Med. Res. Methodol. 2014, 14, 43. 24667063 10.1186/1471-2288-14-43 PMC4230647

[63] M. L. Bouxsein , S. K. Boyd , B. A. Christiansen , R. E. Guldberg , K. J. Jepsen , R. Müller , J. Bone Miner. Res. 2010, 25, 1468. 20533309 10.1002/jbmr.141

[64] Y. Kim , M. D. Brodt , S. Y. Tang , M. J. Silva , Methods Mol. Biol. 2021, 2230, 169. 33197015 10.1007/978-1-0716-1028-2_11 PMC8409170

[65] E. Donnelly , Clin. Orthop. Relat. Res. 2011, 469, 2128. 21116752 10.1007/s11999-010-1702-0 PMC3126959

[66] A. W. P. D. Rohatgi , *Available from*: https://automeris.io/WebPlotDigitizer August, 2021.

[67] J. P. T. Higgins , S. G. Thompson , J. J. Deeks , D. G. Altman , BMJ 2003, 327, 557. 12958120 10.1136/bmj.327.7414.557 PMC192859

