# Supplementary material for: Fracture Fusion on Fast‐Forward: Locally Administered Deferoxamine Significantly Enhances Fracture Healing in Animal Models: A Systematic Review and Meta‐Analysis
Source: Adv Sci (Weinh). 2025 Jan 22;12(8):2413290. doi: 10.1002/advs.202413290 (PMC11848589; doi:10.1002/advs.202413290)
Supplement: Supplementary file 1 — Supporting Information [file ADVS-12-2413290-s001.pdf]

ADVANCED SCIENCE
Open Access

# Supporting Information

for *Adv. Sci.*, DOI 10.1002/advs.202413290

Fracture Fusion on Fast-Forward: Locally Administered Deferoxamine Significantly Enhances Fracture Healing in Animal Models: A Systematic Review and Meta-Analysis

*Daniel Müller*, Jens Klotsche, Magdalena B. Kosik, Carsten Perka, Frank Buttgereit, Paula Hoff* and Timo Gaber**

# Supporting Information

Title

## Fracture Fusion on Fast-Forward: Locally Administered Deferoxamine Significantly Enhances Fracture Healing in Animal Models: A Systematic Review and Meta-Analysis

**Authors**

Daniel Müller, Jens Klotsche, Magdalena B. Kosik, Carsten Perka, Frank Buttgereit, Paula Hoff* and Timo Gaber*

*Authors contributed equally

**Corresponding Author**

Paula Hoff, paula.hoff@amedes-group.com, MVZ Endokrinologikum Berlin am Gendarmenmarkt, Friedrichstraße 76/Jägerstraße 61 (Q 207), 10117 Berlin, Germany; T: +49 (0) 30 209156-2290

## Contents:

Identification and Selection Process of Studies
***Table S1:*** Excluded records
***Table S2:*** Excluded full text articles
***Table S3:*** Studies excluded from meta-analysis

Subgroup Analysis
***Figure S1:*** Health state
***Figure S2.1 and S2.2:*** Bone type
***Figure. S3.1 and S3.2:*** Application route
***Figure S4.1 and S4.2:*** Type of fracture defect model
***Figure S5.1 and S5.2:*** Time points
***Figure S6.1 and S6.2:*** Sex
***Figure S7.1 and S7.2:*** Age

Secondary Endpoints
***Figure S8:*** Bone Volume (BV)
***Figure S9:*** Tissue Volume (TV)
***Figure S10:*** Bone Mineral Density (BMD)
***Figure S11:*** Trabecular Separation or Spacing (Tb.Sp.)
***Figure S12:*** Trabecular Thickness (Tb.Th.)

Sensitivity Analysis
***Figure S13:*** Leave-one-out method
***Figure S14:*** Influence of low-quality studies
***Figure S15:*** Influence of small studies
***Figure S16:*** Exploring the influence of small studies with further subdivisions
***Figure S17***: Influence of different measurement device models

Publication Bias
***Figure S18:*** Trim-and-Fill method
***Figure S19:*** Funnel plot with exclusion of small studies
***Figure S20:*** Trim-and-Fill method with exclusion of small studies

Adjusted Search Strategy
***Text S1:*** Adjusted search strategy for WOS (Web of Science)

# Identification and Selection Process of Studies

## ***Table S1:*** Excluded records

| Author | Year | Reason | Title | Journal |
|---|---|---|---|---|
| Agis, H., Watzek, G. and Gruber, R. | 2012 | no fracture | Prolyl hydroxylase inhibitors increase the production of vascular endothelial growth factor by periodontal fibroblasts | Journal of Periodontal Research |
| Alnahwi, A. H., Ait-Mohand, S., Dumulon-Perreault, V., Dory, Y. L. and Guerin, B. | 2020 | no fracture | Promising Performance of 4HMS, a New Zirconium-89 Octadendate Chelator | ACS Omega |
| Alnahwi, A., Ait-Mohand, S., Dumulon-Perreault, V. and Guerin, B. | 2017 | no fracture | Radiolabeling and validation of a new acyclic chelator bearing N-hydroxy-N-methyl succinamide pendant arms for Zirconium-89 radionuclide, superior performance in comparison with DFO | Journal of Nuclear Medicine. Conference: Society of Nuclear Medicine and Molecular Imaging Annual Meeting, SNMMI |
| Alvarez, N. H. and Lewis, J. | 2022 | no fracture, Duplicate | Preclinical development of [<sup>89</sup>Zr]Zr-DFO-Isatuximab as a novel immunoPET tracer for CD38-targeted imaging of multiple myeloma and lymphomas | Nuclear Medicine and Biology |
| Alvarez, N. H., Viray, T. D., Michel, A. and Lewis, J. | 2022 | no fracture, Duplicate | Preclinical Development of 89Zr-DFO-Isatuximab as a Novel ImmunoPET Tracer for CD38-targeted Imaging of Multiple Myeloma and Other Hematological Malignancies | Molecular Imaging and Biology |
| Angelucci, E. and Pilo, F. | 2016 | no fracture | Management of iron overload before, during, and after hematopoietic stem cell transplantation for thalassemia major | Annals of the New York Academy of Sciences |
| Bam, R., Ling, W., Khan, S., Venkateshaiah, S. U., Li, X., Van Rhee, F., Nair, B., Usmani, S., Barlogie, B., Shaughnessy, J. D., Epstein, J., Anaissie, E. J. and Yaccoby, S. | 2011 | no fracture | Deregulated cellular iron metabolism factors mediate iron overload in myeloma cells and osteoclasts, and promote myeloma growth and bone disease | Blood. Conference: 53rd Annual Meeting of the American Society of Hematology, ASH |
| Bansal, A., Pandey, M., Demirhan, Y., Nesbitt, J., Crespo-Diaz, R., Terzic, A., Behfar, A. and DeGrado, T. | 2015 | no fracture | Novel <sup>89</sup>Zr cell-labeling method for PET imaging of cell trafficking | Journal of Nuclear Medicine. Conference: Society of Nuclear Medicine and Molecular Imaging Annual Meeting, SNMMI |
| Bansal, A., Sharma, S., Klasen, B., Rosch, F. and Pandey, M. K. | 2022 | no fracture | Evaluation of different <sup>89</sup>Zr-labeled synthons for direct labeling and tracking of white blood cells and stem cells in healthy athymic mice | Scientific reports |
| Baschant, U., Rauner, M., Balaian, E., Weidner, H., Roetto, A., Platzbecker, U. and Hofbauer, L. C. | 2016 | no fracture | Wnt5a is a key target for the pro-osteogenic effects of iron chelation on osteoblast progenitors | Haematologica |
| Beckford Vera, D. R., Smith, C. C., Bixby, L. M., Glatt, D. M., Dunn, S. S., Saito, R., Kim, W. Y., Serody, J. S., Vincent, B. G. and Parrott, M. C. | 2018 | no fracture | Immuno-PET imaging of tumor-infiltrating lymphocytes using zirconium-89 radiolabeled anti-CD3 antibody in immune-competent mice bearing syngeneic tumors | PLoS ONE |
| Bian, J., Bao, L., Gao, X., Wen, X., Zhang, Q., Huang, J., Xiong, Z., Hong, F. F., Ge, Z. and Cui, W. | 2022 | no fracture (wound) | Bacteria-engineered porous sponge for hemostasis and vascularization | Journal of Nanobiotechnology |
| Bonkovsky, H. L., Healey, J. F., Sinclair, P. R. and Sinclair, J. F. | 1985 | no fracture | Conversion of 5-aminolaevulinate into haem by homogenates of human liver. Comparison with rat and chick-embryo liver homogenates | Biochemical Journal |
| Boros, E., Adams, C., Wilson, J. and Caravan, P. | 2017 | no fracture | Bifunctional desferrichrome derivatives as platform chelators for <sup>89</sup>Zr(IV) | Journal of Nuclear Medicine. Conference: Society of Nuclear Medicine and Molecular Imaging Annual Meeting, SNMMI |
| Boros, E., Holland, J. and Caravan, P. | 2015 | no fracture, conference abstract | Macrocycle based hydroxamate chelators for stable complexation of <sup>89</sup>Zr | Journal of Nuclear Medicine. Conference: Society of Nuclear Medicine and Molecular Imaging Annual Meeting, SNMMI |
| Branitzki-Heinemann, K., Mollerherm, H., Vollger, L. and Von Kockritz-Blickwede, M. | 2016 | no fracture | Formation of neutrophil and mast cell extracellular traps under low oxygen level | Acta Physiologica |
| Buchwalder, C., Jaraquemada-Pelaez, M. G., Rousseau, J., Merkens, H., Rodriguez-Rodriguez, C., Orvig, C., Benard, F., Schaffer, P., Saatchi, K. and Hafeli, U. O. | 2019 | no fracture | Evaluation of the Tetrakis(3-Hydroxy-4-Pyridinone) Ligand THPN with Zirconium(IV): Thermodynamic Solution Studies, Bifunctionalization, and in Vivo Assessment of Macromolecular <sup>89</sup>Zr-THPN-Conjugates | Inorganic chemistry |
| Bystrom, L., Patel, K., Neto, C., Guo, M. and Rivella, S. | 2011 | no fracture | The anti-tumor effects of potential iron celators from vaccinium macrocarpon (Cranberries) in B16-F10 melanoma cells in vitro | American Journal of Hematology |
| Cabra, R., Siegrist, M., Dolder, S. and Hofstetter, W. | 2018 | no fracture | The role of iron in the development and activity of osteoclasts | Calcified Tissue International |
| Caille, F., Naninck, T., Truillet, C., Kuhnast, B. and Chapon, C. | 2021 | no fracture | <sup>89</sup>Zr radiolabeling of antibodies targeting trachea colonization factor A for immuno-PET imaging of Bodetella pertussis in whooping cough infection | Nuclear Medicine and Biology |
| Callens, C., Coulon, S., Naudin, J., Radford-Weiss, I., Boissel, N., Raffoux, E., Dombret, H., Macintyre, E., Moura, I. and Hermine, O. | 2010 | no fracture | Targeting iron homeostasis induces cellular differentiation and synergizes with differentiating agents in acute myeloid leukemia | Haematologica |
| Chang, E. I., Loh, S. A., Ceradini, D. J., Chang, E. I., Lin, S. E., Bastidas, N., Aarabi, S., Chan, D. A., Freedman, M. L., Giaccia, A. J. and Gurtner, G. C. | 2007 | no fracture | Age decreases endothelial progenitor cell recruitment through decreases in hypoxia-inducible factor 1alpha stabilization during ischemia | Circulation |
| Chen, F., Ma, K., Zhang, L., Zanzonico, P., Wiesner, U. and Bradbury, M. | 2017 | no fracture | Multimodality tumor imaging using clinically-translated ultrasmall silica nanoparticles labeled with Zirconium-89: Comparison of radiolabeling strategies | Journal of Nuclear Medicine. Conference: Society of Nuclear Medicine and Molecular Imaging Annual Meeting, SNMMI |
| Chen, F., Madajewski, B., Ma, K., Zhang, L., Zanzonico, P., Quinn, T., Wiesner, U. and Bradbury, M. | 2018 | no fracture | Renally clearable PSMA inhibitors conjugated ultrasmall silica nanoparticles enhance the specific detection of prostate cancer in vivo | Journal of Nuclear Medicine. Conference: Society of Nuclear Medicine and Molecular Imaging Annual Meeting, SNMMI |
| Chen, H., Yan, Y. F., Qi, J., Deng, L. F. and Cui, W. G. | 2017 | no full text published | Sustained delivery of desferrioxamine via liposome carriers in hydrogel for combining angiogenesis and osteogenesis in bone defects reconstruction | Journal of Controlled Release |
| Chen, J., Li, X., Liu, H., Zhong, D., Yin, K., Li, Y., Zhu, L., Xu, C., Li, M. and Wang, C. | 2023 | no fracture | Bone marrow stromal cell-derived exosomal circular RNA improves diabetic foot ulcer wound healing by activating the nuclear factor erythroid 2-related factor 2 pathway and inhibiting ferroptosis | Diabetic Medicine |
| Choesang, T., Li, H., Dussiot, M., Maciel, T., Breda, L., Santos, D. G., Chen, H., Feola, M., Bao, W., Pham, P., Follenzi, A., Li, G., Moura, I. C., Ponka, P., Fleming, R. E., Rivella, S. and Ginzburg, Y. | 2014 | no fracture | Exogenous apo-transferrin increases monoferric transferrin, decreasing cytosolic iron uptake and heme and globin synthesis in beta-thalassemic mice | Blood. Conference: 56th Annual Meeting of the American Society of Hematology, ASH |
| Choi, H., Jin, H., Kim, J. Y., Lim, K. T., Choung, H. W., Park, J. Y., Chung, J. H. and Choung, P. H. | 2014 | no fracture | Hypoxia promotes CEMP1 expression and induces cementoblastic differentiation of human dental stem cells in an HIF-1-dependent manner | Tissue Engineering - Part A |
| Chung, J. H., Kim, Y. S., Noh, K., Lee, Y. M., Chang, S. W. and Kim, E. C. | 2014 | no fracture, human | Deferoxamine promotes osteoblastic differentiation in human periodontal ligament cells via the nuclear factor erythroid 2-related factor-mediated antioxidant signaling pathway | Journal of periodontal research |
| Clejan, L. A. and Cederbaum, A. I. | 1992 | no fracture | Structural determinants for alcohol substrates to be oxidized to formaldehyde by rat liver microsomes | Archives of Biochemistry and Biophysics |
| Clinkenbeard, E., Edwards, D., Wright, C. and Thompson, W. | 2020 | no fracture, mouse mesenchymal cells | Differential iron requirements of osteoblast and adipocyte differentiation | Journal of Bone and Mineral Research |
| De Los Toyos, J. R., Menendez, P., Sampedro, A. and Hardisson, C. | 1992 | no fracture | Yersinia enterocolitica serotype 0:3-induced arthritis in mice: Microbiological and histopathological information | Apmis |
| de Rosales, R. T. M., Khan, A., Man, F., Minino, A. C., Kim, J. and Pellico, J. | 2022 | no fracture | [<sup>89</sup>Zr]Zr(oxinate)<inf>4</inf> radiolabelling of human neutrophil extracellular microvesicles (EVs): in vivo PET imaging reveals extensive uptake in lymph nodes and brain in a mouse model of rheumatoid arthritis | Nuclear Medicine and Biology |
| Deri, M. A., Ponnala, S., Zeglis, B. M., Pohl, G., Dannenberg, J. J., Lewis, J. S. and Francesconi, L. C. | 2014 | no fracture | Alternative chelator for <sup>89</sup>Zr radiopharmaceuticals: Radiolabeling and evaluation of 3,4,3-(LI-1,2-HOPO) | Journal of Medicinal Chemistry |
| Dewulf, J., Elvas, F. and Van Den Wyngaert, T. | 2019 | no fracture | Radiolabelling and preliminary evaluation of <sup>89</sup>Zr-DFO-denosumab, a novel antibody-based radiopharmaceutical for imaging the receptor activator of the nuclear factor k B ligand (RANKL) in the tumour microenvironment | Journal of Labelled Compounds and Radiopharmaceuticals |
| Dewulf, J., Vangesteh, C., Elvas, F. and Van Den Wyngaert, T. | 2021 | no fracture | [89Zr]Zr-DFO-Denosumab immuno-PET validation in thetumor micro-environment of human head-and-necksquamous UM-SCC-22B xenografts | Journal of Nuclear Medicine. Conference: Society of Nuclear Medicine and Molecular Imaging Annual Meeting, SNMMI |

| | | | | |
|---|---|---|---|---|
| Ding, J., Wang, X., Chen, B., Zhang, J. and Xu, J. | 2019 | no fracture (wound healing) | Exosomes Derived from Human Bone Marrow Mesenchymal Stem Cells Stimulated by Deferoxamine Accelerate Cutaneous Wound Healing by Promoting Angiogenesis | BioMed Research International |
| Ding, Z., Zhou, M., Zhou, Z., Zhang, W., Jiang, X., Lu, X., Zuo, B., Lu, Q. and Kaplan, D. L. | 2019 | no fracture (wound healing) | Injectable Silk Nanofiber Hydrogels for Sustained Release of Small-Molecule Drugs and Vascularization | ACS Biomaterials Science and Engineering |
| Dmochowska, N., Campaniello, M., Mavrangelos, C., Wardill, H., Takhar, P., Keller, M., Tieu, W. and Hughes, P. | 2018 | no fracture | In vivo PET imaging of innate immune responses in murine DSS colitis | Neurogastroenterology and Motility. Conference: 3rd Meeting of the Federation of Neurogastroenterology and Motility and Postgraduate Course on Gastrointestinal Motility, FNM |
| Donneys, A., Ahsan, S., Nelson, N. S. and Buchman, S. R. | 2013 | Reply to a letter to the editor from Namazi et al. | Reply: Deferoxamine Restores Callus Size, Mineralization, and Mechanical Strength in Fracture Healing after Radiotherapy | Plastic and Reconstructive Surgery |
| Donneys, A., Subramanian, C., Lynn, J., Urlaub, K., Kovatch, K., Uygur, H. S., Cohen, M. S. and Buchman, S. R. | 2018 | no fracture (tumor growth) | The propitious dual roles of deferoxamine in head and neck cancer management | Cancer Research. Conference |
| Duarte, D., Hawkins, E. D., Akinduro, O., Ang, H., De Filippo, K., Kong, I. Y., Haltalli, M., Ruivo, N., Straszkowski, L., Vervoort, S. J., McLean, C., Weber, T. S., Khorshed, R., Pirillo, C., Wei, A., Ramasamy, S. K., Kusumbe, A. P., Duffy, K., Adams, R. H., Purton, L. E., Carlin, L. M. and Lo Celso, C. | 2018 | no fracture | Inhibition of Endosteal Vascular Niche Remodeling Rescues Hematopoietic Stem Cell Loss in AML | Cell Stem Cell |
| Duvenhage, J., Ebenhan, T., Garny, S., Hernandez Gonzalez, I., Leyva Montana, R., Price, R., Birkholtz, L. M. and Zeevaart, J. R. | 2020 | no fracture | Molecular Imaging of a Zirconium-89 Labeled Antibody Targeting Plasmodium falciparum-Infected Human Erythrocytes | Molecular Imaging and Biology |
| Duvenhage, J., Garny, S., Ebenhan, T., Bester, C., Buntting, H., Van Wyk, D., Sathekge, M. M., Birkholtz, L. M. and Zeevaart, J. R. | 2016 | no fracture | Zirconium-89 radiolabeling and preclinical imaging of a Plasmodium-specific antibody bioconjugate aimed at the clinical tracking of malaria infections | Molecular Imaging and Biology |
| Egan, C. G. | 2013 | no fracture | Potential therapeutic use of deferoxamine and mesenchymal stem cells in type-1 diabetes: Assembling another piece of the jigsaw, in what is a complex puzzle | Expert Opinion on Biological Therapy |
| Ekstrom, G., Norsten, C., Cronholm, T. and Ingelman-Sundberg, M. | 1987 | no fracture | Cytochrome P-450 dependent ethanol oxidation. Kinetic isotope effects and absence of stereoselectivity | Biochemistry |
| Fay, R. and Holland, J. | 2021 | no fracture | Sortase mediated conjugation and <sup>89</sup>Zr-radiolabelling of the HER2 neu targeting G<inf>3</inf>-DARPin for in vivo PET imaging in BT-474-xenografts | Nuclear Medicine and Biology |
| Foster, A., Kumar, R., Nigam, S., McCarl, L., Edinger, R., Pollack, I., Anderson, C., Edwards, W. and Kohanbash, G. | 2019 | no fracture | ImmunoPET imaging of glioma-infiltrating myeloid cells using Zirconium-89-labeled anti-CD11b antibody | Journal for ImmunoTherapy of Cancer. Conference: 34th Annual Meeting and Pre Conference Programs of the Society for Immunotherapy of Cancer Part |
| Gao, C. and Xu, Y. | 2012 | no fracture, unfractured osteoporotic bone investigated | Iron chelator deferoxamine decreases serum osteocalcin concentration and negativelyaffects boneinovariectomized mouse | Osteoporosis International |
| Gess, B., Hofbauer, K. H., Wenger, R. H., Lohaus, C., Meyer, H. E. and Kurtz, A. | 2003 | no fracture | The cellular oxygen tension regulates expression of the endoplasmic oxidoreductase ERO1-Lalpha | European Journal of Biochemistry |
| Ghai, A., Maji, D., Cho, N., Chanswangphuwana, C., Rettig, M., DiPersio, J., Akers, W., Dehdashti, F., Achilefu, S., Vij, R. and Shokeen, M. | 2017 | no fracture, Duplicate | Preclinical development of CD38-Targeted [89Zr] Zr-DFO-daratumumab for imaging multiple myeloma | Indian Journal of Nuclear Medicine |
| Ghai, A., Maji, D., Cho, N., Chanswangphuwana, C., Rettig, M., Shen, D., DiPersio, J., Akers, W., Dehdashti, F., Achilefu, S., Vij, R. and Shokeen, M. | 2018 | no fracture, Duplicate | Preclinical development of CD38-Targeted [<sup>89</sup>Zr]Zr-DFO-Daratumumab for Imaging Multiple Myeloma | Journal of Nuclear Medicine |
| Ghai, A., Zheleznyak, A., Grabowska, D., Black, K., Prion, J., Vij, K., Vij, R., Dipersio, J., Shokeen, M. and Achilefu, S. | 2021 | no fracture | Therapeutic activation of TiO-Tf-TC nanoparticles byZr-daratumumab in a multiple myeloma tumor model | Journal of Nuclear Medicine. Conference: Society of Nuclear Medicine and Molecular Imaging Annual Meeting, SNMMI |
| Glaus, C., Ikotun, O., Kazules, T., Moriguchi, J., Zhang, S., Thangaraj, B., O'Neill, J., Ketchem, R. R., Doherty, E. M., Sinclair, A., Miranda, L. and Moody, G. | 2014 | no fracture | Quantification of Bi-specific T-cell Engager (BiTE) accumulation in solid tumor xenografts and normal tissues using positron emission tomography | Molecular Imaging and Biology. Conference |
| Gonzalez-Arjona, M. | 2021 | no fracture | 89Zr-alphaTox immunotracer for the in vivo detection of S. aureus infection in osteoarthritis model | Molecular Imaging and Biology |
| Guillou, A., Ouadi, A. and Holland, J. P. | 2022 | no fracture | Heptadentate chelates for <sup>89</sup>Zr-radiolabelling of monoclonal antibodies | Inorganic Chemistry Frontiers |
| Guo, C., Yang, K., Yan, Y., Yan, D., Cheng, Y., Yan, X., Qian, N., Zhou, Q., Chen, B., Jiang, M., Zhou, H., Li, C., Wang, F., Qi, J., Xu, X. and Deng, L. | 2019 | no fracture | SF-deferoxamine, a bone-seeking angiogenic drug, prevents bone loss in estrogen-deficient mice | Bone |
| Guo, J. P., Pan, J. X., Xiong, L., Xia, W. F., Cui, S. and Xiong, W. C. | 2015 | no fracture | Iron chelation inhibits osteoclastic differentiation in vitro and in Tg2576 mouse model of Alzheimer's disease | PLoS ONE |
| Heath, J. L., Weiss, J., Scotland, P. B., Lavau, C. P. and Wechsler, D. S. | 2012 | no fracture | Iron deprivation impairs proliferation of Calm-AF10 leukemia cells in vitro and in vivo | Blood. Conference: 54th Annual Meeting of the American Society of Hematology, ASH |
| Henry, K., Pandya, D., Nikunj, B., Nagle, V., Dilling, T., Wadas, T. and Lewis, J. | 2019 | no fracture | Teaching old ligands new tricks: macrocyclic chelators for zirconium-89 radiochemistry | Nuclear Medicine and Biology |
| Hernandez, R., Thickens, A., Rosenkrans, Z., Lambert-Lepesevich, L., Kink, J., Aluicio-Sarduy, E., Pinchuk, A. and Engle, J. | 2023 | no fracture | Highly efficient, biocompatible radiolabeling method for in vivo cell tracking | European Journal of Nuclear Medicine and Molecular Imaging |
| Higashida, K., Inoue, S. and Nakai, N. | 2020 | no fracture | Iron deficiency attenuates protein synthesis stimulated by branched-chain amino acids and insulin in myotubes | Biochemical and Biophysical Research Communications |
| Holland, J., Klingler, S., Patra, M. and Eichenberger, L. | 2019 | no fracture | Simultaneous photochemical conjugation and <sup>89</sup>Zr-radiolabelling of antibodies for immuno-PET | Journal of Labelled Compounds and Radiopharmaceuticals |
| Hou, J., Yamada, S., Kajikawa, T., Ozaki, N., Awata, T., Yamaba, S., Fujihara, C. and Murakami, S. | 2014 | no fracture | Iron plays a key role in the cytodifferentiation of human periodontal ligament cells | Journal of periodontal research |
| Houghton, J. L., Lyashchenko, S. K., Sawada, R., Zanzonico, P., Rudge, S., Scholz, W. W., Maffuid, P. and Lewis, J. S. | 2016 | no fracture | Optimization and IND enabling investigations of MVT-2163 (89Zr-DFO-5B1) leading to First-in-Human readiness | Cancer Research. Conference: AACR Special Conference on Pancreatic Cancer: Advances in Science and Clinical Care |
| Ishikawa, Y., Maeda, M., Li, M., Lee, S. U., Feldstein, J. T., Kiyoi, H., Naoe, T., Weiss, M. J., Heuser, J. E. and Maeda, T. | 2011 | no fracture | Clathrin assembly protein CALM is necessary for leukemia cell proliferation and erythroid development via regulating transferrin receptor endocytosis | Blood. Conference: 53rd Annual Meeting of the American Society of Hematology, ASH |
| Jablonski, G., Klem, K. H., Danielsen, C. C., Mosekilde, L. and Gordeladze, J. O. | 1996 | no fracture | Aluminium-induced bone disease in uremic rats: Effect of deferoxamine | Bioscience Reports |
| Jung, M., Sola, A., Hughes, J., Kluth, D. C., Vinuesa, E., Vias, J. L., Perez-Ladaga, A. and Hotter, G. | 2012 | no fracture | Infusion of IL-10-expressing cells protects against renal ischemia through induction of lipocalin-2 | Kidney International |
| Jung, M., Sola, A., Hughes, J., Kluth, D. C., Vinuesa, E., Vin as, J. L., Perez, A. and Hotter, G. | 2011 | no fracture | AdIL-10-transduced bone marrow-derived macrophages protects from renal ischemia via iron and Lipocalin-2 | European Journal of Clinical Investigation |
| Kagoya, Y., Arai, S., Yoshimi, A., Tsuruta-Kishino, T., Kataoka, K. and Kurokawa, M. | 2013 | no fracture | JAK2V617F mutation evokes paracrine DNA damage to adjacent normal cells via secretion of lipocalin-2 | Blood. Conference: 55th Annual Meeting of the American Society of Hematology, ASH |
| Kakkar, R., Pierre, S., Das, A., Samidurai, A., Eriksson, N., Akerblom, A., Xie, Z., Wallentin, L. C., Kukreja, R. C. and Devalaraja, M. | 2019 | no fracture | A functional polymorphism in matriptase-2, rs855791, modifies Interleukin-6 mediated cardiovascular risk | Circulation. Conference: American Heart Association Scientific Sessions, AHA |
| Kalay, E., Ermutlu, C., Yeniguel, A. E., Yalcinkaya, U. and Sarisoezen, B. | 2022 | rabbits | Effect of bone morphogenic protein-2 and desferoxamine on distraction osteogenesis Injury-International | Journal of the Care of the Injured |
| Key Jr, L. L., Wolf, W. C., Gundberg, C. M. and Ries, W. L. | 1994 | no fracture | Superoxide and bone resorption | Bone |
| Key, L. L., Jr., Ries, W. L., Glasscock, H., Rodriguiz, R. and Jaffe, H. | 1992 | no fracture, main focus on another agent | Osteoclastic superoxide generation: taking control of bone resorption using modulators of superoxide concentrations | International Journal of Tissue Reactions |
| Khoshlahni, N., Sagha, M., Mirzapour, T., Zarif, M. N. and Mohammadzadeh-Vardin, M. | 2020 | no fracture | Iron depletion with deferoxamine protects bone marrow-derived mesenchymal stem cells against oxidative stress-induced apoptosis | Cell Stress and Chaperones |
| Kim, C. H. and Leitch, H. A. | 2021 | no fracture | Iron overload-induced oxidative stress in myelodysplastic syndromes and its cellular sequelae | Critical Reviews in Oncology Hematology |
| Klein, G. L. | 1989 | no fracture | Aluminum in parenteral products: Medical perspective on large and small volume parenterals | Journal of Parenteral Science and Technology |

| | | | | |
|---|---|---|---|---|
| Koizumi, M., Endo, K., Kunimatsu, M., Sakahara, H., Nakashima, T., Kawamura, Y., Watanabe, Y., Ohmomo, Y., Arano, Y., Yokoyama, A. and Torizuka, K. | 1987 | no fracture | Preparation of <sup>67</sup>Ga-labeled antibodies using deferoxamine as a bifunctional chelate. An improved method | Journal of Immunological Methods |
| Kuo, N., Cunha, J. B., Elenbaas, J. S., Feng, Y., Maitra, D., Shavit, J. and Omary, M. B. | 2020 | no fracture, zebrafish and murine hepatocytes used | High Throughput Screening Identifies an Otc Drug That Ameliorates Experimental Erythropoietic Protoporphyria | Gastroenterology |
| Ladungova, A., Busa, D., Lodhi, Y., Hyl, J., Culen, M. and Smida, M. | 2022 | no fracture | Identification of Novel Therapeutic Options for Venetoclax-Resistant Aml Cells through Drug Repurposing | HemaSphere |
| Li, H., Luo, B., Wen, W., Zhou, C., Tian, L. and Ramakrishna, S. | 2017 | no fracture | Deferoxamine immobilized poly(D,L-lactide) membrane via polydopamine adhesive coating: The influence on mouse embryo osteoblast precursor cells and human umbilical vein endothelial cells | Materials science & engineering |
| Li, J., Fan, L. H., Yu, Z. F., Dang, X. Q. and Wang, K. Z. | 2015 | rabbit | The effect of deferoxamine on angiogenesis and bone repair in steroid-induced osteonecrosis of rabbit femoral heads | Experimental Biology and Medicine |
| Liu, C., Tsai, A. L., Li, P. C., Huang, C. W. and Wu, C. C. | 2017 | no fracture | Endothelial differentiation of bone marrow mesenchyme stem cells applicable to hypoxia and increased migration through Akt and NFkappaB signals | Stem Cell Research and Therapy |
| Loh, S. A., Ceradini, D. J., Chang, E. I., Lin, S. E., Bastidas, N., Aarabi, S., Chan, D. A., Freedman, M. L., Giaccia, A. J. and Gurtner, G. C. | 2007 | no fracture (flap) | Age decreases endothelial progenitor cell recruitment through decreases in hypoxia-inducible factor 1alpha stabilization during ischemia | Circulation |
| Lohrmann, C., O'Reilly, E., J, O. D., Yu, K. H., Pandit-Taskar, N., Lyashchenko, S., Ruan, S., Wu, J., DeNoble, P., Carrasquillo, J., Schmidtlein, C., Teng, R., Lowery, M. A., Varghese, A., Estrella, H., Scholz, W., Maffuid, P., Lewis, J. and Weber, W. | 2017 | no fracture, human | First-in-human study of <sup>89</sup>Zr-DFO-HuMab-5B1 (MVT-2163) PET/CT imaging with and without HuMab-5B1 (MVT-5873) in patients with pancreatic cancer and other CA 19-9 positive malignancies | Journal of Nuclear Medicine. Conference: Society of Nuclear Medicine and Molecular Imaging Annual Meeting, SNMMI |
| Luther, J., Peters, S., Baldauf, C., Amling, M., Schinke, T. and David, J. P. | 2017 | no fracture | CFOS over-expression causes a tumor-independent lipodystrophy | Calcified Tissue International |
| Lv, Q., Niu, H., Yue, L., Liu, J., Yang, L., Liu, C., Jiang, H., Dong, S., Shao, Z., Xing, L. and Wang, H. | 2020 | no fracture | Abnormal Ferroptosis in Myelodysplastic Syndrome | Frontiers in Oncology |
| Ma, J., Wang, A., Zhang, H., Liu, B., Geng, Y., Xu, Y., Zuo, G. and Jia, P. | 2022 | no fracture | Iron overload induced osteocytes apoptosis and led to bone loss in Hepcidin<sup>-/-</sup> mice through increasing sclerostin and RANKL/OPG | Bone |
| Ma, M. T., Meszaros, L. K., Paterson, B. M., Berry, D. J., Cooper, M. S., Ma, Y., Ballinger, J. R., Hider, R. C. and Blower, P. J. | 2013 | no fracture | A tripodal tris(hyroxypyrdinone) ligand for immunoconjugate PET imaging with zirconium-89 | European Journal of Nuclear Medicine and Molecular Imaging |
| Matsunaga, K., Fujisawa, K., Takami, T., Burganova, G., Sasai, N., Matsumoto, T., Yamamoto, N. and Sakaida, I. | 2019 | no fracture | NUPR1 acts as a pro-survival factor in human bone marrow-derived mesenchymal stem cells and is induced by the hypoxia mimetic reagent deferoxamine | Journal of Clinical Biochemistry and Nutrition |
| Mendonca, D. B. S., Mendonca, G., Aragao, F. J. L. and Cooper, L. F. | 2011 | no fracture | NF-kappaB suppresses HIF-1alpha response by competing for P300 binding | Biochemical and Biophysical Research Communications |
| Mendonca, L. E., Pernet, E., Khan, N., Sanz, J., Kaufmann, E., Downey, J., Grant, A., Orlova, M., Schurr, E., Krawczyk, C., Jones, R. G., Barreiro, L. B. and Divangahi, M. | 2022 | no fracture, human | Human alveolar macrophage metabolism is compromised during Mycobacterium tuberculosis infection | Frontiers in immunology |
| Momeni, A., Rapp, S., Donneys, A., Buchman, S. R. and Wan, D. C. | 2016 | clinical report, human | Clinical Use of Deferoxamine in Distraction Osteogenesis of Irradiated Bone | Journal of Craniofacial Surgery |
| Morais, M., Ma, M., Foley, C., Cusnir, R. and Lange, J. | 2019 | no fracture | Tetrakis(3,4-hydroxypyridinone) bifunctional chelators for zirconium-89 imaging of antibodies | Nuclear Medicine and Biology |
| Muns, J. A., Montserrat, V., Houthoff, H. J., Codee-Van Der Schilden, K., Zwaagstra, O., Sijbrandi, N. J., Merkul, E. and Van Dongen, G. A. M. S. | 2018 | no fracture | In vivo characterization of platinum (II)-based linker technology for the development of antibody-drug conjugates: Taking advantage of dual labeling with <sup>195m</sup>Pt and <sup>89</sup>Zr | Journal of Nuclear Medicine |
| Nai, A., Lidonnici, M. R., Federico, G., Pettinato, M., Artuso, I., Mandelli, G., Ferrari, G., Camaschella, C., Carlomagno, F. and Silvestri, L. | 2018 | no fracture | Investigating the role of NCOA4 in acute and chronic iron release from stores | HemaSphere |
| Namazi, H. and Majd, Z. | 2013 | no intervention study, letter to the editor | Deferoxamine Restores Callus Size, Mineralization, and Mechanical Strength in Fracture Healing after Radiotherapy | Plastic and Reconstructive Surgery |
| Nigam, S., McCarl, L., Edinger, R. S., Kumar, R., Anderson, C., Kohanbash, G. and Edwards, W. B. | 2019 | no fracture | Development of MDSC-CD11b tracer for immune PET imaging in glioblastoma model | Journal of Nuclear Medicine. Conference |
| Nocka, K. H. and Pelus, L. M. | 1988 | no fracture | Cell cycle specific effects of deferoxamine on human and murine hematopoietic progenitor cells | Cancer Research |
| Ono-Uruga, Y., Tozawa, K., Matsuoka, S., Horiuchi, T., Okamoto, S., Murata, M., Ikeda, Y., Suda, T. and Matsubara, Y. | 2014 | no fracture | A novel mechanism of megakaryopoiesis from pre-adipocytes: Involvement of transferrin/ CD71/ TPO pathways | Blood. Conference: 56th Annual Meeting of the American Society of Hematology, ASH |
| Oroujeni, M., Garousi, J., Andersson, K., Lofblom, J., Mitran, B., Orlova, A. and Tolmachev, V. | 2018 | no fracture | Comparative evaluation of anti-EFGR affibody molecules labelled with gallium-68 and zirconium-89 using desferrioxamine B as a chelator | European Journal of Nuclear Medicine and Molecular Imaging |
| Paillard, A., Pandya, D. N., Zhu, D., Yuan, H., Mintz, A. and Wadas, T. J. | 2013 | no fracture (tumor) | Imaging fibroblast activation protein alpha expression using PET and Cerenkov luminescence imaging | Molecular Imaging and Biology |
| Panikar, S. S., Keltee, N. C. and Ribeiro Pereira, P. M. | 2022 | no fracture | Targeting Tumor Heterogeneity with Multi-RTK Clicking Antibodies | Molecular Imaging and Biology |
| Past, W. L. | 1964 | no fracture, in vitro | The uptake of ef<sup>54</sup> by osseous tissue arising jm vitro | American journal of pathology |
| Perez-Medina, C., Abdel-Atti, D., Zhang, Y., Irwin, C., Binderup, T., Fayad, Z., Lewis, J., Mulder, W. and Reiner, T. | 2014 | no fracture, conference abstract | 89Zr-Labeled liposomes as theranostic agents | Journal of Nuclear Medicine. Conference: Society of Nuclear Medicine and Molecular Imaging Annual Meeting, SNMMI |
| Placa, D. L., Chean, J., Salazar, F., Olafsen, T., Wu, A. M. and Zettlitz, K. A. | 2022 | no fracture | 89Zr-ImmunoPET visualizes the optimized pharmacokinetics of anti-PSCA scFv-Fc proteins for radioimmunotherapy of pancreatic cancer | Molecular Imaging and Biology |
| Platt, I., Rowe, G. C., Lotinun, S., Horne, W., Atfi, A. and Baron, R. | 2010 | no fracture, no DFO | AP-1 proteins affect bone formation negatively via both AP-1 transcriptional activity and interaction with beta-catenin whereas truncated isoforms (FosB/2FosB) do not | Journal of Bone and Mineral Research |
| Prat, A. G. and Turrens, J. F. | 1990 | no fracture | Ascorbate- and hemoglobin-dependent brain chemiluminescence | Free Radical Biology and Medicine |
| Pringle, T. A., Knight, J., Luli, S., Chan, C. D., Rankin, K. S. and Blair, H. | 2022 | no fracture, Duplicate | The Development of a Dual-Modality (PET/NIRF) Radioimmunoconjugate for Image-Guided Sarcoma Surgery | Molecular Imaging and Biology |
| Pringle, T., Knight, J., Chan, C., Blair, H., Luli, S. and Rankin, K. | 2022 | no fracture, Duplicate | The development of a dual-modality (PET/NIR) radioimmunoconjugate for image-guided sarcoma surgery | Nuclear Medicine and Biology |
| Quarles, L. D., Wenstrup, R. J., Castillo, S. A. and Drezner, M. K. | 1991 | no fracture | Aluminum-induced mitogenesis in MC3T3-E1 osteoblasts: Potential mechanism underlying neoosteogenesis | Endocrinology |
| Ran, Q., Yu, Y., Chen, W., Shen, X., Mu, C., Yuan, Z., Tao, B., Hu, Y., Yang, W. and Cai, K. | 2018 | no fracture | Deferoxamine loaded titania nanotubes substrates regulate osteogenic and angiogenic differentiation of MSCs via activation of HIF-1alpha signaling | Materials science & engineering |
| Reincke, U., Brookoff, D., Burlington, H., Cronkite, E. P., Pappas, N. and Zanjani, E. | 1978 | no fracture | Susceptibility of hematopoietic stem cells (CFU-s) to <sup>55</sup>Fe radiation damage | Radiation Research |
| Ries, W. L., Key Jr, L. L. and Rodriguiz, R. M. | 1992 | no fracture | Nitroblue tetrazolium reduction and bone resorption by osteoclasts in vitro inhibited by a manganese-based superoxide dismutase mimic | Journal of Bone and Mineral Research |
| Salih, A. K., Dominguez Garcia, M., Raheem, S. J., Ahiahonu, W. K. and Price, E. W. | 2023 | no fracture | DFO-Km: A Modular Chelator as a New Chemical Tool for the Construction of Zirconium-89-Based Radiopharmaceuticals | Inorganic chemistry |
| Sarrett, S., Coquillat, A. S., Hosny, M., Kunihiro, A., Lastwika, K., Lampe, P. and Zeglis, B. | 2022 | no fracture | Visualizing SCLC in advanced murine models via immunoPET with a <sup>89</sup>Zr-labeled radioimmunoconjugate | Nuclear Medicine and Biology |
| Shao, Z., Yin, T., Jiang, J., He, Y., Xiang, T. and Zhou, S. | 2023 | no fracture (wound healing) | Wound microenvironment self-adaptive hydrogel with efficient angiogenesis for promoting diabetic wound healing | Bioactive Materials |
| Sheng, H., Lao, Y., Zhang, S., Ding, W., Lu, D. and Xu, B. | 2020 | no fracture | Combined Pharmacotherapy with Alendronate and Desferoxamine Regulate the Bone Resorption and Bone Regeneration for Preventing Glucocorticoids-Induced Osteonecrosis of the Femoral Head | BioMed Research International |
| Sheng, W. and Youjia, X. | 2016 | no fracture | The effect and mechanism of deferoxamine on iron accumulation-induced bone loss in ovariectomized C57BL/6J mice | American Journal of Hematology |
| Shin, J. A., Kim, Y. A., Kim, H. W., Kim, H. S., Lee, K. E., Kang, J. L. and Park, E. M. | 2018 | no fracture | Iron released from reactive microglia by noggin improves myelin repair in the ischemic brain | Neuropharmacology |
| Shrivastav, A., Dixit, M., Kumari, S. and Gambhir, S. | 2022 | no fracture | Modified deferoxamine derivatives as chelator with <sup>89</sup>Zr and its application as PET imaging agent | Indian Journal of Nuclear Medicine |
| Simonetta, F., Alam, I. S., Lohmeyer, J. K., Sahaf, B., Good, Z., Chen, W., Xiao, Z., Hirai, T., Scheller, L., Engels, P., Vermesh, O., Robinson, E., Haywood, T., Sathirachindra, A., Baker, J., Malipatlolla, M. B., Schultz, L. M., Spiegel, J. Y., Lee, J. T., Miklos, D. B., Mackall, C. L., Gambhir, S. S. and Negrin, R. S. | 2020 | no fracture | Molecular Imaging of Chimeric Antigen Receptor T Cells By ICOS-Immunopet | Blood |
| Smith, J. K., Carden, D. L., Grisham, M. B., Granger, D. N. and Korthuis, R. J. | 1989 | no fracture, Duplicate | Role of iron in postischemic microvascular injury | American Journal of Physiology - Heart and Circulatory Physiology |
| Smith, J. K., Carden, D. L., Grisham, M. B., Granger, D. N. and Korthuis, R. J. | 1989 | no fracture, Duplicate | Role of iron in postischemic microvascular injury | American Journal of Physiology |

| | | | | |
|---|---|---|---|---|
| Soon, E., Crosby, A., Southwood, M., Pepke-Zaba, J., Upton, P. and Morrell, N. W. | 2015 | no fracture | Loss of BMPR2 leads to dysfunctional iron metabolism and increased inflammation | American Journal of Respiratory and Critical Care Medicine. Conference: American Thoracic Society International Conference, ATS |
| Steiner, S. R. and Philbert, M. A. | 2011 | no fracture | Proteomic identification of carbonylated proteins in 1,3-dinitrobenzene neurotoxicity | NeuroToxicology |
| Tang, A., Li, Y., Yao, Y., Yang, X., Cao, Z., Nie, H. and Yang, G. | 2021 | no fracture (wound healing) | Injectable keratin hydrogels as hemostatic and wound dressing materials | Biomaterials Science |
| Toyos, J. R. D., Menendez, P., Sampedro, A. and Hardisson, C. | 1992 | no fracture | YERSINIA-ENTEROCOLITICA SEROTYPE 0/3-INDUCED ARTHRITIS IN MICE - MICROBIOLOGICAL AND HISTOPATHOLOGICAL INFORMATION | Apmis |
| Trudell, J. R. and Gut, J. | 1989 | no fracture | Properties of enzymes in hepatocytes that convert 5-HPETE or LTA4 into LTB4 | Free Radical Biology & Medicine |
| Truillet, C., Bouleau, A., Nozach, H., Richard, M., Chevaleyre, C., Dubois, S., Kuhnast, B., Evans, M., Specklin, S. and Tran, L. | 2022 | no fracture | Pharmacokinetic preclinical evaluation of radioligation strategies impact on antibody fragment targeting PD-L1 | Nuclear Medicine and Biology |
| Tully, K., Demoin, D., Deri, M., Hatcher, J., Francesconi, L. and Lewis, J. | 2019 | no fracture | Evaluating HOPO chelators with therapeutic radioisotopes for theranostic applications in immunoPET imaging and radioimmunotherapy | Nuclear Medicine and Biology |
| Ulaner, G. A., Sobol, N. B., O'Donoghue, J. A., Kirov, A. S., Riedl, C. C., Min, R., Smith, E., Carter, L. M., Lyashchenko, S. K., Lewis, J. S. and Ola Landgren, C. | 2020 | no fracture, human | CD38-targeted immuno-PET of multiple myeloma: From xenograft models to first-in-human imaging | Radiology |
| Ulaner, G., Sobo, N., aO'Donoghue, J., Burnazi, E., Staton, K., Weber, W., Lyashchenko, S., Lewis, J. and Landgren, C. O. | 2019 | no fracture, Duplicate | Preclinical development and first-inhuman imaging of 89Zr-daratumumab for CD38 targeted imaging of myeloma | Journal of Nuclear Medicine. Conference |
| Ulaner, G., Sobol, N., O'Donoghue, J., Lyashchenko, S., Lewis, J. and Landgren, C. O. | 2019 | no fracture, Duplicate | Synthesis, preclinical analysis, and first-in-human phase I imaging of 89Zr-DFO-daratumumab for CD38 targeted imaging of myeloma | Clinical Lymphoma, Myeloma and Leukemia |
| Vera, D. R. B., Smith, C. C., Bixby, L. M., Glatt, D. M., Dunn, S. S., Saito, R., Kim, W. Y., Serody, J. S., Vincent, B. G. and Parrott, M. C. | 2018 | no fracture | Immuno-PET imaging of tumor-infiltrating lymphocytes using zirconium-89 radiolabeled anti-CD3 antibody in immune-competent mice bearing syngeneic tumors | Plos One |
| Verbeelen, D., Smeyers-Verbeke, J., Van Hooff, F. and De Roy, G. | 1989 | no fracture | Vitamin D, desferrioxamine and aluminum-induced bone disease in uremic rats | Nephron |
| Vinke, J. S. J., Gorter, A. R., Eisenga, M. F., Dam, W. A., van der Meer, P., van den Born, J., Bakker, S. J. L., Hoes, M. F. and de Borst, M. H. | 2023 | no fracture | Iron deficiency is related to lower muscle mass in community-dwelling individuals and impairs myoblast proliferation | Journal of Cachexia, Sarcopenia and Muscle |
| Vinzenz, P., Schrockmair, S., Gruber, R. and Agis, H. | 2015 | no fracture, in vitro | Bone substitute materials supplemented with prolyl hydroxylase inhibitors decrease osteoclastogenesis in vitro | Journal of Biomedical Materials Research - Part B Applied Biomaterials |
| Wang, L., Jia, P., Shan, Y., Hao, Y., Wang, X., Jiang, Y., Yuan, Y., Du, Q., Zhang, H., Yang, F., Zhang, W., Sheng, M. and Xu, Y. | 2017 | no fracture | Synergistic protection of bone vasculature and bone mass by desferrioxamine in osteoporotic mice | Molecular Medicine Reports |
| Wang, L., Zhou, F., Zhang, P., Wang, H., Qu, Z., Jia, P., Yao, Z., Shen, G., Li, G., Zhao, G., Li, J., Mao, Y., Xie, Z., Xu, W. and Xu, Y. | 2017 | no fracture | Human type H vessels are a sensitive biomarker of bone mass | Cell Death and Disease |
| Wu, H., Wang, T., Liang, Y., Chen, L. and Li, Z. | 2024 | no fracture (wound healing) | Self-assembled and dynamic bond crosslinked herb-polysaccharide hydrogel with anti-inflammation and pro-angiogenesis effects for burn wound healing | Colloids and Surfaces B: Biointerfaces |
| Wu, S., Wang, S. and Meng, Y. 2023 Protective Effects and Mechanisms of Quercetin on Osteoporotic Rats via Modulating the Ferroptosis Pathway Journal of Biological Regulators and Homeostatic Agents | 2023 | no fracture | Protective Effects and Mechanisms of Quercetin on Osteoporotic Rats via Modulating the Ferroptosis Pathway | Journal of Biological Regulators and Homeostatic Agents |
| Wyszatko, K., Valliant, J., Sadeghi, S. and Singh, S. | 2021 | no fracture | PET imaging cancer stem cells using a novel zirconium-89labelled fully human anti-CD133 antibody | Journal of Nuclear Medicine. Conference: Society of Nuclear Medicine and Molecular Imaging Annual Meeting, SNMMI |
| Xing, Y., Wang, R., Chen, D., Mao, J., Shi, R., Wu, Z., Kang, J., Tian, W. and Zhang, C. | 2015 | no fracture | COX2 is involved in hypoxia-induced TNF-alpha expression in osteoblast | Scientific reports |
| Xu, C. Z., Lin, T., Zhao, X. L., Gan, Y. C., Huang, J. X., Zhang, J., Zheng, H. B., Pu, C. Y., Lin, R. R., Yan, B., Hu, G. J., Liu, Q. L., Yu, B., Li, S. J. and Hou, H. H. | 2023 | no fracture (tendon-to-bone healing) | Microenvironment responsive hypoxia-mimetic DFO composite hydrogel for on-demand neovascularization to promote tendon-to-bone healing | Composites Part B-Engineering |
| Xu, W., Yu, R., Zhu, X., Li, Z., Jia, J., Li, D., Chen, Y. and Zhang, X. | 2020 | no fracture | Iron-Chelating Agent Can Maintain Bone Homeostasis Disrupted by Iron Overload by Upregulating Wnt/Beta-Catenin Signaling | BioMed Research International |
| Xue, Y., Yang, J., Luo, J., Ren, L., Shen, Y., Dong, D., Fang, Y., Hu, L., Liu, M., Liao, Z., Li, J., Fang, Z. and Shang, P. | 2020 | no fracture | Disorder of Iron Metabolism Inhibits the Recovery of Unloading-Induced Bone Loss in Hypomagnetic Field | Journal of Bone and Mineral Research |
| Xun, X., Qiu, J., Zhang, J., Wang, H., Han, F., Xu, X. and Yuan, R. | 2022 | no fracture, rabbits | Triple-functional injectable liposome-hydrogel composite enhances bacteriostasis and osteo/angio-genesis for advanced maxillary sinus floor augmentation | Colloids and Surfaces B: Biointerfaces |
| Yang, J., Dong, D., Luo, X., Zhou, J., Shang, P. and Zhang, H. | 2020 | no fracture, in vitro | Iron Overload-Induced Osteocyte Apoptosis Stimulates Osteoclast Differentiation Through Increasing Osteocytic RANKL Production In Vitro | Calcified Tissue International |
| Yi, L., Ju, Y., He, Y., Yin, X., Xu, Y. and Weng, T. | 2021 | no fracture | Intraperitoneal injection of Desferal alleviated the age-related bone loss and senescence of bone marrow stromal cells in rats | Stem Cell Research and Therapy |
| Yu, M.-L., Wu, H.-M., Li, G.-F., Hu, M.-Y. and Chen, D. | 2023 | no fracture | Effect of sodium alginate-g-deferoxamine/chitosan microspheres on osteogenic differentiation of rat bone mesenchymal stem cells | Shanghai journal of stomatology |
| Zettlitz, K. A., Salazar, F., Chean, J., Placa, D. L., Ahmed, S., Adhikarla, V., Chen, B. Y., Jaiswal, S., Olafsen, T. and Wu, A. M. | 2022 | no fracture | Antibody fragment-based theranostics targeting prostate stem cell antigen (PSCA): Using 89Zr-immunoPET to guide the development of 177Lu-radioimmunotherapy | Molecular Imaging and Biology |
| Zhang, J., Zheng, L., Wang, Z., Pei, H., Hu, W., Nie, J., Shang, P., Li, B., Hei, T. K. and Zhou, G. | 2019 | no fracture | Lowering iron level protects against bone loss in focally irradiated and contralateral femurs through distinct mechanisms | Bone |
| Zhang, W. B., Li, G. S., Deng, R. X., Deng, L. F. and Qiu, S. J. | 2012 | rabbit | New bone formation in a true bone ceramic scaffold loaded with desferrioxamine in the treatment of segmental bone defect: a preliminary study | Journal of Orthopaedic Science |
| | | | | |

***Table S2:*** Excluded full text articles

| Author | Year | Reason | Title | Journal |
|---|---|---|---|---|
| Donneys, A., Blough, J. T., Nelson, N. S., Perosky, J. E., Deshpande, S. S., Kang, S. Y., Felice, P. A., Figueredo, C., Peterson, J. R., Kozloff, K. M., Levi, B., Chepeha, D. B. and Buchman, S. R. | 2016 | DFO always administered together with ASCs (adipose-derived stem cells), combination therapy -> no adequate control to seperately assess effects of DFO | Translational treatment paradigm for managing non-unions secondary to radiation injury utilizing adipose derived stem cells and angiogenic therapy | Head and Neck |
| Donneys, A., Ettinger, R., Ranganathan, K., Snider, A., Buchman, L., Nelson, N. S., Blough, J. T., Deshpande, S. S., Cohen, M. S. and Buchman, S. R. | 2017 | Conference abstract | Enabled nonvascularized grafting in irradiated bone utilizing a novel implantable deferoxamine nano-particle formulation | Journal of the American College of Surgeons |
| Donneys, A., Farberg, A. S., Tchanque-Fossuo, C. N., Deshpande, S. S. and Buchman, S. R. | 2012 | No BV or BV/TV, only vessel formation investigated | Deferoxamine enhances the vascular response of bone regeneration in mandibular distraction osteogenesis | Plastic and reconstructive surgery |
| Donneys, A., Nelson, N. S., Page, E. E., Deshpande, S. S., Felice, P. A., Tchanque-Fossuo, C. N., Spiegel, J. P. and Buchman, S. R. | 2015 | No µCT | Targeting angiogenesis as a therapeutic means to reinforce osteocyte survival and prevent nonunions in the aftermath of radiotherapy | Head and Neck |
| Donneys, A., Nelson, N. S., Perosky, J. E., Polyatskaya, Y., Rodriguez, J. J., Figueredo, C., Vasseli, C. A., Ratliff, H. C., Deshpande, S. S., Kozloff, K. M. and Buchman, S. R. | 2016 | No BV or BV/TV, only mechanical testing and µCT-angiography | Prevention of radiation-induced bone pathology through combined pharmacologic cytoprotection and angiogenic stimulation | Bone |
| Donneys, A., Weiss, D. M., Deshpande, S. S., Ahsan, S., Tchanque-Fossuo, C. N., Sarhaddi, D., Levi, B., Goldstein, S. A. and Buchman, S. R. | 2013 | No BV or BV/TV, only vessel formation investigated | Localized deferoxamine injection augments vascularity and improves bony union in pathologic fracture healing after radiotherapy | Bone |
| Ettinger, R., Nelson, N., Snider, A., Donneys, A., Hoxie, J., Perosky, J. and Buchman, S. | 2017 | Conference abstract | Mitigation of radiation induced injury in non-vascularized bone graft reconstruction of the murine mandible using a novel deferoxamine nanoparticle | Cleft Palate-Craniofacial Journal |
| Farberg, A. S., Jing, X. L., Monson, L. A., Donneys, A., Tchanque-Fossuo, C. N., Deshpande, S. S. and Buchman, S. R. | 2012 | No BV or BV/TV, only vessel formation investigated | Deferoxamine reverses radiation induced hypovascularity during bone regeneration and repair in the murine mandible | Bone. |
| Farberg, A. S., Sarhaddi, D., Donneys, A., Deshpande, S. S. and Buchman, S. R. | 2014 | No µCT, BV/TV only measured with histology | Deferoxamine enhances bone regeneration in mandibular distraction osteogenesis | Plastic and reconstructive surgery |
| Genetos, D. C., Wong, A., Watari, S. and Yellowley, C. E. | 2010 | no fracture; in vitro with rat material | Hypoxia increases Annexin A2 expression in osteoblastic cells via VEGF and ERK | Bone |
| Grewal, B. S., Keller, B., Weinhold, P. and Dahners, L. E. | 2014 | Relevant outcome (mineralized callus volume = BV) only measured for DFO (received calcium sulfate carrier loaded with DFO) and blank control group and not for carrier group (would be the adequate comparison) -> no adequate comparison group | Evaluating effects of deferoxamine in a rat tibia critical bone defect model | Journal of Orthopaedics |
| Guzey, S., Aykan, A., Ozturk, S., Avsever, H., Karslioglu, Y. and Ertan, A. | 2016 | No BV or BV/TV | The Effects of Desferroxamine on Bone and Bone Graft Healing in Critical-Size Bone Defects | Annals of plastic surgery |
| Han, X., Sun, M., Chen, B., Saiding, Q., Zhang, J., Song, H., Deng, L., Wang, P., Gong, W. and Cui, W. | 2021 | DFO loaded into liposomes utilized as a vector, which is not used in the control groups -> no adequate comparison group | Lotus seedpod-inspired internal vascularized 3D printed scaffold for bone tissue repair | Bioactive Materials |
| Hertzberg, B. P., Holt, J. B., Graff, R. D., Gilbert, S. R. and Dahners, L. E. | 2013 | ex vivo: fetal mouse metatarsal angiogenesis assay | An evaluation of carrier agents for desferoxamine, an up-regulator of vascular endothelial growth factor | Journal of Biomaterials Applications |
| Hu, Y. and Li, H. | 2023 | no fracture (intraperitoneal injections and ground down teeth) | Deferoxamine mesylate enhances mandibular advancement-induced condylar osteogenesis by promoting H-type angiogenesis | Journal of oral rehabilitation |
| Jiang, Z., Wang, H., Qi, G., Jiang, C., Chen, K. and Yan, Z. | 2022 | no fracture | Iron overload-induced ferroptosis of osteoblasts inhibits osteogenesis and promotes osteoporosis: An in vitro and in vivo study | IUBMB Life |
| Jing, X., Du, T., Yang, X., Zhang, W., Wang, G., Liu, X., Li, T. and Jiang, Z. | 2020 | no fracture (Glucocorticoid-induced osteonecrosis of the femoral head) | Desferoxamine protects against glucocorticoid-induced osteonecrosis of the femoral head via activating HIF-1alpha expression | Journal of Cellular Physiology |
| Kuchler, U., Keibl, C., Fugl, A., Schwarze, U. Y., Tangl, S., Agis, H. and Gruber, R. | 2015 | No MicroCT, BV/TV only measured with histology | Dimethyloxalylglycine lyophilized onto bone substitutes increase vessel area in rat calvarial defects | Clinical oral implants research |
| Leatherwood, W. H., Bortner, B. A., Draeger, R. W., Dahners, L. E., Rubin, J. and Weinhold, P. S. | 2020 | no fracture, which is only surrounded by bone substance (intra-medullary femoral implant, osseointegration, peri-implant bone volume measured) | Evaluation of zoledronate, cytochalasin-d, and desferrioxamine on osseointegration in an intra-medullary femoral implant model | Journal of Musculoskeletal Neuronal Interactions |
| Li, H., Liao, L., Hu, Y., Xu, Y., Zhang, Y., Huo, F., Tian, W. and Guo, W. | 2021 | no fracture, lower incisors grinded off and osteoporosis experiment -> no fractures induced | Identification of Type H Vessels in Mice Mandibular Condyle | Journal of dental research |
| Li, Z., Cheng, S., Li, A., Song, C., Jiang, A., Xu, F., Chi, H., Yan, J. and Chen, G. | 2022 | DFO always administered together with BMP2 -> seperate or independent effect of DFO not assessable | Fabrication of BMP-2-peptide-Deferoxamine- and QK-peptide-functionalized nanoscaffolds and their application for bone defect treatment | Journal of Tissue Engineering and Regenerative Medicine |
| Li, Z., Li, S., Yang, J., Ha, Y., Zhang, Q., Zhou, X. and He, C. | 2022 | DFO loaded into ethosomes utilized as a vector, which is not used in the control groups -> no adequate comparison group | 3D bioprinted gelatin/gellan gum-based scaffold with double-crosslinking network for vascularized bone regeneration | Carbohydrate Polymers |
| Liu, C., Tsai, A. L., Chen, Y. C., Fan, S. C., Huang, C. H., Wu, C. C. and Chang, C. H. | 2012 | DFO only used in in vitro experiments and not in vivo (Indometacin) | Facilitation of human osteoblast apoptosis by sulindac and indomethacin under hypoxic injury | Journal of Cellular Biochemistry |

| | | | | |
|---|---|---|---|---|
| Liu, J., Kang, H., Lu, J., Dai, Y. and Wang, F. | 2021 | no fracture, which is only surrounded by bone substance (knee replacement, tibial implant, peri-implant bone formation investigated) | Experimental study of the effects of hypoxia simulator on osteointegration of titanium prosthesis in osteoporotic rats | BMC Musculoskeletal Disorders |
| Qiu, M., Cai, Z., Li, C., Yang, K., Tulufu, N., Chen, B., Cheng, L., Zhuang, C., Liu, Z., Qi, J., Cui, W. and Deng, L. | 2023 | DFO linked to an auxiliary molecule (iminodiacetic acid, SF) as an anchor, which is not used in the control groups (no adequate comparison) | 3D Biomimetic Calcified Cartilaginous Callus that Induces Type H Vessels Formation and Osteoclastogenesis | Advanced science (Weinheim, Baden-Wurttemberg, Germany) |
| Tchanque-Fossuo, C. N., Dahle, S. E., Buchman, S. R. and Isseroff, R. R. | 2016 | no original intervention study | Deferoxamine in wound healing | Journal of Investigative Dermatology |
| Urlaub, K. M., Lynn, J. V., Carey, E. G., Nelson, N. S., Polyatskaya, Y., Donneys, A., Mazzoli, A. C. and Buchman, S. R. | 2018 | No MicroCT, BV/TV only measured with histology | Histologic Improvements in Irradiated Bone Through Pharmaceutical Intervention in Mandibular Distraction Osteogenesis | Journal of Oral and Maxillofacial Surgery |
| Wilson, S. S., Wong, A., Toupadakis, C. A. and Yellowley, C. E. | 2015 | no µCT, no BV or BV/TV, DFO only used in vitro | Expression of angiopoietin-like protein 4 at the fracture site: Regulation by hypoxia and osteoblastic differentiation | Journal of Orthopaedic Research |
| Xing, D., Zuo, W., Chen, J., Ma, B., Cheng, X., Zhou, X. and Qian, Y. | 2022 | First in vivo exp.: alveolar fracture/extracted tooth (wrong fracture type, but relevant outcome measures); second in vivo exp.: rat fenestration mandibular bone defect model with induced S.aureus infection (right fracture type, but no relevant outcome measures) | Spatial Delivery of Triple Functional Nanoparticles via an Extracellular Matrix-Mimicking Coaxial Scaffold Synergistically Enhancing Bone Regeneration | ACS applied materials & interfaces |
| Xu, D., Gan, K., Wu, Z., Wang, Y., Zhang, S., Peng, Y., Fang, X., Wei, H., Zhang, Y., Ma, W. and Chen, J. | 2022 | No µCT, no BV or BV/TV | A Composite Deferoxamine/Black Phosphorus Nanosheet/Gelatin Hydrogel Scaffold for Ischemic Tibial Bone Repair | International Journal of Nanomedicine |
| Yalcin-Ulker, G. M., Gunbatan, M., Duygu, G., Soluk-Tekkesin, M. and Ozcakir-Tomruk, C. | 2023 | no fracture (tooth extraction socket) | Could Local Application of Hypoxia Inducible Factor 1-alpha Enhancer Deferoxamine Be Promising for Preventing of Medication-Related Osteonecrosis of the Jaw? | Biomedicines |
| Yao, Q., Liu, Y., Selvaratnam, B., Koodali, R. T. and Sun, H. | 2018 | No fracture, only in vitro experiments | Mesoporous silicate nanoparticles/3D nanofibrous scaffold-mediated dual-drug delivery for bone tissue engineering | Journal of Controlled Release |
| Yao, Q., Liu, Y., Tao, J., Baumgarten, K. M. and Sun, H. | 2016 | No µCT, only BAF (bone area fraction -> 2D) measured | Hypoxia-Mimicking Nanofibrous Scaffolds Promote Endogenous Bone Regeneration | ACS applied materials & interfaces |
| Zheng, X., Zhang, X., Wang, Y., Liu, Y., Pan, Y., Li, Y., Ji, M., Zhao, X., Huang, S. and Yao, Q. | 2021 | DFO loaded onto GelMA (gelatin methacrylamide), which is not used in the control groups (no adequate comparison) | Hypoxia-mimicking 3D bioglass-nanoclay scaffolds promote endogenous bone regeneration | Bioactive Materials |
| | | | | |

## *Table S3:* Studies excluded from meta-analysis

| Author | Year | Reason | Title | Journal |
|---|---|---|---|---|
| Geng, M., Zhang, Q., Gu, J., Yang, J., Du, H., Jia, Y., Zhou, X. and He, C. | 2021 | No n given | Construction of a nanofiber network within 3D printed scaffolds for vascularized bone regeneration | Biomaterials Science |
| Shen, X., Wan, C., Ramaswamy, G., Mavalli, M., Wang, Y., Duvall, C. L., Lian, F. D., Guldberg, R. E., Eberhart, A., Clemens, T. L. and Gilbert, S. R. | 2009 | No specific n given for calculations in analysis | Prolyl hydroxylase inhibitors increase neoangiogenesis and callus formation following femur fracture in mice | Journal of Orthopaedic Research |
| Stewart, R., Goldstein, J., Eberhardt, A., Gabriel Chu, G. T. M. and Gilbert, S. | 2011 | No specific outcome values given for calculations in analysis, no specific n given for calculations in analysis | Increasing vascularity to improve healing of a segmental defect of the rat femur | Journal of Orthopaedic Trauma |
| | | | | |

# Subgroup Analysis

***Figure S1:*** Health state

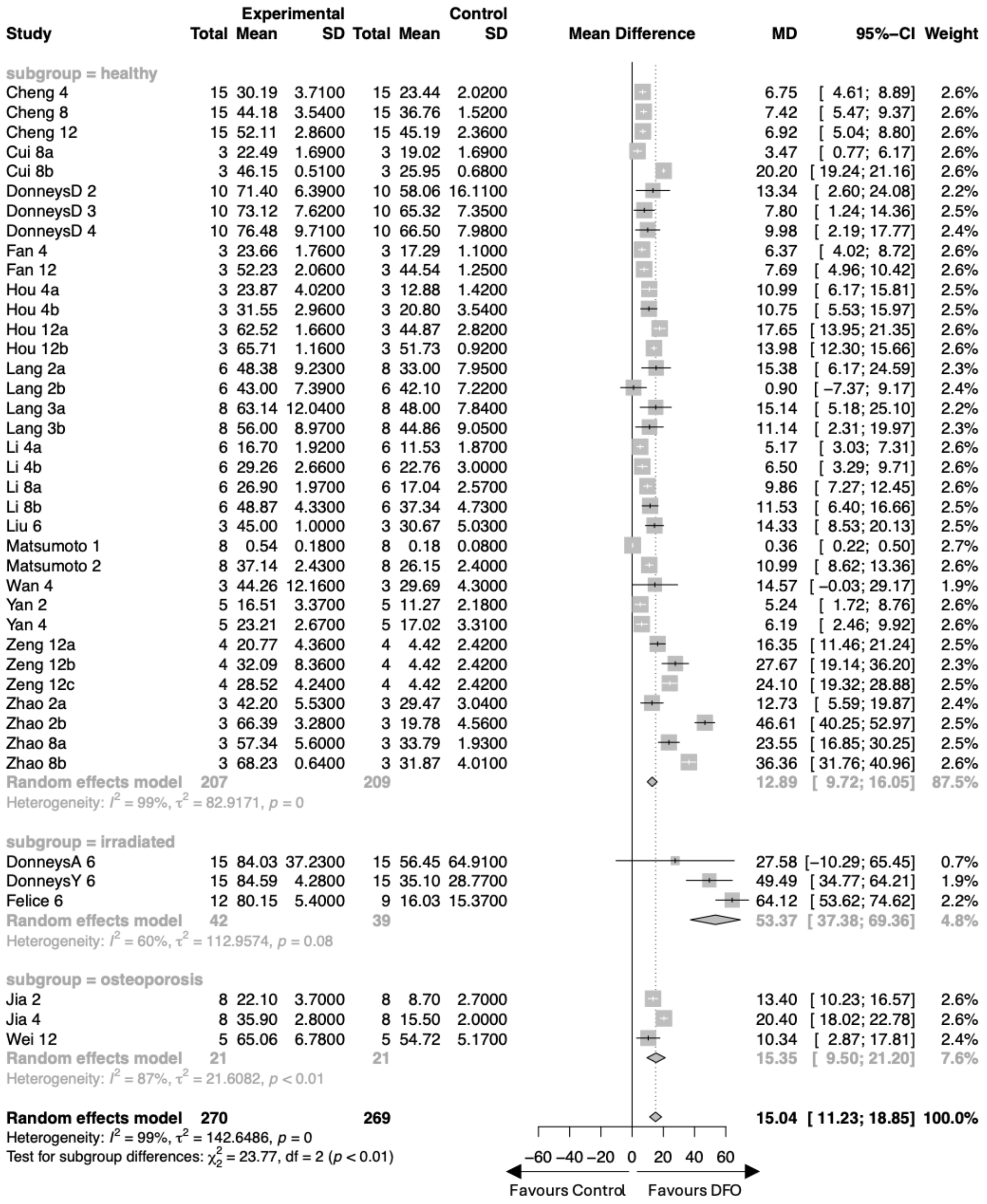


***Forest plot of subgroup analysis for health state with disease models being subdivided into irradiated and osteoporotic bone***

*Figure S2.1 and S2.2:* Bone type

| Study | Experimental Total | Experimental Mean | Experimental SD | Control Total | Control Mean | Control SD | MD | 95%-CI | Weight |
|---|---|---|---|---|---|---|---|---|---|
| subgroup = cranial | | | | | | | | | |
| Cheng 4 | 15 | 30.19 | 3.7100 | 15 | 23.44 | 2.0200 | 6.75 | [ 4.61; 8.89] | 2.6% |
| Cheng 8 | 15 | 44.18 | 3.5400 | 15 | 36.76 | 1.5200 | 7.42 | [ 5.47; 9.37] | 2.6% |
| Cheng 12 | 15 | 52.11 | 2.8600 | 15 | 45.19 | 2.3600 | 6.92 | [ 5.04; 8.80] | 2.6% |
| Cui 8a | 3 | 22.49 | 1.6900 | 3 | 19.02 | 1.6900 | 3.47 | [ 0.77; 6.17] | 2.6% |
| Cui 8b | 3 | 46.15 | 0.5100 | 3 | 25.95 | 0.6800 | 20.20 | [ 19.24; 21.16] | 2.6% |
| DonneysA 6 | 15 | 84.03 | 37.2300 | 15 | 56.45 | 64.9100 | 27.58 | [−10.29; 65.45] | 0.7% |
| DonneysD 2 | 10 | 71.40 | 6.3900 | 10 | 58.06 | 16.1100 | 13.34 | [ 2.60; 24.08] | 2.2% |
| DonneysD 3 | 10 | 73.12 | 7.6200 | 10 | 65.32 | 7.3500 | 7.80 | [ 1.24; 14.36] | 2.5% |
| DonneysD 4 | 10 | 76.48 | 9.7100 | 10 | 66.50 | 7.9800 | 9.98 | [ 2.19; 17.77] | 2.4% |
| DonneysY 6 | 15 | 84.59 | 4.2800 | 15 | 35.10 | 28.7700 | 49.49 | [ 34.77; 64.21] | 1.9% |
| Felice 6 | 12 | 80.15 | 5.4000 | 9 | 16.03 | 15.3700 | 64.12 | [ 53.62; 74.62] | 2.2% |
| Random effects model | 123 | | | 120 | | | 18.77 | [ 7.01; 30.53] | 25.0% |
| Heterogeneity: $I^2 = 98\%$, $\tau^2 = 364.6727$, $p < 0.01$ | | | | | | | | | |
| subgroup = long | | | | | | | | | |
| Fan 4 | 3 | 23.66 | 1.7600 | 3 | 17.29 | 1.1000 | 6.37 | [ 4.02; 8.72] | 2.6% |
| Fan 12 | 3 | 52.23 | 2.0600 | 3 | 44.54 | 1.2500 | 7.69 | [ 4.96; 10.42] | 2.6% |
| Hou 4a | 3 | 23.87 | 4.0200 | 3 | 12.88 | 1.4200 | 10.99 | [ 6.17; 15.81] | 2.5% |
| Hou 4b | 3 | 31.55 | 2.9600 | 3 | 20.80 | 3.5400 | 10.75 | [ 5.53; 15.97] | 2.5% |
| Hou 12a | 3 | 62.52 | 1.6600 | 3 | 44.87 | 2.8200 | 17.65 | [ 13.95; 21.35] | 2.6% |
| Hou 12b | 3 | 65.71 | 1.1600 | 3 | 51.73 | 0.9200 | 13.98 | [ 12.30; 15.66] | 2.6% |
| Jia 2 | 8 | 22.10 | 3.7000 | 8 | 8.70 | 2.7000 | 13.40 | [ 10.23; 16.57] | 2.6% |
| Jia 4 | 8 | 35.90 | 2.8000 | 8 | 15.50 | 2.0000 | 20.40 | [ 18.02; 22.78] | 2.6% |
| Lang 2a | 6 | 48.38 | 9.2300 | 8 | 33.00 | 7.9500 | 15.38 | [ 6.17; 24.59] | 2.3% |
| Lang 2b | 6 | 43.00 | 7.3900 | 6 | 42.10 | 7.2200 | 0.90 | [ −7.37; 9.17] | 2.4% |
| Lang 3a | 8 | 63.14 | 12.0400 | 8 | 48.00 | 7.8400 | 15.14 | [ 5.18; 25.10] | 2.2% |
| Lang 3b | 8 | 56.00 | 8.9700 | 8 | 44.86 | 9.0500 | 11.14 | [ 2.31; 19.97] | 2.3% |
| Li 4a | 6 | 16.70 | 1.9200 | 6 | 11.53 | 1.8700 | 5.17 | [ 3.03; 7.31] | 2.6% |
| Li 4b | 6 | 29.26 | 2.6600 | 6 | 22.76 | 3.0000 | 6.50 | [ 3.29; 9.71] | 2.6% |
| Li 8a | 6 | 26.90 | 1.9700 | 6 | 17.04 | 2.5700 | 9.86 | [ 7.27; 12.45] | 2.6% |
| Li 8b | 6 | 48.87 | 4.3300 | 6 | 37.34 | 4.7300 | 11.53 | [ 6.40; 16.66] | 2.5% |
| Liu 6 | 3 | 45.00 | 1.0000 | 3 | 30.67 | 5.0300 | 14.33 | [ 8.53; 20.13] | 2.5% |
| Matsumoto 1 | 8 | 0.54 | 0.1800 | 8 | 0.18 | 0.0800 | 0.36 | [ 0.22; 0.50] | 2.7% |
| Matsumoto 2 | 8 | 37.14 | 2.4300 | 8 | 26.15 | 2.4000 | 10.99 | [ 8.62; 13.36] | 2.6% |
| Wan 4 | 3 | 44.26 | 12.1600 | 3 | 29.69 | 4.3000 | 14.57 | [ −0.03; 29.17] | 1.9% |
| Wei 12 | 5 | 65.06 | 6.7800 | 5 | 54.72 | 5.1700 | 10.34 | [ 2.87; 17.81] | 2.4% |
| Yan 2 | 5 | 16.51 | 3.3700 | 5 | 11.27 | 2.1800 | 5.24 | [ 1.72; 8.76] | 2.6% |
| Yan 4 | 5 | 23.21 | 2.6700 | 5 | 17.02 | 3.3100 | 6.19 | [ 2.46; 9.92] | 2.6% |
| Zeng 12a | 4 | 20.77 | 4.3600 | 4 | 4.42 | 2.4200 | 16.35 | [ 11.46; 21.24] | 2.5% |
| Zeng 12b | 4 | 32.09 | 8.3600 | 4 | 4.42 | 2.4200 | 27.67 | [ 19.14; 36.20] | 2.3% |
| Zeng 12c | 4 | 28.52 | 4.2400 | 4 | 4.42 | 2.4200 | 24.10 | [ 19.32; 28.88] | 2.5% |
| Zhao 2a | 3 | 42.20 | 5.5300 | 3 | 29.47 | 3.0400 | 12.73 | [ 5.59; 19.87] | 2.4% |
| Zhao 2b | 3 | 66.39 | 3.2800 | 3 | 19.78 | 4.5600 | 46.61 | [ 40.25; 52.97] | 2.5% |
| Zhao 8a | 3 | 57.34 | 5.6000 | 3 | 33.79 | 1.9300 | 23.55 | [ 16.85; 30.25] | 2.5% |
| Zhao 8b | 3 | 68.23 | 0.6400 | 3 | 31.87 | 4.0100 | 36.36 | [ 31.76; 40.96] | 2.6% |
| Random effects model | 147 | | | 149 | | | 14.06 | [ 10.52; 17.59] | 75.0% |
| Heterogeneity: $I^2 = 98\%$, $\tau^2 = 89.2015$, $p = 0$ | | | | | | | | | |
| **Random effects model** | **270** | | | **269** | | | **15.04** | **[ 11.23; 18.85]** | **100.0%** |
| Heterogeneity: $I^2 = 99\%$, $\tau^2 = 142.6486$, $p = 0$ | | | | | | | | | |
| Test for subgroup differences: $\chi^2_1 = 0.57$, df = 1 ($p = 0.45$) | | | | | | | | | |

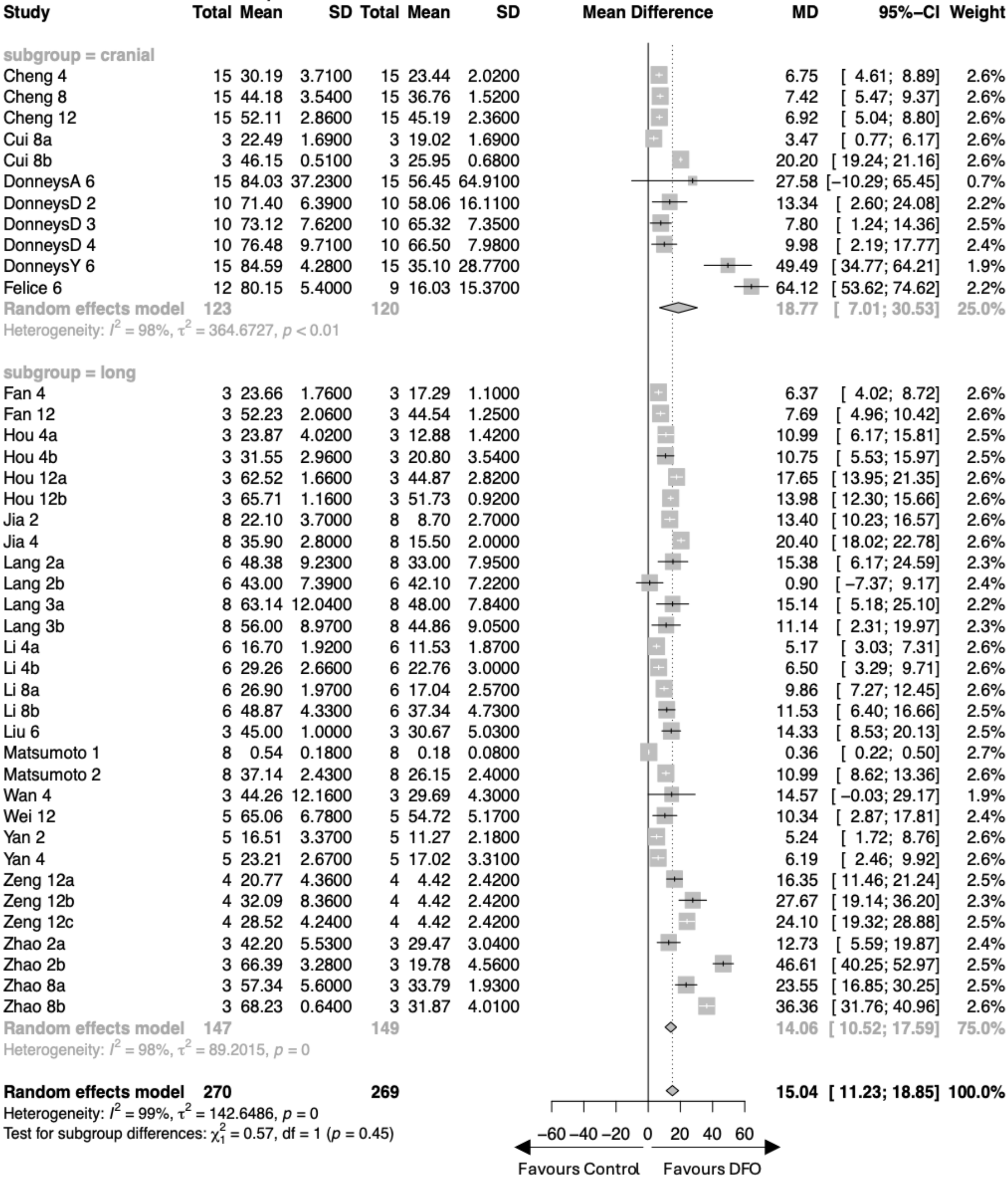


***Figure S2.1: Forest plot for analyzing the bone type subgroups of cranial and long bones, including disease model studies***

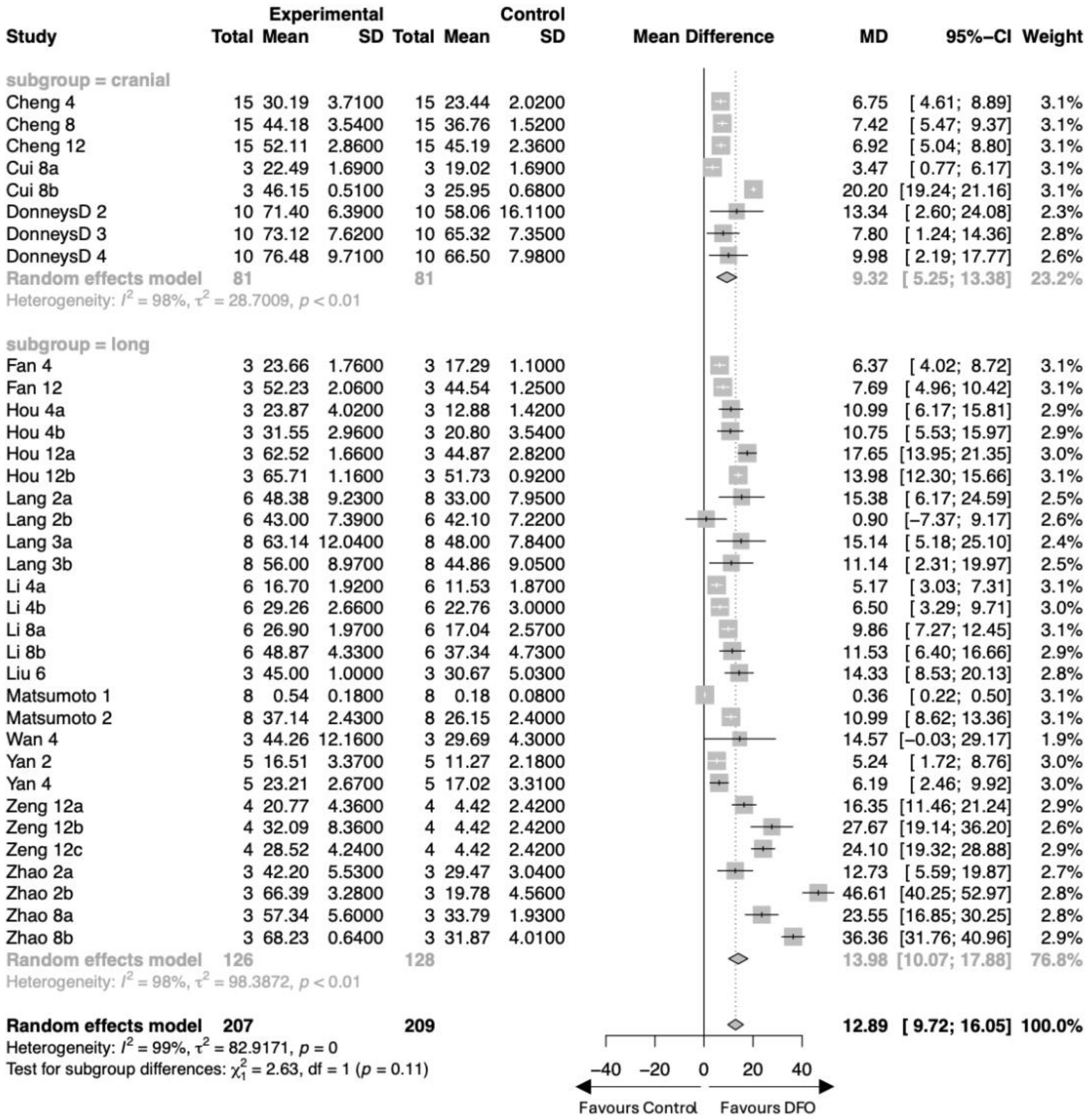


*Figure S2.2: Forest plot of subgroup analysis for bone type, without disease models*

***Figure S3.1 and S3.2:*** Application route

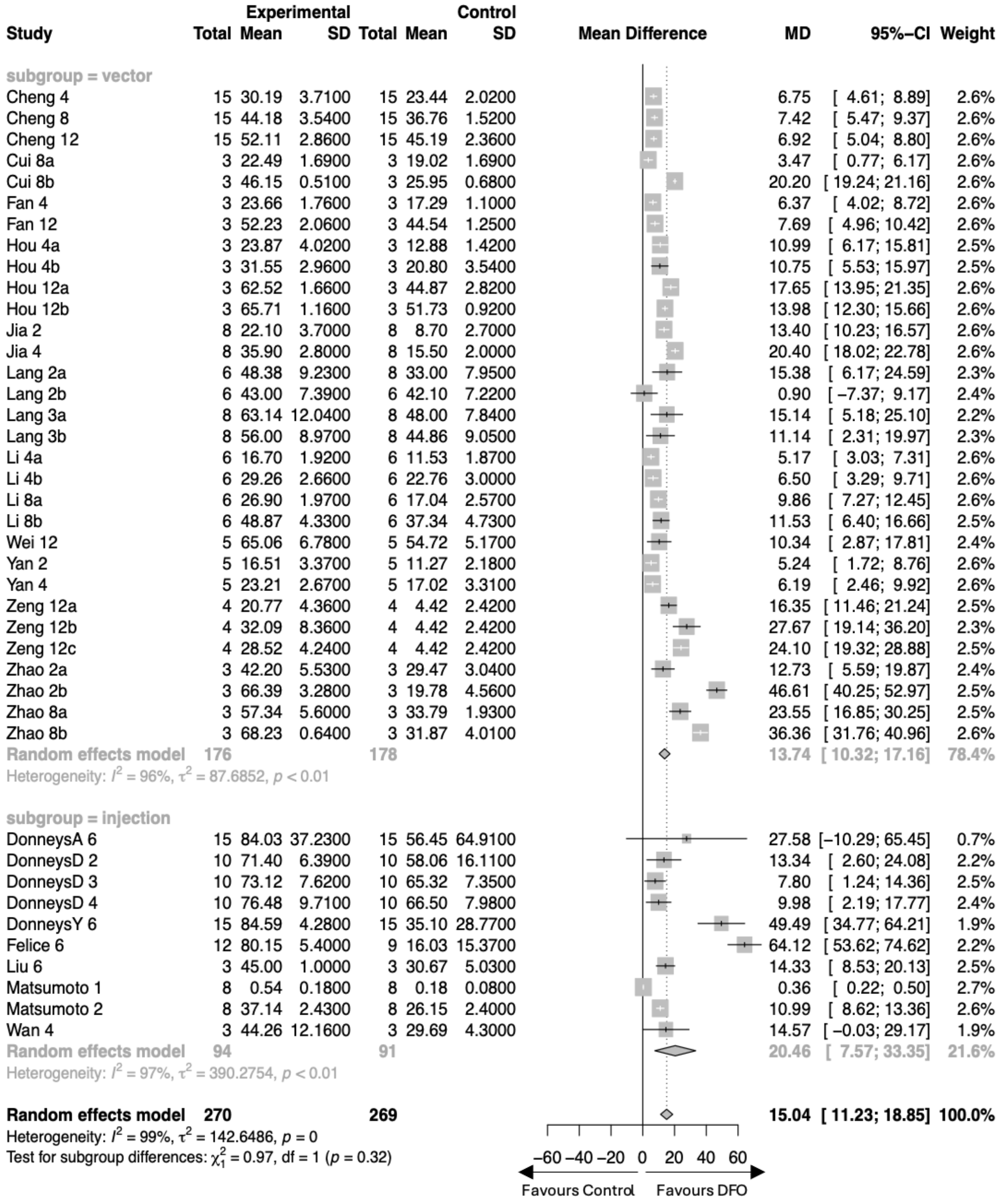


***Figure S3.1: Forest plot for analyzing the application route subgroups of direct injections and vector systems, including disease models***

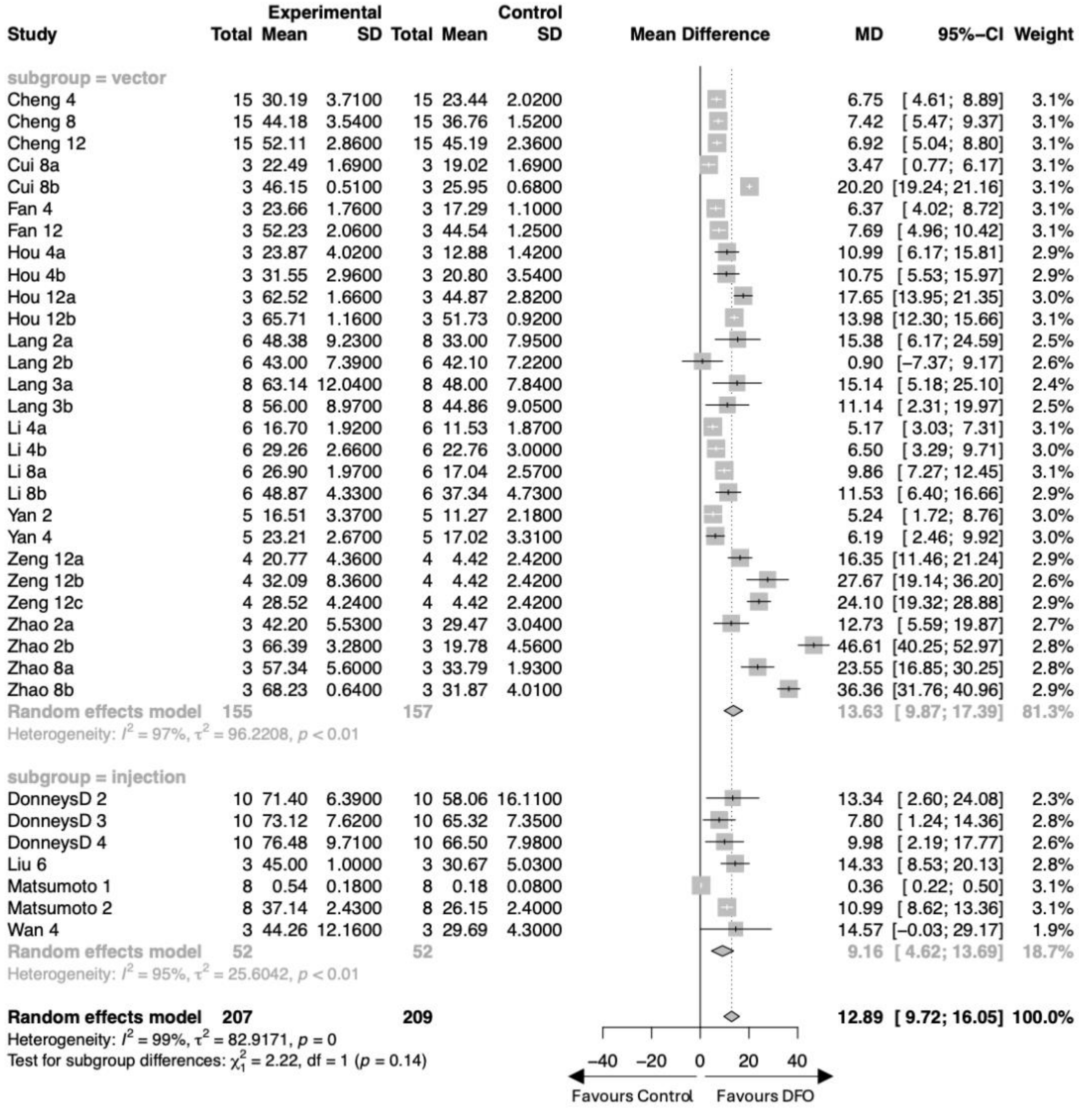


***Figure S3.2: Forest plot of subgroup analysis for application route, without disease models***

***Figure S4.1 and S4.2:*** Type of fracture defect model

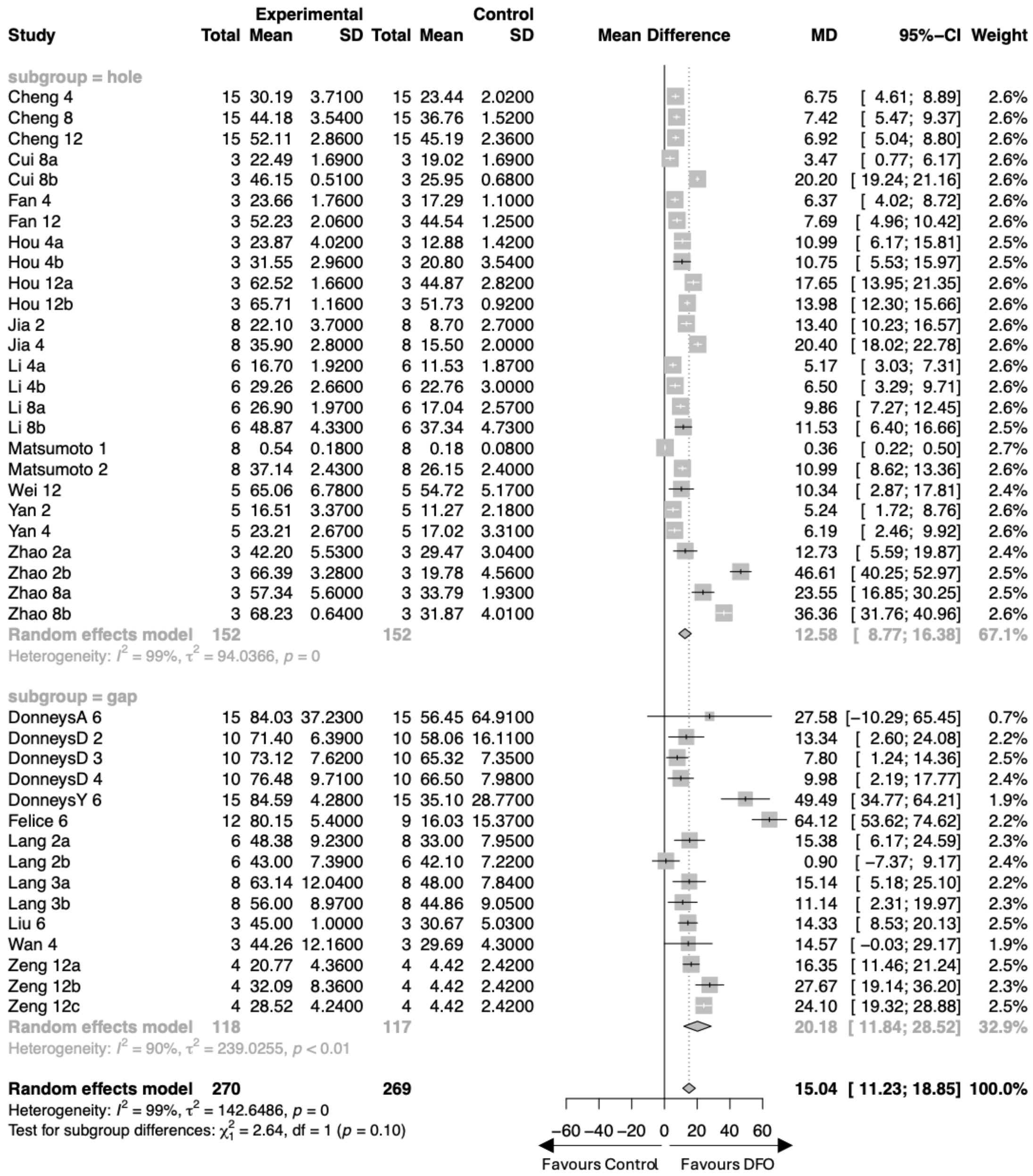


***Figure S4.1: Forest plot for analyzing the fracture defect model subgroups of drill hole and fracture gap, including disease models***

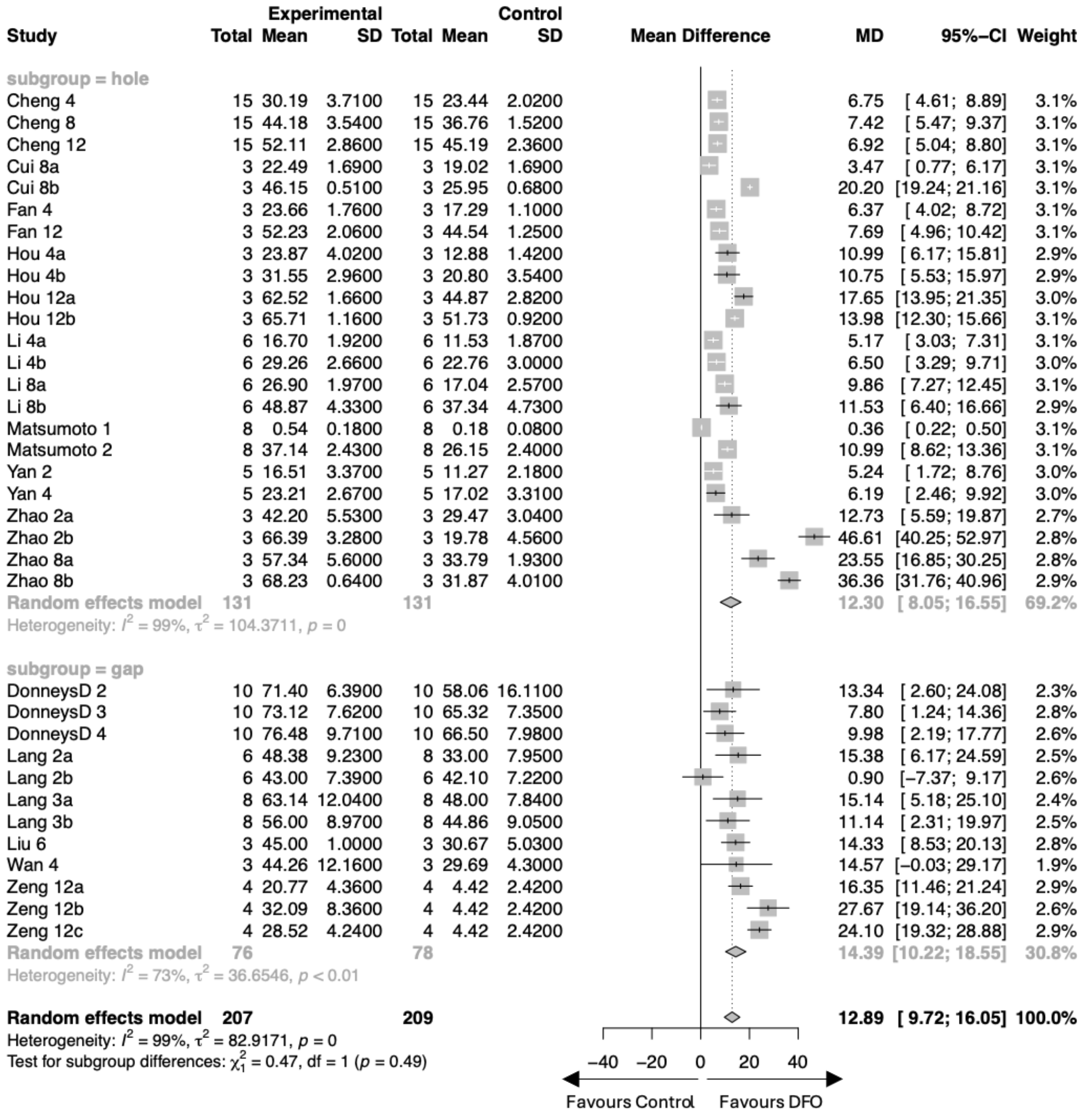


***Figure S4.2: Forest plot of subgroup analysis for fracture defect model types, without disease models***

***Figure S5.1 and S5.2:*** Time points

| Study | Experimental Total | Experimental Mean | Experimental SD | Control Total | Control Mean | Control SD | MD | 95%-CI | Weight |
|---|---|---|---|---|---|---|---|---|---|
| week = 1 | | | | | | | | | |
| Matsumoto 1 | 8 | 0.54 | 0.1800 | 8 | 0.18 | 0.0800 | 0.36 | [ 0.22; 0.50] | 2.7% |
| week = 2 | | | | | | | | | |
| DonneysD 2 | 10 | 71.40 | 6.3900 | 10 | 58.06 | 16.1100 | 13.34 | [ 2.60; 24.08] | 2.2% |
| Jia 2 | 8 | 22.10 | 3.7000 | 8 | 8.70 | 2.7000 | 13.40 | [10.23; 16.57] | 2.6% |
| Lang 2a | 6 | 48.38 | 9.2300 | 8 | 33.00 | 7.9500 | 15.38 | [ 6.17; 24.59] | 2.3% |
| Lang 2b | 6 | 43.00 | 7.3900 | 6 | 42.10 | 7.2200 | 0.90 | [−7.37; 9.17] | 2.4% |
| Matsumoto 2 | 8 | 37.14 | 2.4300 | 8 | 26.15 | 2.4000 | 10.99 | [ 8.62; 13.36] | 2.6% |
| Yan 2 | 5 | 16.51 | 3.3700 | 5 | 11.27 | 2.1800 | 5.24 | [ 1.72; 8.76] | 2.6% |
| Zhao 2a | 3 | 42.20 | 5.5300 | 3 | 29.47 | 3.0400 | 12.73 | [ 5.59; 19.87] | 2.4% |
| Zhao 2b | 3 | 66.39 | 3.2800 | 3 | 19.78 | 4.5600 | 46.61 | [40.25; 52.97] | 2.5% |
| Random effects model | 49 | | | 51 | | | 14.83 | [ 5.25; 24.40] | 19.6% |
| Heterogeneity: $I^2 = 95\%$, $\tau^2 = 178.9260$, $p < 0.01$ | | | | | | | | | |
| week = 3 | | | | | | | | | |
| DonneysD 3 | 10 | 73.12 | 7.6200 | 10 | 65.32 | 7.3500 | 7.80 | [ 1.24; 14.36] | 2.5% |
| Lang 3a | 8 | 63.14 | 12.0400 | 8 | 48.00 | 7.8400 | 15.14 | [ 5.18; 25.10] | 2.2% |
| Lang 3b | 8 | 56.00 | 8.9700 | 8 | 44.86 | 9.0500 | 11.14 | [ 2.31; 19.97] | 2.3% |
| Random effects model | 26 | | | 26 | | | 10.33 | [ 5.68; 14.99] | 7.0% |
| Heterogeneity: $I^2 = 0\%$, $\tau^2 = 0$, $p = 0.47$ | | | | | | | | | |
| week = 4 | | | | | | | | | |
| Cheng 4 | 15 | 30.19 | 3.7100 | 15 | 23.44 | 2.0200 | 6.75 | [ 4.61; 8.89] | 2.6% |
| DonneysD 4 | 10 | 76.48 | 9.7100 | 10 | 66.50 | 7.9800 | 9.98 | [ 2.19; 17.77] | 2.4% |
| Fan 4 | 3 | 23.66 | 1.7600 | 3 | 17.29 | 1.1000 | 6.37 | [ 4.02; 8.72] | 2.6% |
| Hou 4a | 3 | 23.87 | 4.0200 | 3 | 12.88 | 1.4200 | 10.99 | [ 6.17; 15.81] | 2.5% |
| Hou 4b | 3 | 31.55 | 2.9600 | 3 | 20.80 | 3.5400 | 10.75 | [ 5.53; 15.97] | 2.5% |
| Jia 4 | 8 | 35.90 | 2.8000 | 8 | 15.50 | 2.0000 | 20.40 | [18.02; 22.78] | 2.6% |
| Li 4a | 6 | 16.70 | 1.9200 | 6 | 11.53 | 1.8700 | 5.17 | [ 3.03; 7.31] | 2.6% |
| Li 4b | 6 | 29.26 | 2.6600 | 6 | 22.76 | 3.0000 | 6.50 | [ 3.29; 9.71] | 2.6% |
| Wan 4 | 3 | 44.26 | 12.1600 | 3 | 29.69 | 4.3000 | 14.57 | [−0.03; 29.17] | 1.9% |
| Yan 4 | 5 | 23.21 | 2.6700 | 5 | 17.02 | 3.3100 | 6.19 | [ 2.46; 9.92] | 2.6% |
| Random effects model | 62 | | | 62 | | | 9.36 | [ 6.09; 12.62] | 25.1% |
| Heterogeneity: $I^2 = 92\%$, $\tau^2 = 21.9862$, $p < 0.01$ | | | | | | | | | |
| week = 6 | | | | | | | | | |
| DonneysA 6 | 15 | 84.03 | 37.2300 | 15 | 56.45 | 64.9100 | 27.58 | [−10.29; 65.45] | 0.7% |
| DonneysY 6 | 15 | 84.59 | 4.2800 | 15 | 35.10 | 28.7700 | 49.49 | [34.77; 64.21] | 1.9% |
| Felice 6 | 12 | 80.15 | 5.4000 | 9 | 16.03 | 15.3700 | 64.12 | [53.62; 74.62] | 2.2% |
| Liu 6 | 3 | 45.00 | 1.0000 | 3 | 30.67 | 5.0300 | 14.33 | [ 8.53; 20.13] | 2.5% |
| Random effects model | 45 | | | 42 | | | 39.70 | [15.76; 63.64] | 7.3% |
| Heterogeneity: $I^2 = 96\%$, $\tau^2 = 508.2263$, $p < 0.01$ | | | | | | | | | |
| week = 8 | | | | | | | | | |
| Cheng 8 | 15 | 44.18 | 3.5400 | 15 | 36.76 | 1.5200 | 7.42 | [ 5.47; 9.37] | 2.6% |
| Cui 8a | 3 | 22.49 | 1.6900 | 3 | 19.02 | 1.6900 | 3.47 | [ 0.77; 6.17] | 2.6% |
| Cui 8b | 3 | 46.15 | 0.5100 | 3 | 25.95 | 0.6800 | 20.20 | [19.24; 21.16] | 2.6% |
| Li 8a | 6 | 26.90 | 1.9700 | 6 | 17.04 | 2.5700 | 9.86 | [ 7.27; 12.45] | 2.6% |
| Li 8b | 6 | 48.87 | 4.3300 | 6 | 37.34 | 4.7300 | 11.53 | [ 6.40; 16.66] | 2.5% |
| Zhao 8a | 3 | 57.34 | 5.6000 | 3 | 33.79 | 1.9300 | 23.55 | [16.85; 30.25] | 2.5% |
| Zhao 8b | 3 | 68.23 | 0.6400 | 3 | 31.87 | 4.0100 | 36.36 | [31.76; 40.96] | 2.6% |
| Random effects model | 39 | | | 39 | | | 15.92 | [ 7.51; 24.32] | 18.1% |
| Heterogeneity: $I^2 = 98\%$, $\tau^2 = 124.6950$, $p < 0.01$ | | | | | | | | | |
| week = 12 | | | | | | | | | |
| Cheng 12 | 15 | 52.11 | 2.8600 | 15 | 45.19 | 2.3600 | 6.92 | [ 5.04; 8.80] | 2.6% |
| Fan 12 | 3 | 52.23 | 2.0600 | 3 | 44.54 | 1.2500 | 7.69 | [ 4.96; 10.42] | 2.6% |
| Hou 12a | 3 | 62.52 | 1.6600 | 3 | 44.87 | 2.8200 | 17.65 | [13.95; 21.35] | 2.6% |
| Hou 12b | 3 | 65.71 | 1.1600 | 3 | 51.73 | 0.9200 | 13.98 | [12.30; 15.66] | 2.6% |
| Wei 12 | 5 | 65.06 | 6.7800 | 5 | 54.72 | 5.1700 | 10.34 | [ 2.87; 17.81] | 2.4% |
| Zeng 12a | 4 | 20.77 | 4.3600 | 4 | 4.42 | 2.4200 | 16.35 | [11.46; 21.24] | 2.5% |
| Zeng 12b | 4 | 32.09 | 8.3600 | 4 | 4.42 | 2.4200 | 27.67 | [19.14; 36.20] | 2.3% |
| Zeng 12c | 4 | 28.52 | 4.2400 | 4 | 4.42 | 2.4200 | 24.10 | [19.32; 28.88] | 2.5% |
| Random effects model | 41 | | | 41 | | | 15.16 | [10.22; 20.10] | 20.3% |
| Heterogeneity: $I^2 = 92\%$, $\tau^2 = 44.8977$, $p < 0.01$ | | | | | | | | | |
| **Random effects model** | **270** | | | **269** | | | **15.04** | **[11.23; 18.85]** | **100.0%** |
| Heterogeneity: $I^2 = 99\%$, $\tau^2 = 142.6486$, $p = 0$ | | | | | | | | | |
| Test for subgroup differences: $\chi^2_6 = 113.22$, df = 6 ($p < 0.01$) | | | | | | | | | |

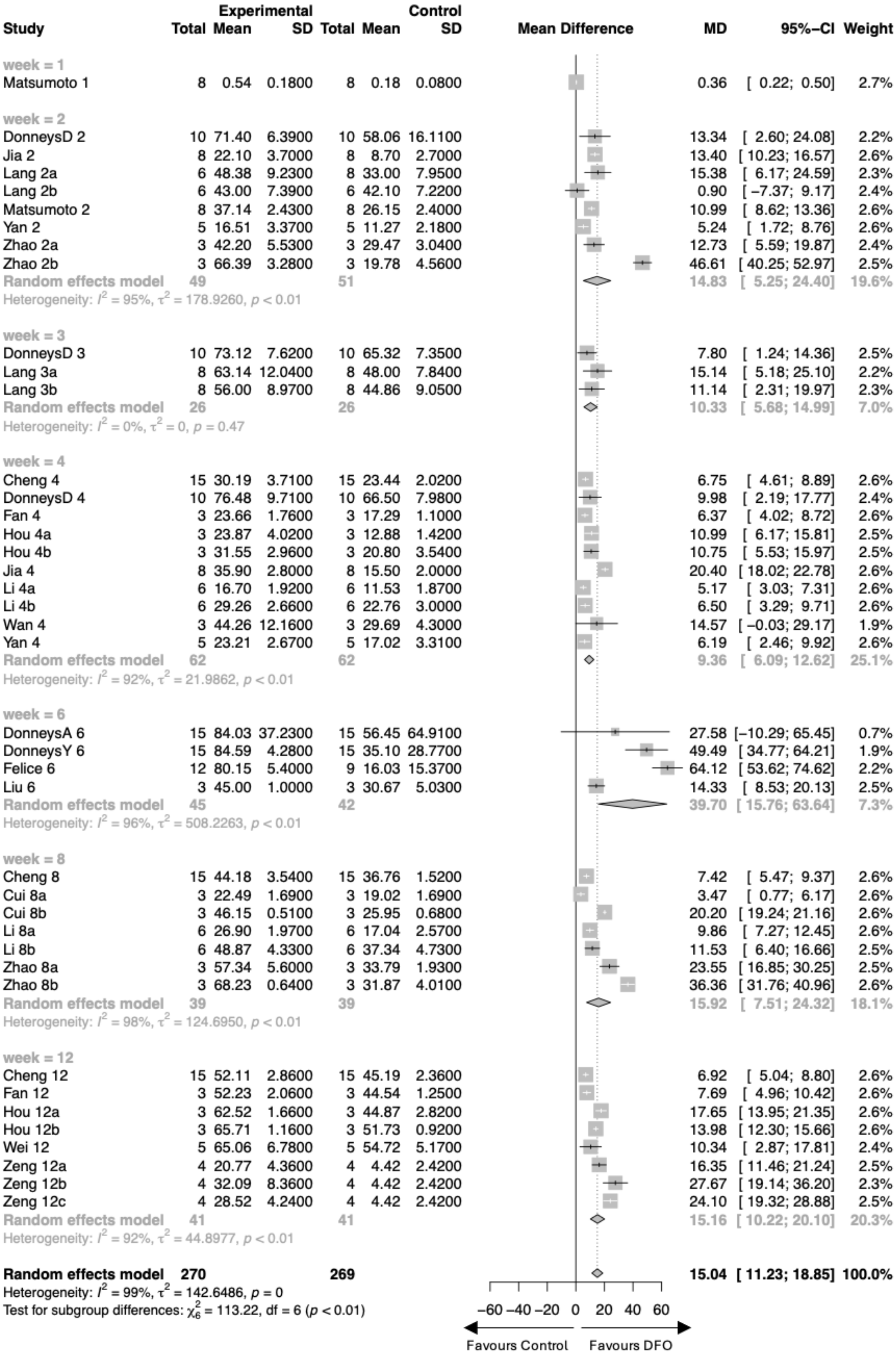


***Figure S5.1: Forest plot for analyzing the subgroups of different time points of the measurements after surgery (in weeks), including disease models***

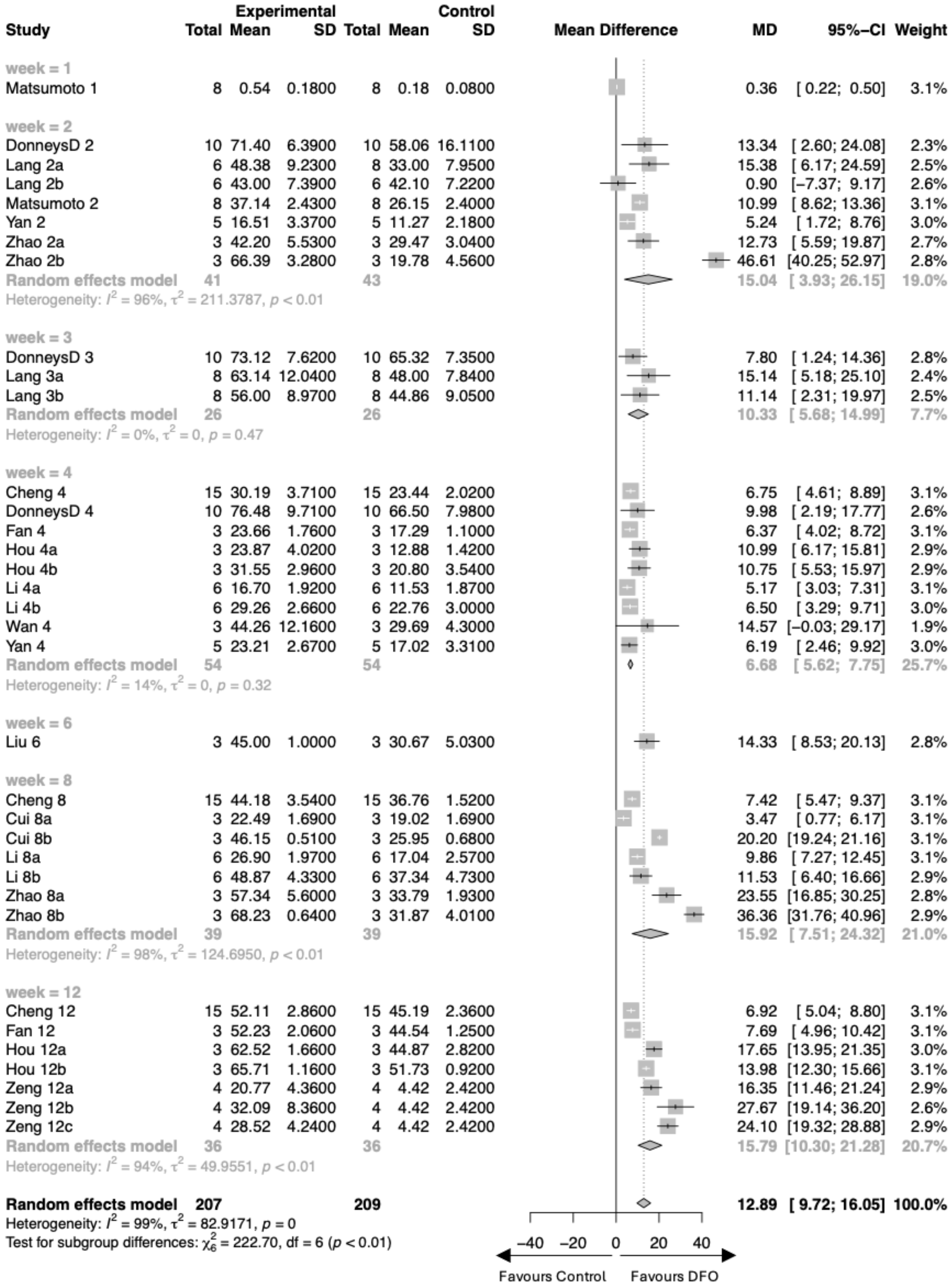


***Figure S5.2: Forest plot of subgroup analysis for the time points of measurements, without disease models***

***Figure S6.1 and S6.2:*** Sex

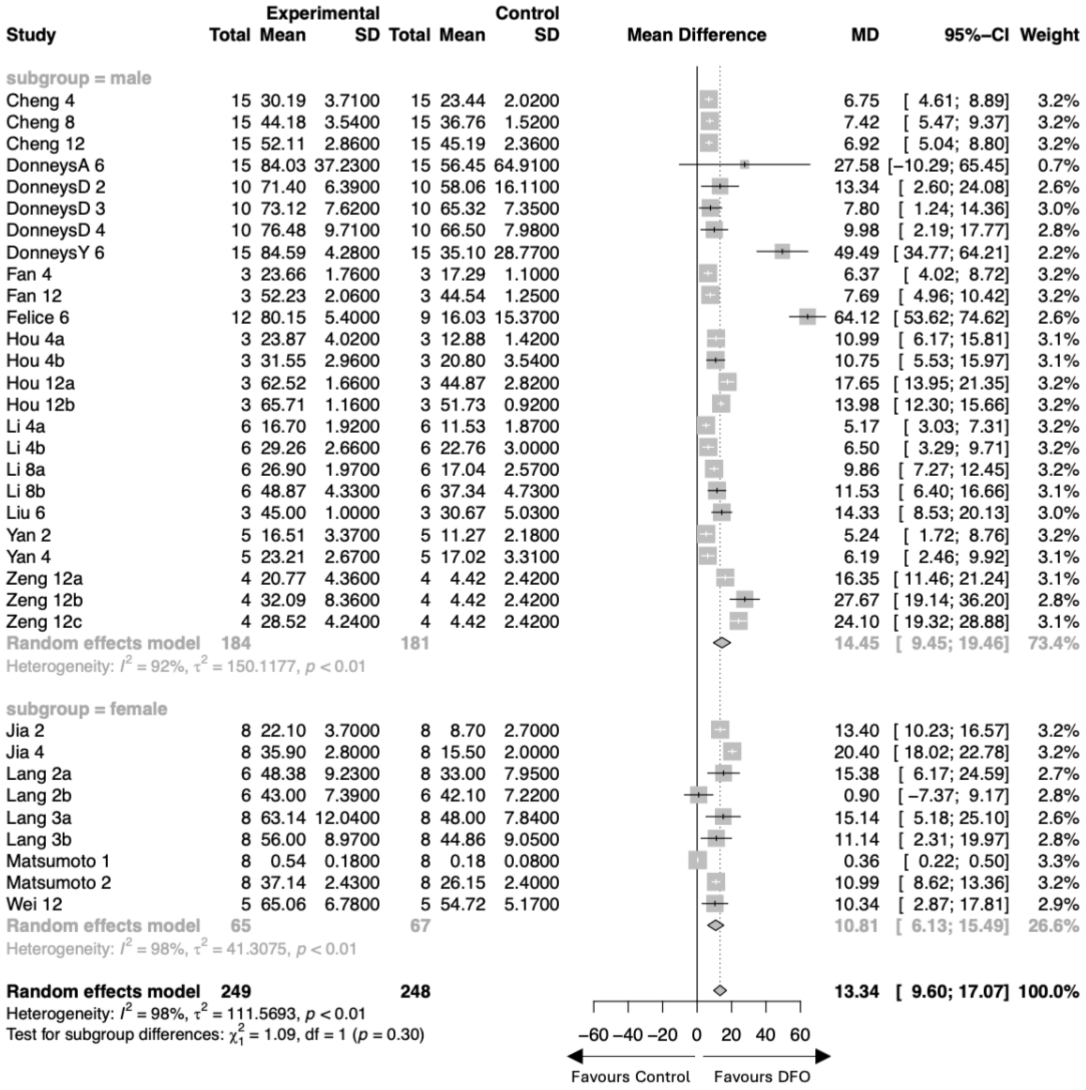


***Figure S6.1: Forest plot for analyzing the impact of the sex of the subjects on the results, including disease models***

| Study | Experimental Total | Experimental Mean | Experimental SD | Control Total | Control Mean | Control SD | MD | 95%-CI | Weight |
|---|---|---|---|---|---|---|---|---|---|
| **subgroup = male** | | | | | | | | | |
| Cheng 4 | 15 | 30.19 | 3.7100 | 15 | 23.44 | 2.0200 | 6.75 | [ 4.61; 8.89] | 4.2% |
| Cheng 8 | 15 | 44.18 | 3.5400 | 15 | 36.76 | 1.5200 | 7.42 | [ 5.47; 9.37] | 4.3% |
| Cheng 12 | 15 | 52.11 | 2.8600 | 15 | 45.19 | 2.3600 | 6.92 | [ 5.04; 8.80] | 4.3% |
| DonneysD 2 | 10 | 71.40 | 6.3900 | 10 | 58.06 | 16.1100 | 13.34 | [ 2.60; 24.08] | 2.1% |
| DonneysD 3 | 10 | 73.12 | 7.6200 | 10 | 65.32 | 7.3500 | 7.80 | [ 1.24; 14.36] | 3.1% |
| DonneysD 4 | 10 | 76.48 | 9.7100 | 10 | 66.50 | 7.9800 | 9.98 | [ 2.19; 17.77] | 2.8% |
| Fan 4 | 3 | 23.66 | 1.7600 | 3 | 17.29 | 1.1000 | 6.37 | [ 4.02; 8.72] | 4.2% |
| Fan 12 | 3 | 52.23 | 2.0600 | 3 | 44.54 | 1.2500 | 7.69 | [ 4.96; 10.42] | 4.1% |
| Hou 4a | 3 | 23.87 | 4.0200 | 3 | 12.88 | 1.4200 | 10.99 | [ 6.17; 15.81] | 3.6% |
| Hou 4b | 3 | 31.55 | 2.9600 | 3 | 20.80 | 3.5400 | 10.75 | [ 5.53; 15.97] | 3.5% |
| Hou 12a | 3 | 62.52 | 1.6600 | 3 | 44.87 | 2.8200 | 17.65 | [13.95; 21.35] | 3.9% |
| Hou 12b | 3 | 65.71 | 1.1600 | 3 | 51.73 | 0.9200 | 13.98 | [12.30; 15.66] | 4.3% |
| Li 4a | 6 | 16.70 | 1.9200 | 6 | 11.53 | 1.8700 | 5.17 | [ 3.03; 7.31] | 4.2% |
| Li 4b | 6 | 29.26 | 2.6600 | 6 | 22.76 | 3.0000 | 6.50 | [ 3.29; 9.71] | 4.0% |
| Li 8a | 6 | 26.90 | 1.9700 | 6 | 17.04 | 2.5700 | 9.86 | [ 7.27; 12.45] | 4.2% |
| Li 8b | 6 | 48.87 | 4.3300 | 6 | 37.34 | 4.7300 | 11.53 | [ 6.40; 16.66] | 3.5% |
| Liu 6 | 3 | 45.00 | 1.0000 | 3 | 30.67 | 5.0300 | 14.33 | [ 8.53; 20.13] | 3.3% |
| Yan 2 | 5 | 16.51 | 3.3700 | 5 | 11.27 | 2.1800 | 5.24 | [ 1.72; 8.76] | 4.0% |
| Yan 4 | 5 | 23.21 | 2.6700 | 5 | 17.02 | 3.3100 | 6.19 | [ 2.46; 9.92] | 3.9% |
| Zeng 12a | 4 | 20.77 | 4.3600 | 4 | 4.42 | 2.4200 | 16.35 | [11.46; 21.24] | 3.6% |
| Zeng 12b | 4 | 32.09 | 8.3600 | 4 | 4.42 | 2.4200 | 27.67 | [19.14; 36.20] | 2.6% |
| Zeng 12c | 4 | 28.52 | 4.2400 | 4 | 4.42 | 2.4200 | 24.10 | [19.32; 28.88] | 3.6% |
| **Random effects model** | 142 | | | 142 | | | 10.73 | [ 8.43; 13.03] | 81.5% |
| Heterogeneity: $I^2 = 87\%$, $\tau^2 = 25.1381$, $p < 0.01$ | | | | | | | | | |
| **subgroup = female** | | | | | | | | | |
| Lang 2a | 6 | 48.38 | 9.2300 | 8 | 33.00 | 7.9500 | 15.38 | [ 6.17; 24.59] | 2.4% |
| Lang 2b | 6 | 43.00 | 7.3900 | 6 | 42.10 | 7.2200 | 0.90 | [−7.37; 9.17] | 2.7% |
| Lang 3a | 8 | 63.14 | 12.0400 | 8 | 48.00 | 7.8400 | 15.14 | [ 5.18; 25.10] | 2.3% |
| Lang 3b | 8 | 56.00 | 8.9700 | 8 | 44.86 | 9.0500 | 11.14 | [ 2.31; 19.97] | 2.5% |
| Matsumoto 1 | 8 | 0.54 | 0.1800 | 8 | 0.18 | 0.0800 | 0.36 | [ 0.22; 0.50] | 4.4% |
| Matsumoto 2 | 8 | 37.14 | 2.4300 | 8 | 26.15 | 2.4000 | 10.99 | [ 8.62; 13.36] | 4.2% |
| **Random effects model** | 44 | | | 46 | | | 8.27 | [ 2.72; 13.83] | 18.5% |
| Heterogeneity: $I^2 = 95\%$, $\tau^2 = 35.9183$, $p < 0.01$ | | | | | | | | | |
| **Random effects model** | **186** | | | **188** | | | **10.27** | **[ 8.12; 12.42]** | **100.0%** |
| Heterogeneity: $I^2 = 97\%$, $\tau^2 = 27.2586$, $p < 0.01$ | | | | | | | | | |
| Test for subgroup differences: $\chi^2_1 = 0.64$, df = 1 ($p = 0.42$) | | | | | | | | | |

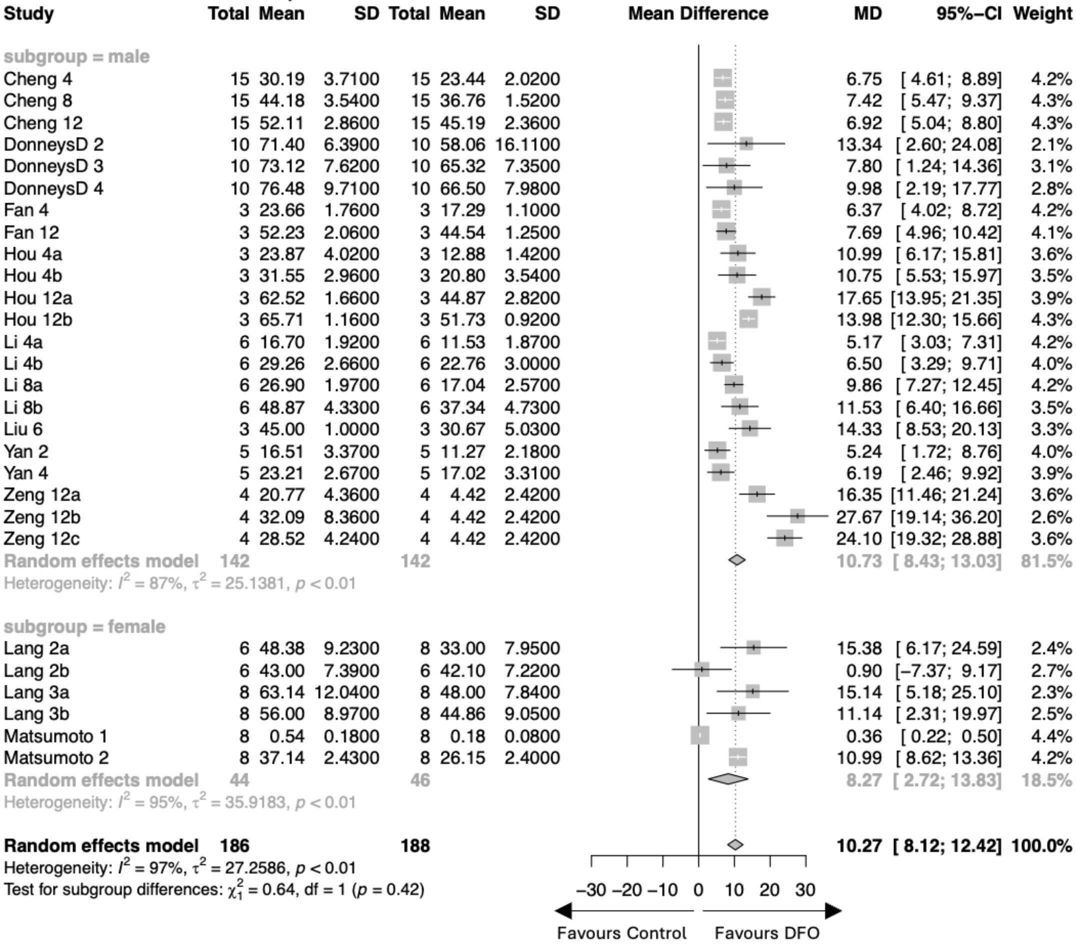


***Figure S6.2: Forest plot for analyzing the impact of the sex of the subjects on the results, without disease models***

***Figure S7.1 and S7.2:*** Age

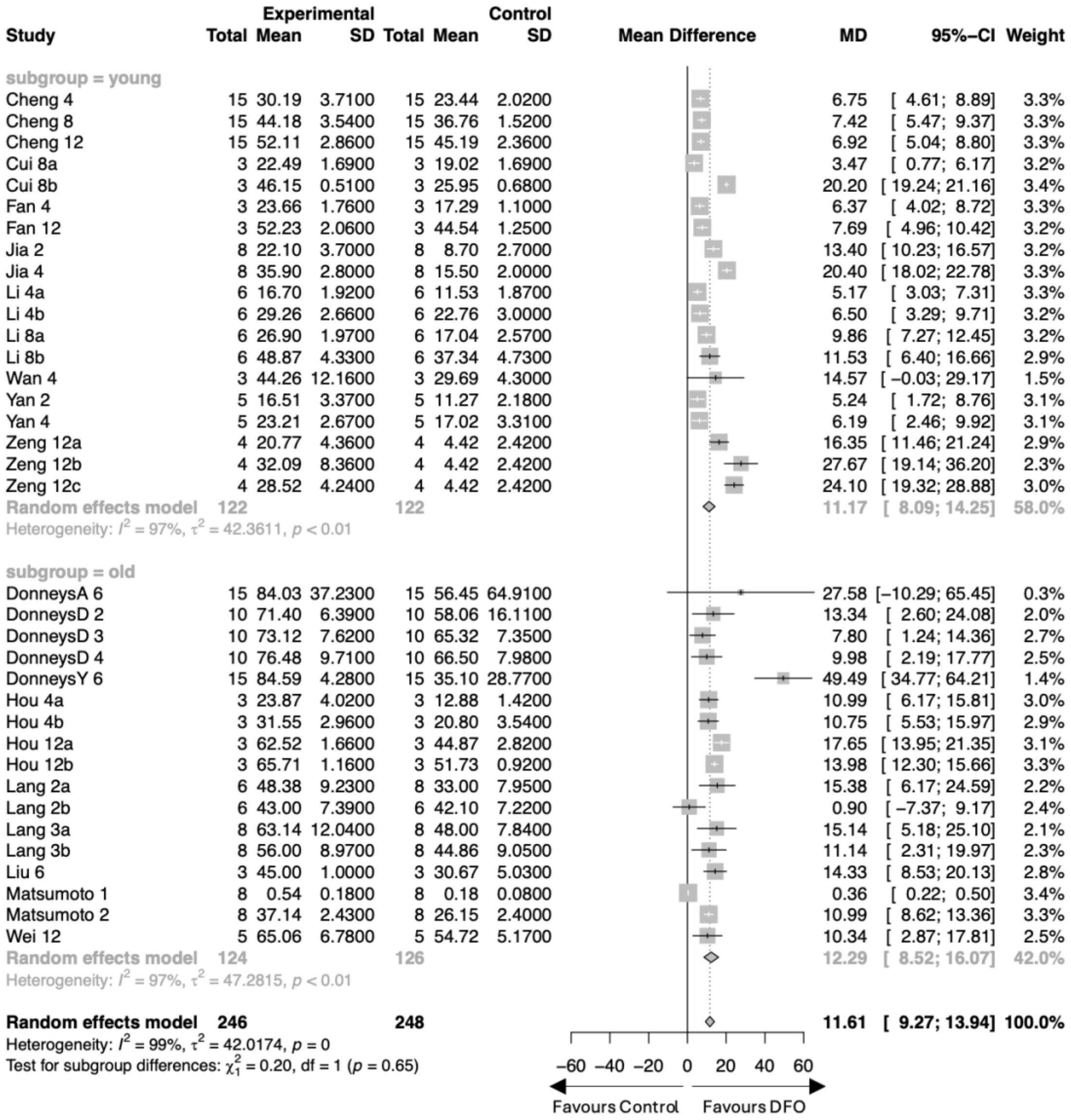


***Figure S7.1: Forest plot for analyzing the impact of the age of the subjects on the results, including disease models***

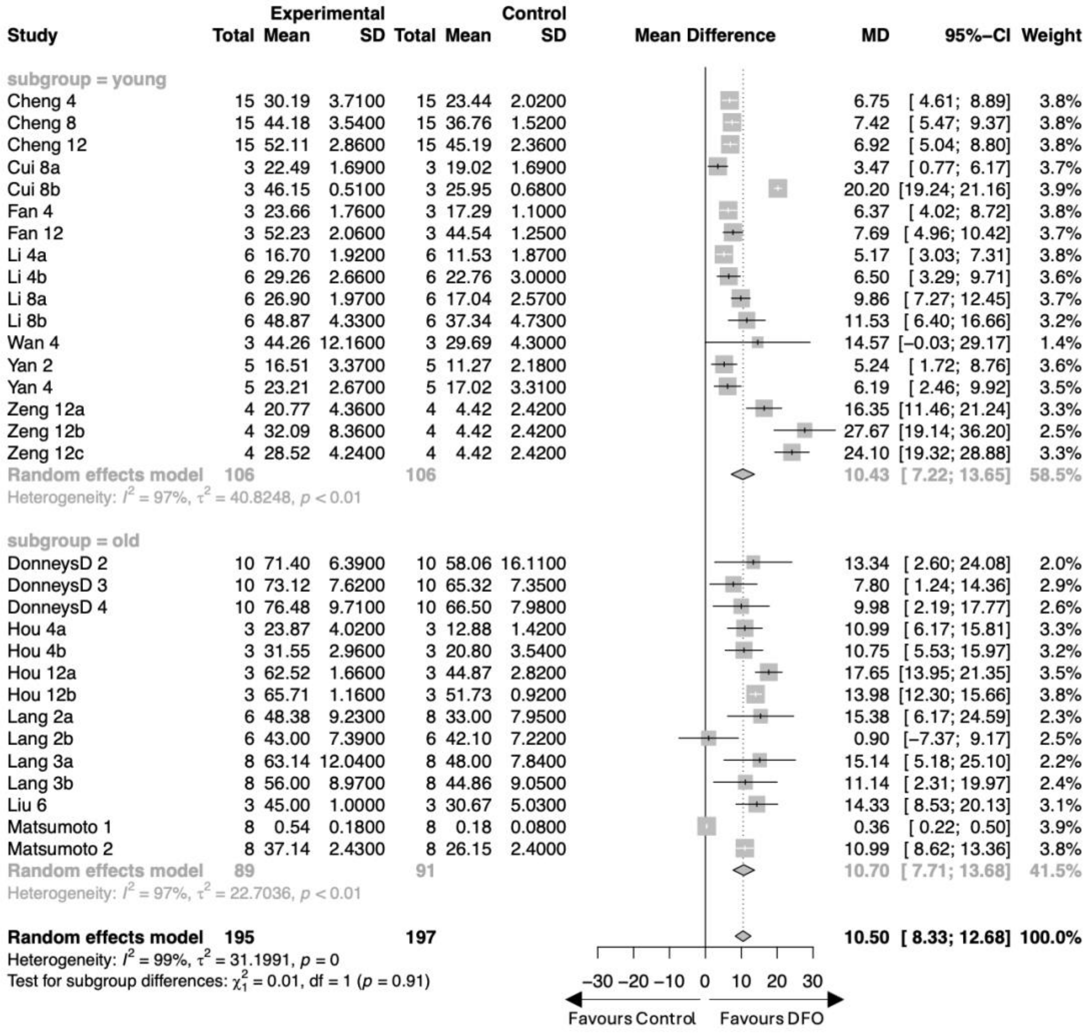


***Figure S7.2: Forest plot for analyzing the impact of the age of the subjects on the results, without disease models***

## Secondary Endpoints

***Figure S8:*** Bone Volume (BV)

Over half of the study groups showed a significant BV increase compared to controls. The aggregate outcome, represented by the grey diamond in the meta-analysis, indicates a significant improvement with an MD of 2.4 mm³ (CI: 0,49; 4.32).

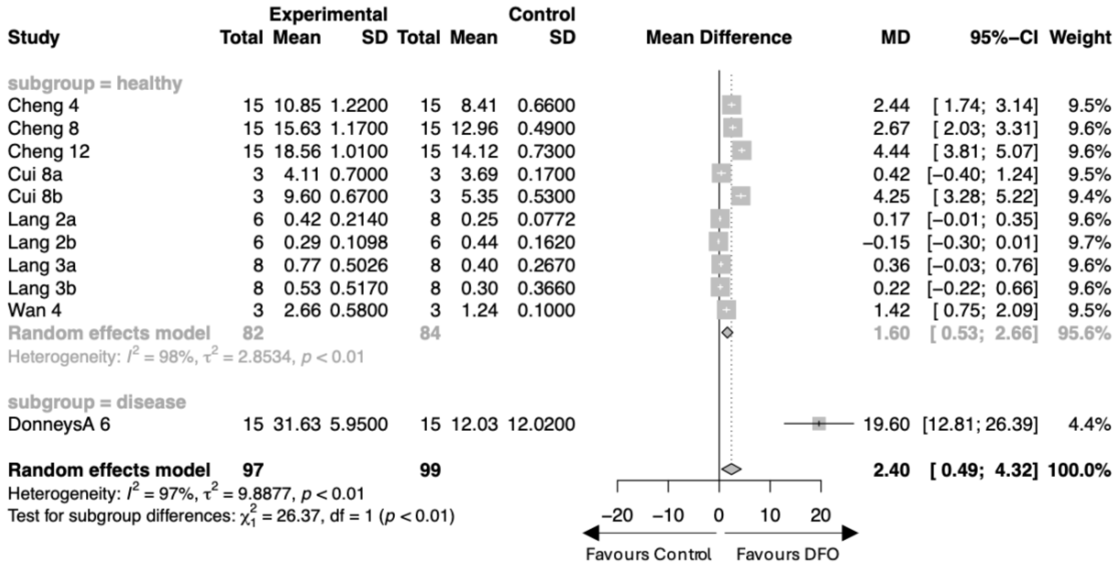


***Forest plot for analyzing the outcome parameter BV***

***Figure S9:*** Tissue Volume (TV)

Regarding TV, the pooled effect size, depicted by the grey diamond, indicated a slight increase in callus or tissue volume by 0.75 mm³ (CI: -0.19; 1.69) due to DFO treatment, though this was not statistically significant, as the CI crosses the line of no effect.

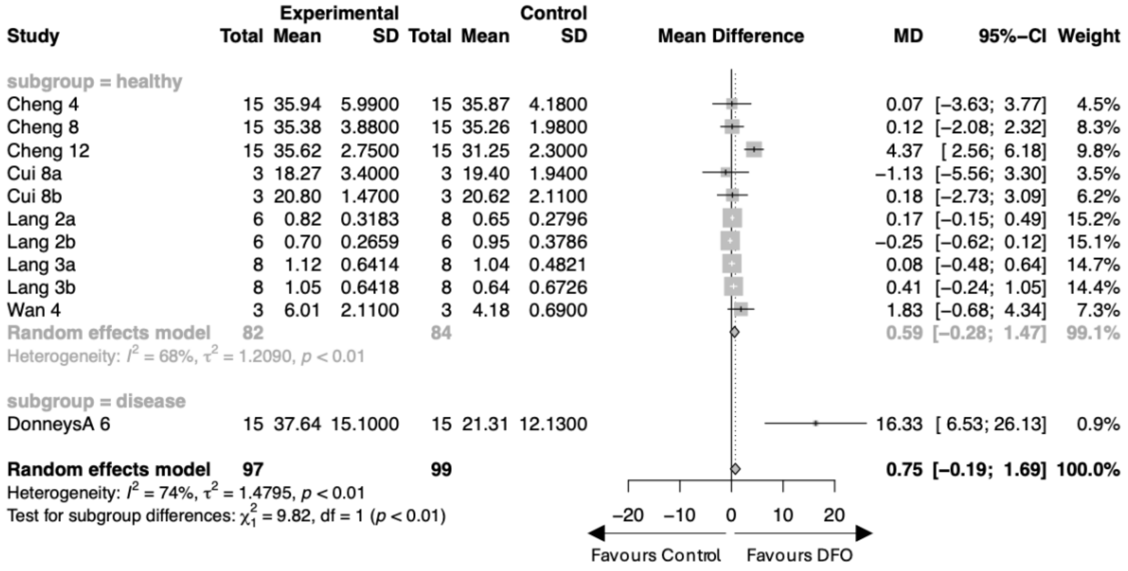


***Forest plot for analyzing the outcome parameter TV***

***Figure S10:*** Bone Mineral Density (BMD)

The analysis showed a significant increase in BMD after DFO treatment, with an MD of 105.67 mg/cm³ (CI: 69.35; 141.99). This suggests that DFO enhances bone mineralization, likely due to improved nutrient delivery from enhanced vascularization.

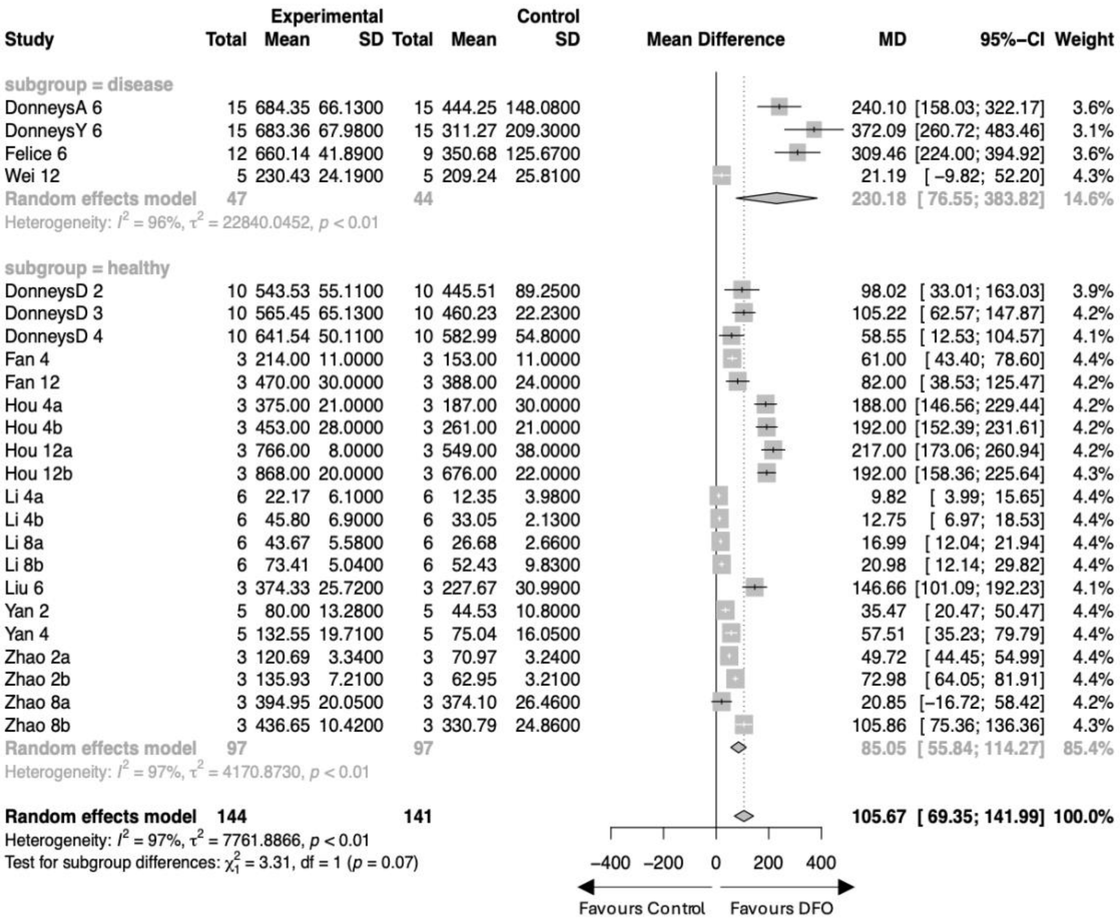


***Forest plot for analyzing the outcome parameter BMD***

*Figure S11:* Trabecular Separation or Spacing (Tb.Sp.)

In Tb.Sp., a smaller distance between the trabeculae indicates denser, better-quality bone. Thus, a decrease in Tb.Sp. favors DFO, shown to the left in the forest plot. The analysis revealed a significant reduction in Tb.Sp. in all but two studies (Li 8a, Wei 12) [27,33], with a pooled MD of -0.21 mm (CI: -0.27; -0.14), indicating DFO improves bone structure and density.

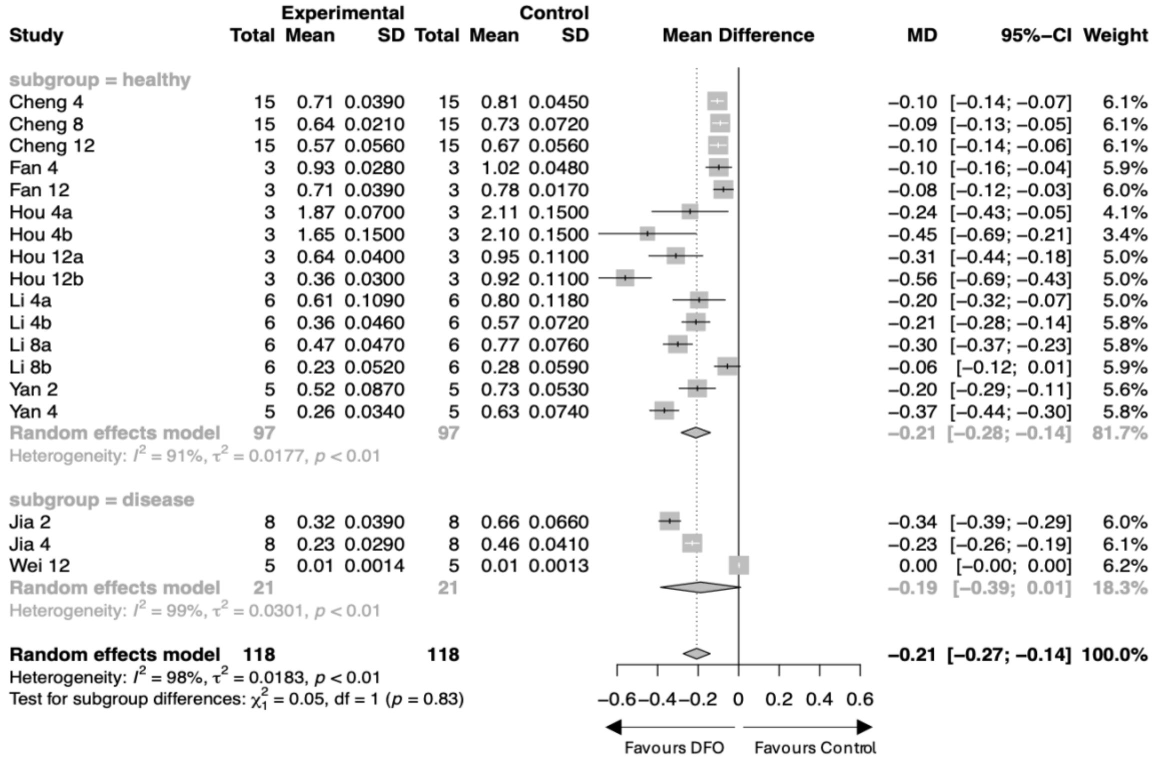


***Forest plot for analyzing the outcome parameter Tb.Sp.***

***Figure S12:*** Trabecular Thickness (Tb.Th.)
In Tb.Th., larger values indicate thicker, stronger trabeculae, favoring DFO on the right side of the forest plot. The analysis shows a significant pooled MD of 0.04 mm (CI: 0.02; 0.07) in favor of DFO.

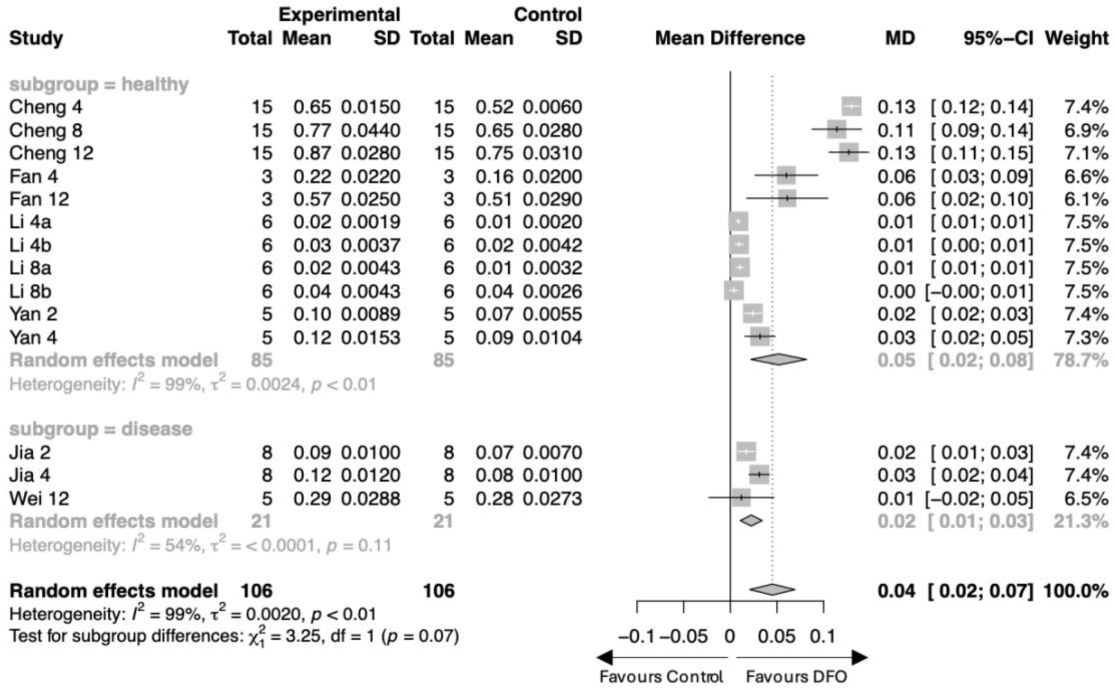


***Forest plot for analyzing the outcome parameter Tb.Th.***

## Sensitivity Analysis

***Figure S13:*** Sensitivity analysis using the leave-one-out method

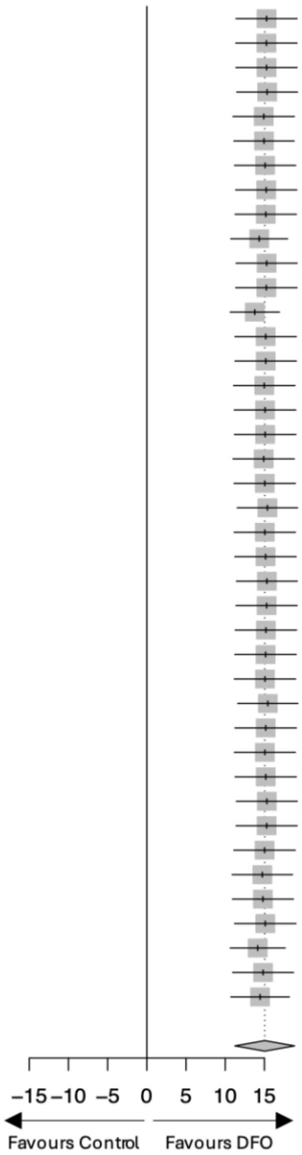


| Study | MD | 95%−CI | P−value | Tau2 | Tau | I2 |
|---|---|---|---|---|---|---|
| Omitting Cheng 4 | 15.27 | [11.37; 19.17] | < 0.01 | 145.2935 | 12.0538 | 99% |
| Omitting Cheng 8 | 15.25 | [11.35; 19.15] | < 0.01 | 145.6356 | 12.0680 | 99% |
| Omitting Cheng 12 | 15.27 | [11.37; 19.16] | < 0.01 | 145.3835 | 12.0575 | 99% |
| Omitting Cui 8a | 15.35 | [11.48; 19.22] | < 0.01 | 143.2039 | 11.9668 | 99% |
| Omitting Cui 8b | 14.91 | [11.00; 18.82] | < 0.01 | 146.3553 | 12.0977 | 98% |
| Omitting DonneysA 6 | 14.95 | [11.11; 18.79] | < 0.01 | 143.4816 | 11.9784 | 99% |
| Omitting DonneysD 2 | 15.09 | [11.19; 18.99] | < 0.01 | 146.5792 | 12.1070 | 99% |
| Omitting DonneysD 3 | 15.23 | [11.33; 19.13] | < 0.01 | 145.7256 | 12.0717 | 99% |
| Omitting DonneysD 4 | 15.17 | [11.27; 19.08] | < 0.01 | 146.3721 | 12.0984 | 99% |
| Omitting DonneysY 6 | 14.33 | [10.71; 17.96] | < 0.01 | 125.5640 | 11.2055 | 99% |
| Omitting Fan 4 | 15.28 | [11.39; 19.17] | < 0.01 | 145.0871 | 12.0452 | 99% |
| Omitting Fan 12 | 15.25 | [11.34; 19.15] | < 0.01 | 145.7573 | 12.0730 | 99% |
| Omitting Felice 6 | 13.78 | [10.64; 16.91] | < 0.01 | 91.4583 | 9.5634 | 99% |
| Omitting Hou 4a | 15.16 | [11.24; 19.07] | < 0.01 | 146.8207 | 12.1170 | 99% |
| Omitting Hou 4b | 15.16 | [11.25; 19.07] | < 0.01 | 146.7405 | 12.1136 | 99% |
| Omitting Hou 12a | 14.98 | [11.07; 18.90] | < 0.01 | 147.0114 | 12.1248 | 99% |
| Omitting Hou 12b | 15.08 | [11.16; 19.00] | < 0.01 | 147.3849 | 12.1402 | 99% |
| Omitting Jia 2 | 15.10 | [11.18; 19.02] | < 0.01 | 147.2899 | 12.1363 | 99% |
| Omitting Jia 4 | 14.91 | [11.00; 18.81] | < 0.01 | 146.2560 | 12.0936 | 99% |
| Omitting Lang 2a | 15.04 | [11.14; 18.95] | < 0.01 | 146.7828 | 12.1154 | 99% |
| Omitting Lang 2b | 15.38 | [11.53; 19.23] | < 0.01 | 141.8590 | 11.9105 | 99% |
| Omitting Lang 3a | 15.05 | [11.14; 18.96] | < 0.01 | 146.7021 | 12.1121 | 99% |
| Omitting Lang 3b | 15.14 | [11.24; 19.05] | < 0.01 | 146.5355 | 12.1052 | 99% |
| Omitting Li 4a | 15.31 | [11.43; 19.20] | < 0.01 | 144.3687 | 12.0154 | 99% |
| Omitting Li 4b | 15.27 | [11.38; 19.17] | < 0.01 | 145.1597 | 12.0482 | 99% |
| Omitting Li 8a | 15.19 | [11.28; 19.10] | < 0.01 | 146.6077 | 12.1082 | 99% |
| Omitting Li 8b | 15.14 | [11.23; 19.06] | < 0.01 | 146.9148 | 12.1208 | 99% |
| Omitting Liu 6 | 15.07 | [11.15; 18.99] | < 0.01 | 147.1528 | 12.1307 | 99% |
| Omitting Matsumoto 1 | 15.43 | [11.60; 19.27] | < 0.01 | 140.4091 | 11.8494 | 96% |
| Omitting Matsumoto 2 | 15.16 | [11.24; 19.08] | < 0.01 | 146.9321 | 12.1216 | 99% |
| Omitting Wan 4 | 15.06 | [11.17; 18.95] | < 0.01 | 146.1121 | 12.0877 | 99% |
| Omitting Wei 12 | 15.17 | [11.26; 19.07] | < 0.01 | 146.4851 | 12.1031 | 99% |
| Omitting Yan 2 | 15.31 | [11.42; 19.19] | < 0.01 | 144.4363 | 12.0182 | 99% |
| Omitting Yan 4 | 15.28 | [11.39; 19.17] | < 0.01 | 144.9928 | 12.0413 | 99% |
| Omitting Zeng 12a | 15.02 | [11.10; 18.94] | < 0.01 | 147.1353 | 12.1299 | 99% |
| Omitting Zeng 12b | 14.74 | [10.88; 18.59] | < 0.01 | 142.2872 | 11.9284 | 99% |
| Omitting Zeng 12c | 14.81 | [10.92; 18.69] | < 0.01 | 144.3848 | 12.0160 | 99% |
| Omitting Zhao 2a | 15.11 | [11.20; 19.02] | < 0.01 | 146.9290 | 12.1214 | 99% |
| Omitting Zhao 2b | 14.14 | [10.64; 17.65] | < 0.01 | 115.7344 | 10.7580 | 99% |
| Omitting Zhao 8a | 14.83 | [10.95; 18.72] | < 0.01 | 144.7192 | 12.0299 | 99% |
| Omitting Zhao 8b | 14.44 | [10.72; 18.17] | < 0.01 | 131.8983 | 11.4847 | 99% |
| **Random effects model** | **15.04** | **[11.23; 18.85]** | **< 0.01** | **142.6486** | **11.9436** | **99%** |

***Leave-one-out method****. This figure assesses the influence of individual studies by rerunning the analysis with each study or study arm removed. Each row represents the overall effect that was found when the named study arm was not included. The overall effect remains largely unchanged, indicating no single study is unduly influential.*

***Figure S14:*** *Sensitivity analysis for the influence of low-quality studies*

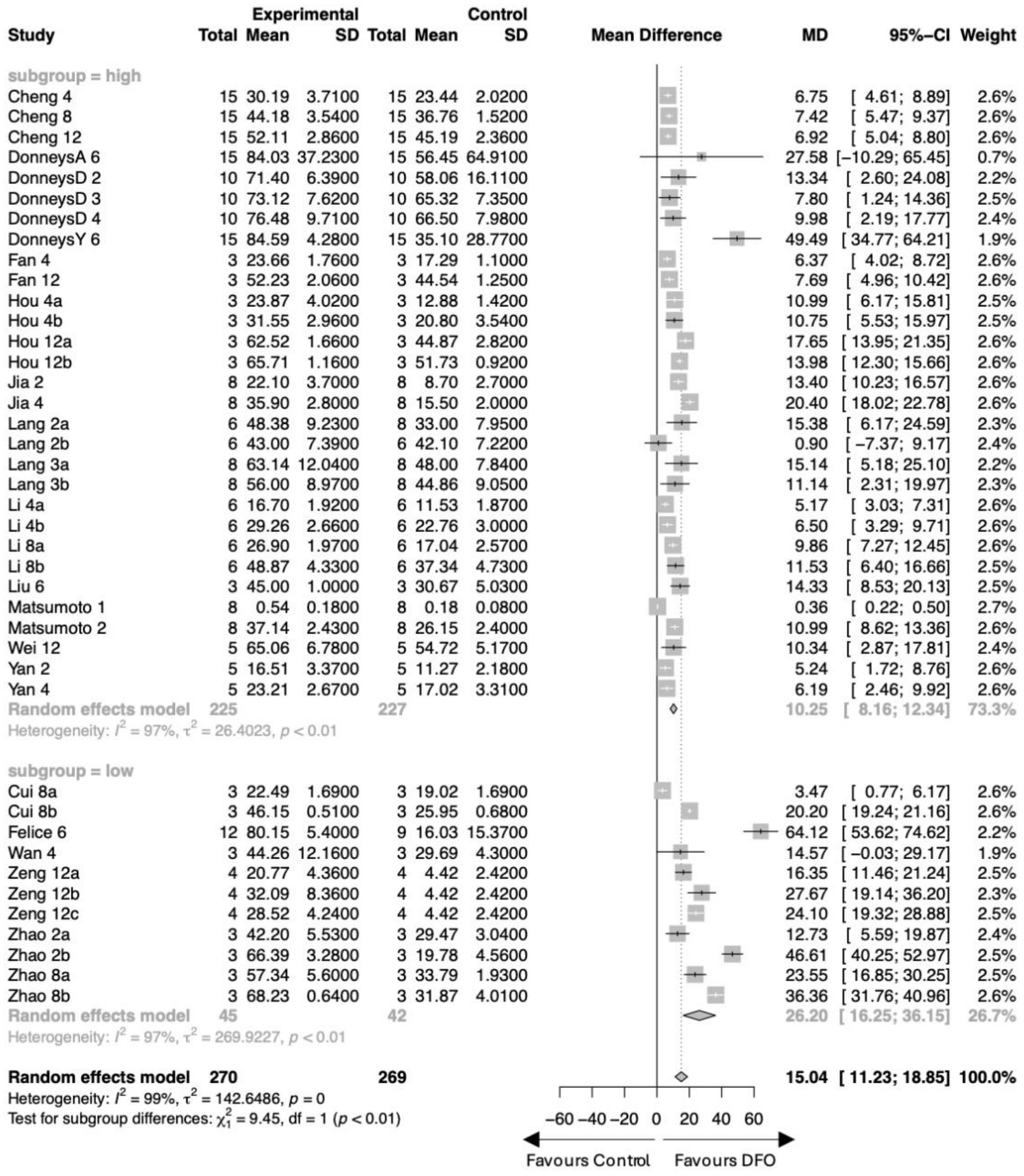


***Forest plot for the sensitivity analysis excluding low-quality studies***

***Figure S15:*** Sensitivity analysis for the influence of small studies

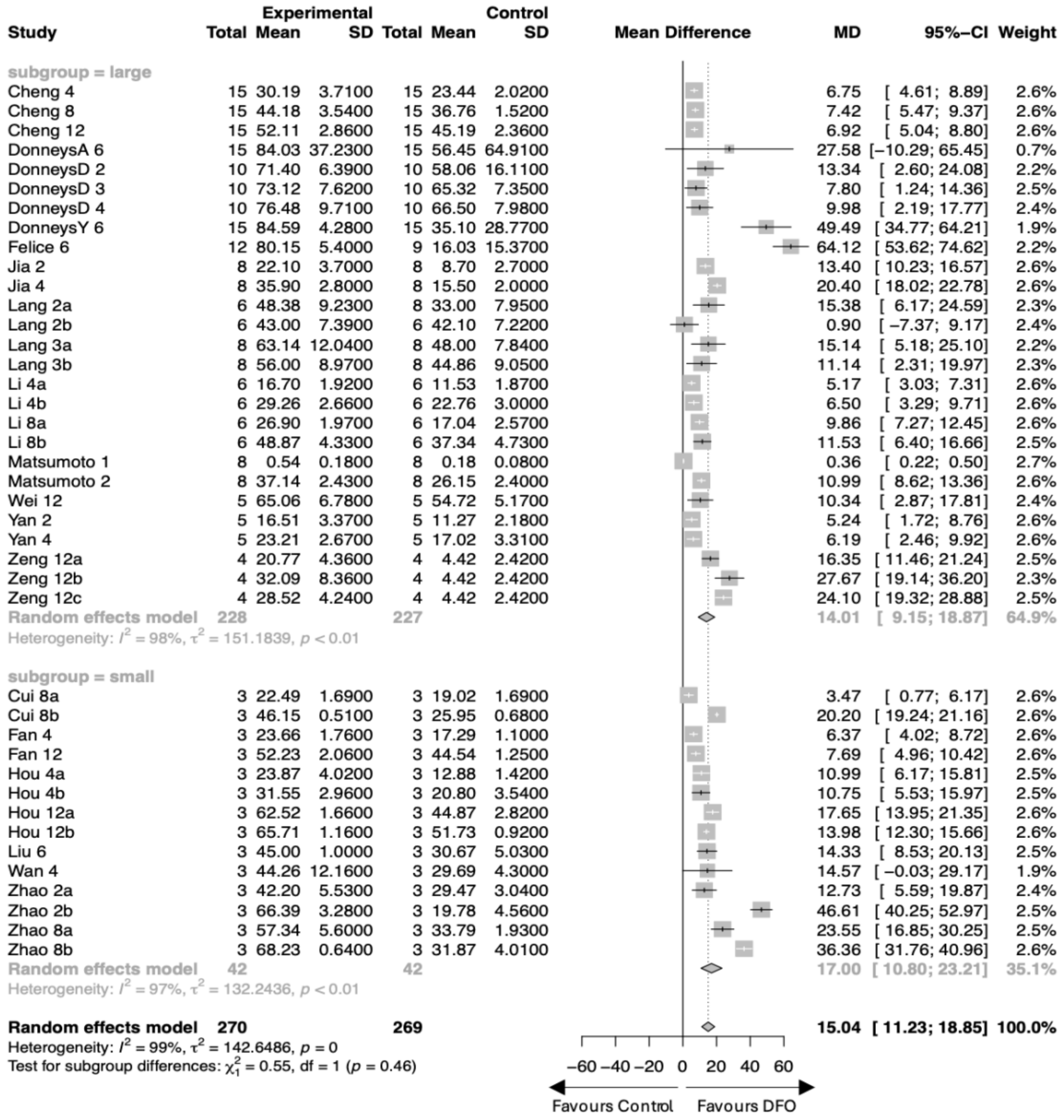


***Forest plot for sensitivity analysis excluding small studies***

***Figure S16:*** Sensitivity analysis for the influence of small studies further subdivided into small, moderate and large.

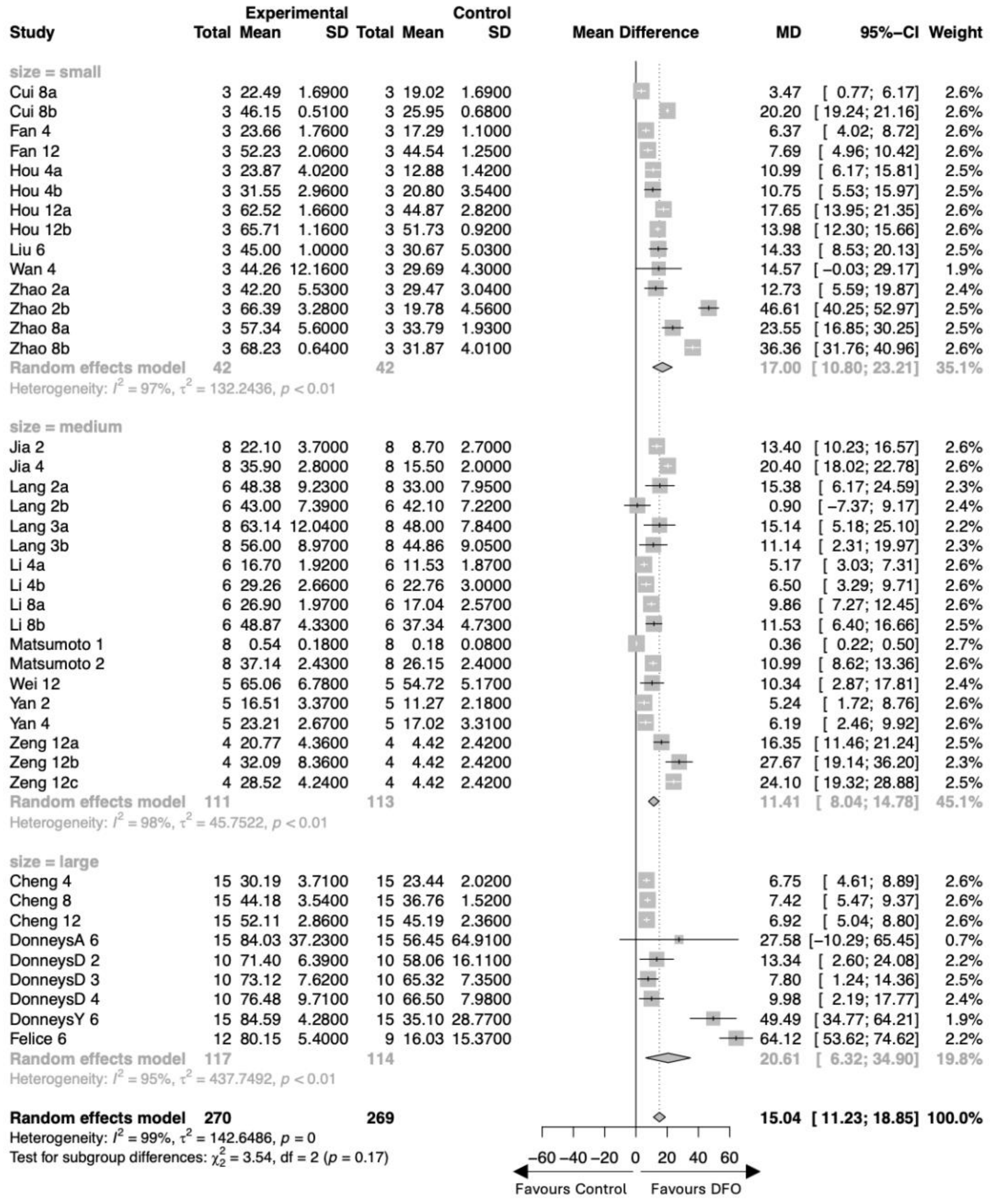


***Forest plot for sensitivity analysis investigating study size: categorized into small, moderate and large studies.***

***Figure S17:*** Subgroup analysis for the different brands and models of the used µCT device measuring BV/TV

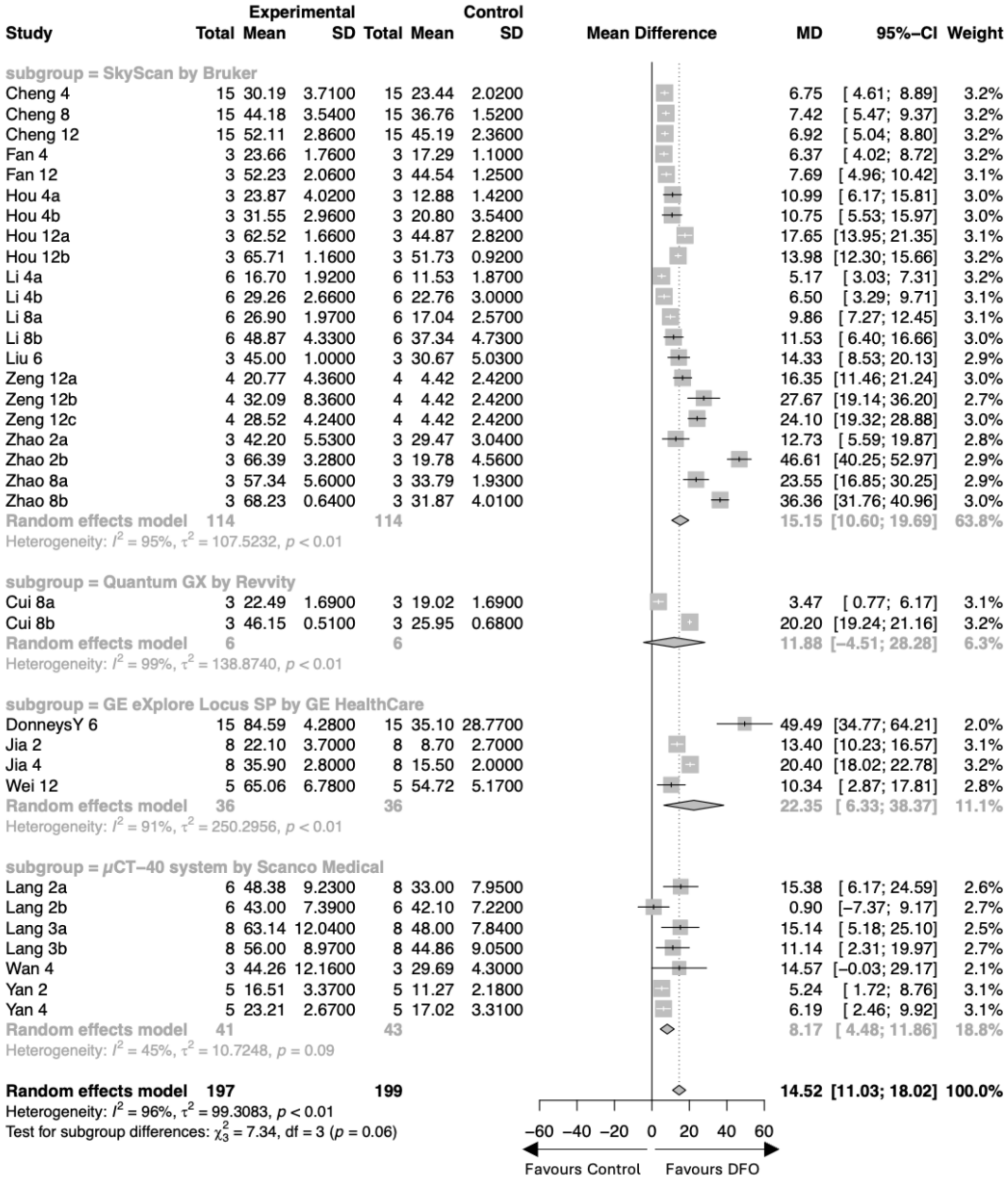


***Forest Plot showing a subgroup analysis for the different µCT device systems seen as potential confounder.***

## Publication Bias

***Figure S18:*** Investigating publication bias using the Trim-and-Fill method

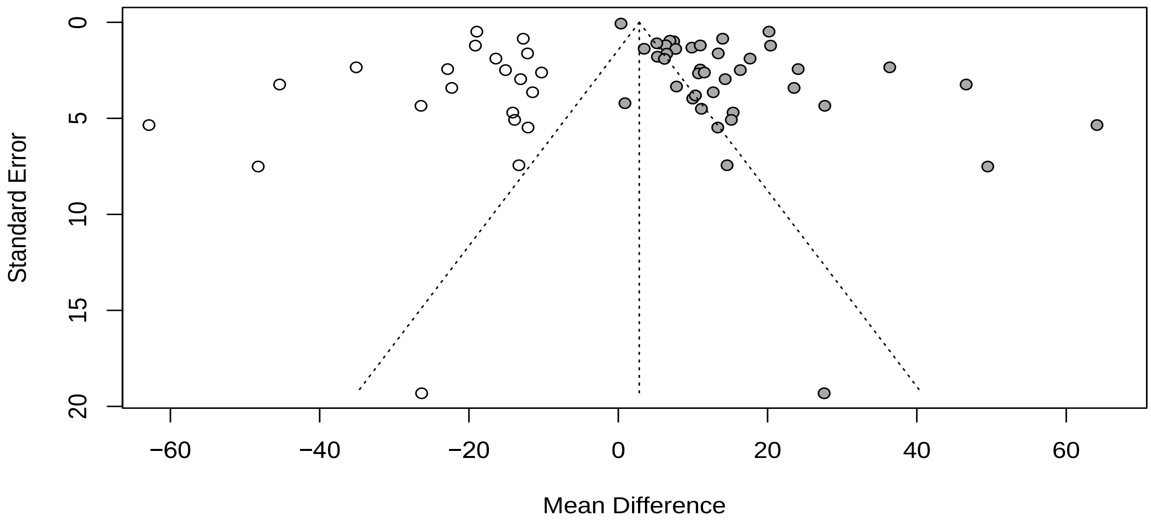


***Funnel plot presenting the adjusted distribution of the study results in our meta-analysis, identifying and adjusting for potentially missing studies (Trim-and-Fill).***

***Figure S19:*** Investigating publication bias with the exclusion of small studies presented in a funnel plot.

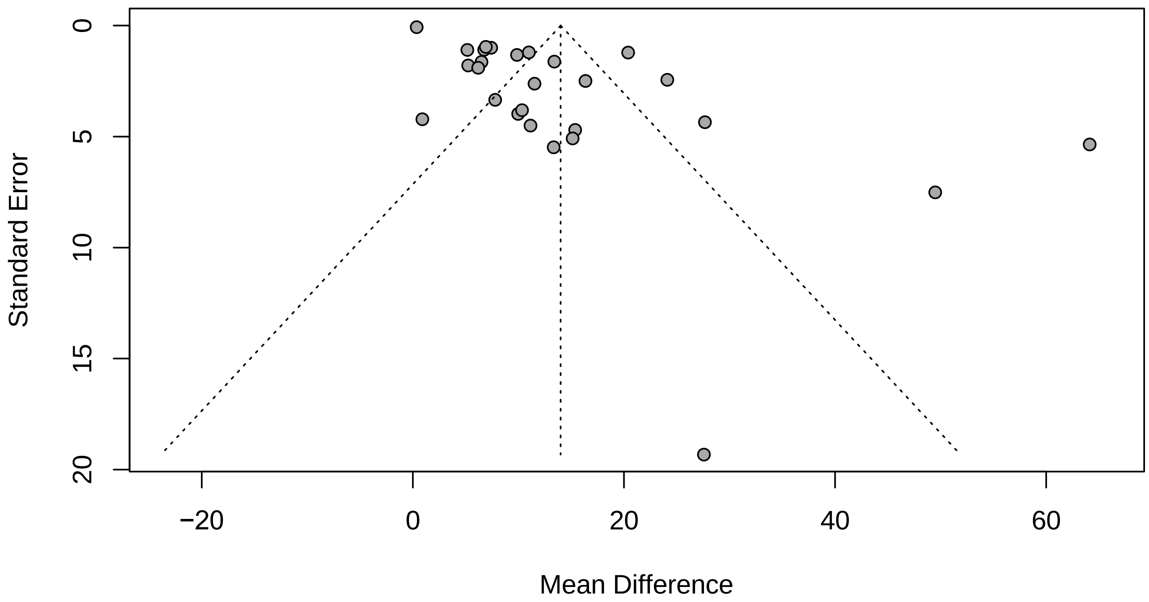


***Funnel plot with exclusion of small studies presenting the distribution of the study results in our meta-analysis, with correlating MD and standard error (SE) of BV/TV data.***

***Figure S20:*** Investigating publication bias using the Trim-and-Fill method with the exclusion of small studies

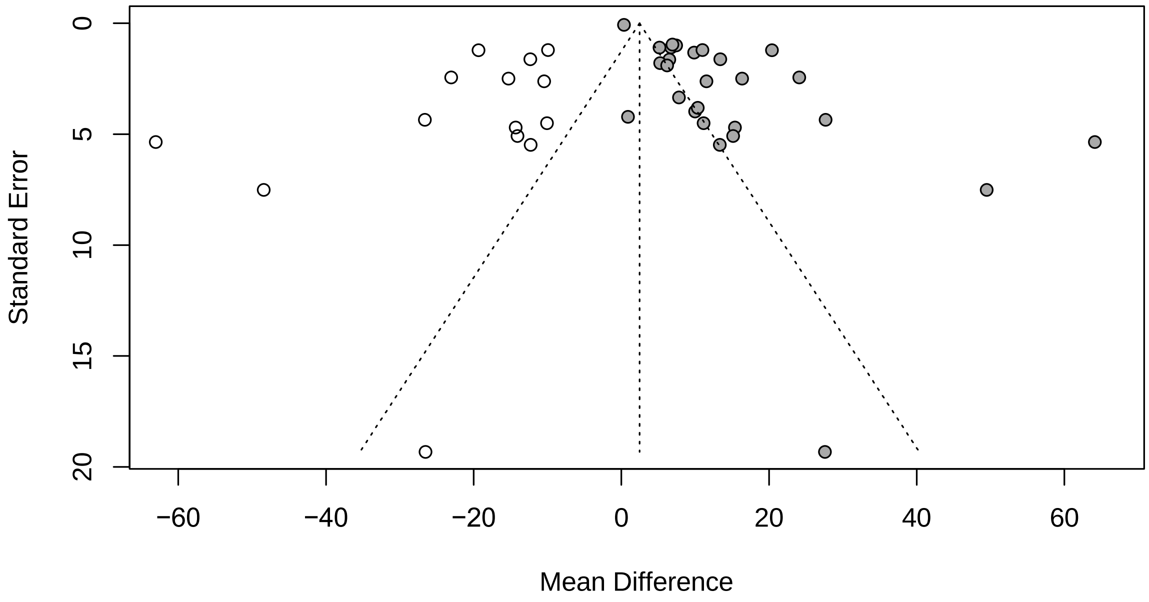


***Funnel plot presenting the adjusted distribution of the study results in our meta-analysis, identifying and adjusting for potentially missing studies (Trim-and-Fill) with the exclusion of small studies.***

## Adjusted Search Strategy

***Text S1:*** Adjusted search strategy for WOS (Web of Science)

(TI=(Deferoxamine or DFO* or Desferoxamine or Desferrioxamine or Desferal) AND (TI=(bon* or fractur* or skelet* or callus) AND TI=((defect* or seperat* or heal* or fusion* or regenerat* or remodel* or repa* or reconstruct* or mass or union or vasculariz* or format* or gap) or distraction osteogenesis)) OR AB=(Deferoxamine or DFO* or Desferoxamine or Desferrioxamine or Desferal) AND (AB=(bon* or fractur* or skelet* or callus) AND AB=((defect* or seperat* or heal* or fusion* or regenerat* or remodel* or repa* or reconstruct* or mass or union or vasculariz* or format* or gap) or distraction osteogenesis)) AND TS=(rat or rats or mouse or mice or murine))

*"*" (asterisk): Substitute for any string of zero or more characters;* ***"TI"*** *(Title): Searches within the title of the study;* ***"AB"*** *(Abstract): Searches within the abstract of the study;* ***"TS"*** *(Topic): Searches within the study's title, abstract, author keywords, and Keywords Plus (a proprietary keyword field created by WOS).*
